# A pragmatic workflow for integrating large language models into toxicological risk assessment

**DOI:** 10.3389/ftox.2026.1877128

**Published:** 2026-09-28

**Authors:** Arianna Bassan, Alexander Amberg, Maria Letizia Barreca, Paul M. Bradley, Frank Bringezu, Joyce De Paula Souza, Corinne Guicheney, Elena Lo Piparo, Simona Kovarich, Andrey Massarsky, Georgia Maundrill, Lance Molnar, Cristina Parenti, Patricia Parris, Manuela Pavan, John F. Reichard, Ron Steigerwalt, Olga Tcheremenskaia, Kenneth Unice, Christopher J. Waine, Geraldine Whelan, Angela White, Glenn J. Myatt

**Affiliations:** 1 Innovatune srl, Padova, Italy; 2 Preclinical Safety, Sanofi, Frankfurt, Germany; 3 In Silico Drug Discovery Lab (I2D Lab), Department of Pharmaceutical Sciences, University of Perugia, Perugia, Italy; 4 Paul Bradley Consulting, Royston, United Kingdom; 5 Merck Healthcare KGaA, Darmstadt, Germany; 6 Swiss Centre for Applied Human Toxicology, Department of Pharmaceutical Sciences, University of Basel, Basel, Switzerland; 7 Global Toxicology Services, KaLy-Cell, a Company of GBA Pharma, Plobsheim, France; 8 Global Regulatory Affairs, Sciences & Strategy, FMC, Geneva, Switzerland; 9 Global Regulatory & Computational Toxicology Team, Hazard Communication and Chemicals Regulations, Merck Life Science S.r.l., Milan, Italy; 10 Stantec Consulting Services Inc., Irvine, CA, United States; 11 Medicines Research Centre, GSK, Stevenage, United Kingdom; 12 Global Pharmacology and Toxicology, Viatris, Morgantown, WV, United States; 13 Environment and Health Department, Istituto Superiore di Sanità, Rome, Italy; 14 Pfizer Worldwide Research, Development and Medical, Sandwich, United Kingdom; 15 Department of Environmental and Public Health Sciences, University of Cincinnati, Cincinnati, OH, United States; 16 Ron Steigerwalt Regulatory Tox Consulting, LLC, Palm Springs, CA, United States; 17 Strategic Health Sciences, TRC Companies, Pittsburgh, PA, United States; 18 Bibra Toxicology Advice and Consulting, Wallington, Surrey, United Kingdom; 19 Informence Labs LLC, St. Petersburg, FL, United States

**Keywords:** AI, human-in-the-loop, LLM, PDE, pragmatic workflow, toxicological risk assessment

## Abstract

Artificial intelligence (AI), particularly in the form of large language models (LLMs), offers promising support for toxicological risk assessment (TRA), which integrates multiple lines of evidence to support health-based conclusions, including those used in regulatory decision-making. Effective use of LLMs in TRA requires a clear understanding of their strengths and limitations, especially in the context of regulatory expectations for transparency, reproducibility, and accountability. This work explores the emerging role of LLMs in TRA through three integrated components: an overview of LLMs in the context of TRA, an exemplar case study examining LLM-assisted derivation (mainly using ChatGPT) of the Permitted Daily Exposure (PDE) for acetyl tributyl citrate under the draft ICH Q3E guideline, and a workflow hypothesis for the integration of LLMs into TRA. Observations from the case study suggest that LLMs may support pattern recognition, targeted information retrieval, and rapid summarization of large bodies of text, facilitating literature triage, extraction of study details, and evidence summaries. At the same time, the case study highlights important limitations for LLMs. Observed variability in LLM outputs reflects inherent model stochastic behavior, model limitations, and the intrinsic complexity of TRA, where different experts may reasonably reach distinct, yet scientifically defensible conclusions based on the available evidence. The observations from the case study, interpreted in the context of the available literature, are used to inform a hypothesis that LLMs may be most effectively integrated into TRA through structured, modular, human-supervised workflows. This hypothesis reflects the view that the suitability of LLM-assisted TRA depends not only on LLM capabilities, but also on how LLMs are integrated into the assessment process to address both LLM variability and the interpretive nature of TRA. The proposed pragmatic workflow separates data search, information extraction, and integrated summarization while maintaining human-in-the-loop control over interpretation, expert judgment, and final conclusions.

## 1 Introduction

Artificial intelligence (AI) is increasingly used in toxicology. While traditional machine learning techniques have supported predictions of toxicity endpoints such as bacterial mutagenicity for a long time (Myatt et al., 2018; Cronin and Yoon, 2019; Hasselgren et al., 2019), recent advances in deep learning have broadened AI applications beyond predictions to tasks involving data integration and pattern recognition. These developments are particularly relevant for modern toxicology, which relies on large volumes of both structured and unstructured evidence (e.g., databases, full-text study reports, and narrative assessments) from diverse sources, including legacy studies, new approach methodologies (NAMs), high-throughput assays, omics platforms, and peer-reviewed literature (Hartung, 2023b; 2023a; Kleinstreuer and Hartung, 2024). AI methods can help manage this volume and heterogeneity of information, supporting predictive modeling, probabilistic risk assessment, and systematic evidence integration and summarization. Consequently, the mindset in chemical safety evaluation has shifted from “if” AI will be used to “how,” with growing consensus that AI will play a central role in modernizing risk assessment frameworks (Hartung et al., 2025b; Luechtefeld and Hartung, 2025).

Within regulatory science, AI is being explored to support the implementation of NAMs, as reflected by pilot studies of AI-based tools conducted by agencies such as the U.S. Food and Drug Administration (FDA), European Medicines Agency (EMA), European Food Safety Authority (EFSA), and Swissmedic (Thakkar et al., 2023; EMA, 2024; EFSA, 2025; FDA, 2025; GSRS25, 2025; Swissmedic, 2025; Tsaioun et al., 2025). Notably, the term AI does not describe a single, uniform technology, rather it comprises a spectrum of computational methods with varying levels of autonomy and human oversight (Sarker, 2022; CRS, 2025). Tong and Baran (2024) characterize this continuum as “50 shades of AI”, including many degrees of involvement, such as assisting intelligence that automates routine tasks, augmenting intelligence that enhances human decision-making, autocratic intelligence that operates with minimal human guidance, and autonomous intelligence operating independently with no human intervention. In the context of regulatory science, and particularly TRA, current AI applications are best understood as examples of assisting or augmenting intelligence, i.e., tools that improve efficiency and support evidence integration, while leaving ultimate responsibility and judgment with human experts.

Within this broader AI revolution, large language models (LLMs) represent a particularly promising development. General-purpose, instruction-tuned LLMs deployed in systems such as ChatGPT (OpenAI, San Francisco, CA, United States), Gemini (Google DeepMind, Mountain View, CA, United States), or Claude (Anthropic, San Francisco, CA, United States) have gained rapid popularity due to their ability to generate human-like responses to increasingly complex queries (Artificial Analysis, 2026). Their broad adoption by the public, business, education, research, and healthcare is further enabled by low barriers to access and minimal upfront costs for end-users in some applications.

LLMs are a type of Generative Artificial Intelligence (GenAI) that use a model architecture (often referred to as transformer models) designed to generate text responsive to user prompts (Vaswani et al., 2017). These models are trained using deep learning methods on billions of data points drawn from diverse sources, including websites, articles, and books collected over a finite timeframe. Through this training process, LLMs learn patterns in language, such as grammar, context, semantics, and reasoning. LLMs generate sentences one token at a time, where tokens may represent full words or word fragments. At each step, the model predicts the next token based on preceding text, using statistical likelihood rather than factual correctness as the guiding principle. The transformer architecture enables this by employing attention mechanisms that weigh the relevance of different parts of the input during generation (Vaswani et al., 2017). Because the generation process includes a degree of randomness, outputs may vary across runs of the same prompt, which can be beneficial for certain tasks (e.g., creative generation) (Holtzman et al., 2019). After pre-training, additional post-training and deployment-level techniques Zhang et al. (2025b) including fine-tuning, prompt engineering, retrieval-augmented generation (RAG), or rule-based alignment may be layered to refine model behavior. These layers help produce contextually appropriate, human-like responses while reducing the likelihood of biased or harmful outputs (Chang et al., 2024).

Based on their ability to model language, semantic context, and relationships, LLMs hold significant potential to support many tasks common in TRA (Kleinstreuer and Hartung, 2024; Parisi and Sutton, 2024; Sonnenburg et al., 2024; Moreira-Filho et al., 2025; Schmidt et al., 2025). These include drafting reports, summarizing toxicological studies, literature searching, and extracting and collating text across large volumes of documents. When strategically integrated, LLMs can streamline and accelerate assessments while maintaining scientific rigor and supporting robust conclusions. In this respect, the value of LLMs lies not in replacing expert toxicologists, but as a tool for augmenting expert judgment through hybrid human-AI approaches, thereby enhancing transparency, reproducibility, and accountability (ECETOC, 2025; Hartung et al., 2025b). By accelerating data integration and evidence summarization, LLMs can support subject matter experts in TRA and contribute to the 3Rs (Replacement, Reduction, and Refinement) by reducing reliance on new animal studies (Kleinstreuer and Hartung, 2024; Lui, 2024; Alam et al., 2025). However, their use in TRA is challenged by their lack of domain-specific training and intrinsic limitations including prompt sensitivity, output variability, limited explainability, and biases.

This manuscript presents the perspectives of a collaborative working group on the emerging role of LLMs in TRA and is organized into three parts: (i) study foundations, (ii) an exemplar case study including exploratory LLM investigations and corresponding observations, and (iii) a workflow hypothesis informed by the study foundations, case study, and resulting observations (Figure 1). First, the study foundations are established in the background section, which provides an overview of LLMs in the context of TRA, outlining their core principles, strengths, and limitations, and highlighting both their transformative potential and current challenges based on the literature and the working group’s perspectives. Second, a case study examines the use of LLMs to support the derivation of a Permitted Daily Exposure (PDE) for acetyl tributyl citrate involving several core elements of TRA; PDE derivation integrates toxicological information to estimate a daily chemical dose not expected to pose a health risk. The case study serves as an exemplar to explore the use of LLMs in TRA and to generate observations on their applications to tasks including evidence retrieval, summarization, and reasoning. It is not designed as a validation exercise, but rather as a means to inform the development of the proposed workflow hypothesis. The observations from the case study are interpreted in the context of the available literature. Third, insights from the background section and the observations from the case study are used to propose an LLM-powered workflow that positions LLMs as augmentative tools to support, rather than replace, expert judgment. This workflow follows a modular, human-supervised approach in which LLMs are selectively applied to support specific tasks. Together, the study foundations, exemplar case study, and proposed workflow support the hypothesis that one possible fit-for-purpose LLM use in TRA is achievable when models are incorporated into transparent, human-supervised workflows grounded in verifiable, case-specific evidence. The methodological contribution of this work lies in translating observations from an LLM-assisted PDE case study into a structured, regulator-aligned workflow hypothesis that operationalizes human-in-the-loop use of LLMs in TRA.

**FIGURE 1:**
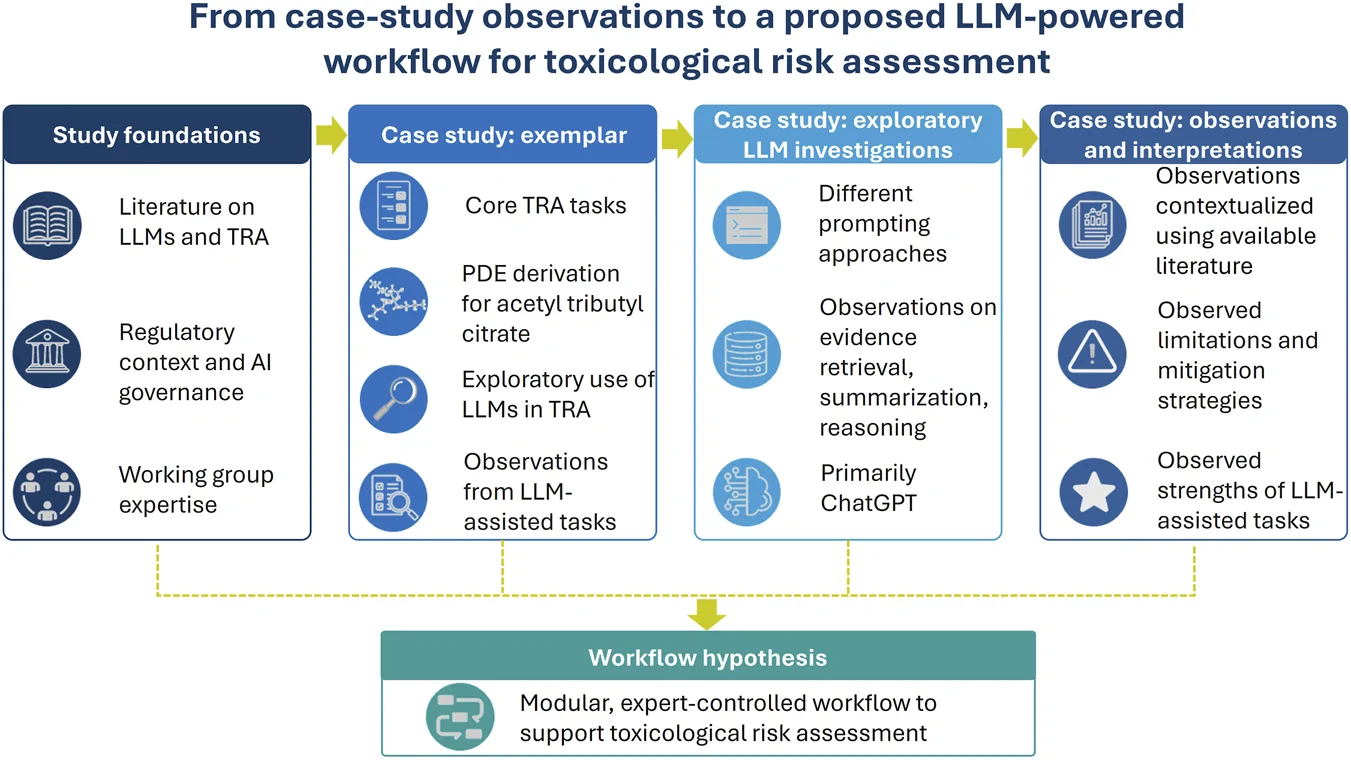
From case-study observations to a proposed LLM-powered workflow for TRA. The study foundations comprise a background analysis of LLMs in the context of TRA, the regulatory context, and the perspectives of the working group. An exemplar case study on the derivation of a Permitted Daily Exposure (PDE) for acetyl tributyl citrate was then conducted. Exploratory LLM investigations generated observations on the use of LLMs for core TRA tasks, including evidence retrieval, summarization, and reasoning. These observations were interpreted in the context of the available literature to identify both strengths and limitations of LLM-assisted approaches and to inform potential mitigation strategies. Together, the study foundations, case-study observations, and their interpretation informed the workflow hypothesis that LLMs may be most appropriately integrated into TRA as components of structured, modular, human-supervised workflows that support, rather than replace, expert judgment. Exploratory investigations were conducted primarily using ChatGPT, with selected Claude and Gemini runs reported in the Supplementary Material.

## 2 Background: LLMs and TRA

### 2.1 Technical considerations for the use of LLMs

LLMs are designed as general-purpose systems and trained primarily on data aggregated from broad, non-specialized sources rather than domain-specific sources (Busch et al., 2025). LLMs generate outputs predicting the most statistically likely next tokens based on learned patterns in text, rather than by applying domain-specific reasoning and logic (Vaswani et al., 2017; Radford et al., 2019). Notably, LLMs often rely on keywords and familiar phrasings rather than robust domain reasoning, a phenomenon known as shortcut learning (Geirhos et al., 2020; Du et al., 2024). Beyond the domain gap, LLMs exhibit some technical characteristics and challenges that are important to consider when applied in scientific and regulatory settings.

#### 2.1.1 Inaccuracies and omissions

LLMs may generate incorrect or fabricated statements, a phenomenon commonly referred to as “hallucination” in the AI literature (Ji et al., 2023; Sun et al., 2024), but more accurately described as confabulation, referring to the generation of plausible but false content without awareness of its inaccuracy or any intent to deceive (Smith et al., 2023). Several factors contribute to this type of error. The underlying training data may lack peer review, contain outdated information, or include factual inaccuracies. Even when explicit source material is provided for summarization, LLMs may still rely on other learned patterns and introduce errors. Owing to the nature of machine learning and ranking methods, a pattern or trend may reflect a statistical association without being causally or contextually valid. Furthermore, the ranking algorithms of these systems often favor generating a response even if the data are limited or unreliable rather than declining to respond (Kalai et al., 2025) or indicating that the model does not have sufficient information needed to provide an adequate response. These artifacts can introduce or reinforce biases in the output that the LLM filters do not detect. Since the knowledge encoded in general-use LLMs reflects only the documents included in their training sets, important information from missing documents or more recent publications may be omitted entirely. Since the output from LLMs may contain inaccuracies, omissions, or biases, many of the commercially available LLMs explicitly recommend LLM outputs to be reviewed by users, especially in situations that support important tasks. LLMs are also reported to be prone to errors in quantitative reasoning and numeric extraction, thus requiring additional verification of the output (Frieder et al., 2023).

Methods for reducing hallucinations have been developed (Anh-Hoang et al., 2025; Joshi, 2025; Li et al., 2025; Lin et al., 2025). One widely used approach is RAG, which constrains the model’s responses to external sources, linking outputs to verifiable documents and reducing unsupported claims. Prompting strategies that encourage the model to reason step-by-step or check its conclusions for internal consistency (often called “chain-of-thought” prompting) can also improve accuracy and logical coherence. More recently, agentic systems incorporate automated post-generation checks, including secondary verification steps and tool-based validation (Darwish et al., 2025). These approaches can significantly reduce hallucinations, although they require more complex system architectures. Under constrained tasks with structured outputs and expert review, hallucination rates near 1% have been reported (Asgari et al., 2025).

#### 2.1.2 Prompt engineering

Prompt engineering is the practice of designing and optimizing prompts (instructions) to guide GenAI models to produce accurate and relevant outputs. Prompt engineering facilitates domain-specific applications of LLMs by systematically encoding domain knowledge, task definitions, and workflow constraints into input prompts. Because LLMs generate text in direct response to user prompts, the quality and reliability of the output are highly dependent on how these prompts are constructed. Crafting an effective prompt can be challenging, as it may need to specify the task context and provide a clear, unambiguous request, while also detailing requirements such as response length, format, or tone. Moreover, it is often uncertain whether a particular prompt will yield an appropriate response or whether including illustrative or clarifying examples will enhance the quality of the output (Bsharat et al., 2023). Prompt engineering (i.e., crafting domain-appropriate, well-scoped questions) has been shown to improve retrieval coverage and correctness of answers (Li D. et al., 2024; Qiu and Zhou, 2024; Wang et al., 2024). Importantly, careful prompt design serves as a key safeguard against hallucinations (confabulations), because constraining outputs through explicit instructions reduces the likelihood of fabricated or misleading content (Moreira-Filho et al., 2025). At the same time, because LLM outputs are sensitive to prompt formulation, transparent reporting of prompts is important, and established methodologies for evidence evaluation remain crucial (Kosch and Feger, 2025).

#### 2.1.3 Consistency

LLMs incorporate stochastic processes in their transformer-based architectures, resulting in different responses to the same prompt. In many cases, these variations are superficial (e.g., different wording with the same semantic meaning) but this is not always true. A single token choice during generation can shift the meaning of a sentence, leading to materially different outputs. For example, the model may select a word partway through the generation process that carries a different meaning or connotation than alternative choices, which can influence the selection of subsequent words and result in a divergent semantic path. Variability may also arise when new versions of an LLM are released. These updates, which may include changes to model architecture or training data, are not always visible to end users and can produce different results for identical prompts. Beyond run-to-run variability, studies have documented broader concerns about LLM stability, particularly their tendency to provide different answers when presented with slightly rephrased but semantically equivalent questions (Qiu and Zhou, 2024; Wang et al., 2024). This inconsistency driven by non-determinism and sensitivity to prompt-phrasing has been documented in the literature through systematic evaluations, including within the health sciences (e.g., Busch et al., 2025; Ouyang et al., 2025; Qiu and Zhou, 2024; Shiferaw et al., 2024; Wang et al., 2024; Zhu et al., 2024).

#### 2.1.4 Explainability

LLMs contain billions of parameters learned from vast training datasets, and this scale makes it impossible to directly trace their reasoning processes or to determine the factual basis and reliability of specific inferences or statements. Moreover, the full set of documents used to train these models is often inaccessible as some sources may be proprietary or non-public, making independent verification of outputs difficult (Zhang and Zhao, 2025). Although LLMs may produce fluent step-by-step explanations, these are often post-hoc rationalizations rather than faithful representations of the model’s internal decision process (Parcalabescu and Frank, 2024). Despite these limitations, LLM outputs can still be useful when guided by effective prompt design and critically interpreted by subject-matter experts, enabling the generation of information suitable for assessment and decision-support.

In response to the limited transparency of complex AI models such as LLMs, explainable artificial intelligence (xAI) encompasses a range of techniques designed to provide human-interpretable insights into model outputs and behavior (Alkhanbouli et al., 2025). xAI does not seek to fully reconstruct the internal reasoning of highly parametrized models (a task that is generally not feasible), but instead relies on post-hoc or model-intrinsic methods to highlight influential features, decision pathways, or local approximations of model behavior (Hartung, 2023a; Hartung et al., 2025a).

#### 2.1.5 Bias

Biases can arise intrinsically from the training data itself (e.g., imbalanced or non-representative sources) or extrinsically during downstream tasks, where outputs may amplify selective narratives or omit critical evidence (Guo et al., 2024). Biases may also be introduced intentionally by model developers or deployers, which can shape model behavior in line with specific governance, safety, or preferences (Bommasani et al., 2021; Ouyang et al., 2022) as well as the wording and structural composition of the prompt (Brucks and Toubia, 2025; Gandhi and Gandhi, 2025). In toxicology, for example, such biases could distort evidence retrieval, underrepresent negative findings, or misinterpret regulatory frameworks, thereby undermining the reliability of safety assessments and underscoring the need for active bias assessment and expert review (Hartung et al., 2025a).

#### 2.1.6 Validation

Validation refers to determining whether a model performs reliably and is fit for its intended purpose. For LLMs, this means assessing whether the generated text is accurate, appropriate, and safe for the specific task and context (Hond et al., 2024). Validation is particularly challenging for LLMs because they can produce multiple diverse outputs for the same query, complicating benchmarking and reproducibility (Alam et al., 2025). As a result, LLM validation remains an active area of research and practice. It can be approached through structured, task-based evaluations aligned to the intended use (Hond et al., 2024) and/or as a continuous lifecycle process that includes ongoing monitoring, drift detection, and human oversight rather than a one-time approval (Luechtefeld and Hartung, 2025). In practice, domain-specific metrics (e.g., factuality, faithfulness to cited sources, harmful content rates, quantitative accuracy) are often needed alongside general measures of quality.

#### 2.1.7 Context handling and document processing

The context window is the LLM’s working memory pertaining to the user’s current prompt, conversation history, system instructions and uploaded documents; it is essentially the maximum amount of information an LLM can “keep in mind” and process during a single interaction (Vaswani et al., 2017; Lewis et al., 2021; Liu et al., 2023). Complex interactions with LLMs that rely on large datasets, including multiple documents, can be limited by the size of the context window, which is measured in tokens. When the number of tokens accrued during an LLM interaction exceeds the context window, the model may “forget” details of the conversation, causing responses to become less relevant, less coherent, and more repetitive or generic (Liu et al., 2023). Different LLMs handle uploaded documents differently, which can impact the context window and document parsing and representation. LLMs do not “read” uploaded documents; instead, they use various approaches to identify and score the most relevant sections of a document and feed selected content into the context window to generate output. Direct ingestion of entire documents is inefficient because the full document counts against the context window regardless of relevance. Retrieval-based approaches such as RAG circumvent this inefficiency, as discussed below.

#### 2.1.8 Data security and privacy

LLMs are typically hosted in cloud-based data centers and accessed over the internet. Transmitting confidential information, such as sensitive data or proprietary business materials, to these systems requires additional safeguards, including enhanced security controls or redaction of sensitive content before submission.

### 2.2 Implementation strategies for LLMs

Most LLMs are pre-trained on vast datasets of generic text data rather than domain-specific sources. While this provides broad language capabilities, it can limit the performance in specialized fields including TRA, for example, by misinterpreting or oversimplifying key concepts or failing to recognize relevant regulatory frameworks. These limitations make the manner in which LLMs are integrated into contexts such as TRA particularly important, as different approaches carry distinct implications for interpretability, methodological control, and regulatory suitability.

LLMs, such as the Generative Pre-trained Transformer (GPT) family developed by OpenAI (OpenAI, 2026), the Claude family developed by Anthropic (Anthropic, 2026) and Gemini developed by Google (Gemini Team, 2025), are backend models that generate text in response to input and are typically accessed via an Application Programming Interface (API). On their own, these models do not manage conversations, retain context beyond the provided input, or enforce specific rules. Chatbots, such as ChatGPT (OpenAI), Claude (Anthropic), Gemini (Google), and Microsoft Copilot (which incorporates OpenAI models), are instead user-interface applications built on top of LLMs (IBM, 2021; Casheekar et al., 2024; Dam et al., 2024). In addition to embedding the underlying LLM, chatbots implement conversation management (including context window handling and memory), system instructions (pre-configured prompts that shape responses but are invisible to users), safety controls, and sometimes extended functionalities such as web search, code execution, or access to external databases. As a result, LLM behavior can differ between chatbot-based deployments and API-based access, reflecting not only the underlying model but also application-level design choices that affect how the output is generated.

Retrieval-based approaches are an important implementation in specialized contexts such as TRA because chatbots do not guarantee that outputs are grounded in explicit evidence. RAG links LLM text generation to an explicit retrieval step from a curated knowledge base, thereby constraining outputs to identifiable source material (Lewis et al., 2021). When working with very large files or multiple files, it is more efficient to “chunk” the text stored in a vector database, based on semantic meaning (i.e., embedding). RAG approaches retrieve relevant chunks to the context window to generate output. RAG helps avoid cluttering the context window with unnecessary noise that occurs with direct ingestion when information is uploaded to the chat window. This helps the LLM focus on the main conversation points and retain pertinent information in the context window. RAG improves factual specificity, reduces reliance on potentially outdated or implicit model-encoded knowledge (Abo El-Enen et al., 2025; Gan et al., 2025; Ke et al., 2025; Zhang et al., 2025a), and enables traceable outputs that are more suitable for transparent and reproducible TRA (Kleinstreuer and Hartung, 2024; Sonnenburg et al., 2024). However, RAG primarily governs the information input and does not, by itself, enforce the logical structure or sequencing expected in established toxicological methodologies.

Orchestration-based systems act as a conductor for LLM-based workflows, coordinating multiple components and integrating LLMs into predefined, stepwise processes aligned with domain-specific paradigms. Orchestration regulates when and how LLMs are invoked, enforces the use of approved sources, specifies task order, embeds decision checkpoints, and incorporates expert review (e.g., requiring evaluation of available systemic toxicity data before derivation of compound-specific limit). In such systems, the workflow is fixed, even though the LLM-generated content within each step may show variability. Supervised, modular workflows of this type are increasingly recognized as a pragmatic way to integrate LLMs into high-stakes domains (e.g., regulatory risk assessment) where transparency, reproducibility, and expert supervision are essential (Huang et al., 2024; Zou et al., 2025).

Beyond RAG, which focuses outputs on explicit evidence, and orchestration, which governs workflow sequencing, additional rigor can be achieved by formally encoding domain knowledge and constraints within the system architecture. Domain-Driven Design (DDD) operates at this deeper architectural level by explicitly modeling a specific domain (e.g., the toxicological domain) and its core concepts. Unlike orchestration approaches that control the sequence of assessment steps through an external workflow engine, DDD embeds core toxicological rules and validity constraints directly into the system architecture. This allows LLM-assisted outputs to be automatically checked against established domain-specific principles. By defining key domain concepts, relationships, and constraints as formal elements of the software design, DDD provides structured representations that help manage the inherent non-determinism of LLM outputs and enable systematic validation against domain logic (Evans, 2004; Ling et al., 2023). For example, in the TRA context, this could include fixed hazard evaluation sequences, dose–response criteria, or acceptable ranges for modifying factors (i.e., uncertainty factors) encoded as architectural rules. By making assumptions, constraints, and decision pathways explicit at the architectural level, DDD supports interpretability, traceability, and auditability of AI-assisted reasoning–capabilities that are essential under regulatory scrutiny (Mitchell et al., 2018; Bommasani et al., 2023).

Agentic LLM systems represent a distinct approach in which LLMs are used not only to generate text but also to plan and carry out multi-step tasks. In these systems, the model is given a high-level objective and can autonomously determine which actions to take, in what order, and which external data sources or analytical tools to invoke to achieve that objective (Hartung, 2025; Luechtefeld and Hartung, 2025; Plaat et al., 2025). A defining feature of agentic systems is tool integration, which may include document-retrieval engines, databases, calculators, modeling software, or other domain-specific tools that allow the LLM to iteratively retrieve information, perform analyses, and refine intermediate results (Djidrovski et al., 2025). Illustrative examples include ChemCrow, an LLM-based agent supporting chemical tasks such as synthesis planning, reaction analysis, or data gathering (Bran et al., 2024), and OpenAI’s Deep Research, which autonomously scans, evaluates, and synthesizes insights from hundreds of sources while documenting the steps of the process (OpenAI, 2025b; Xu and Peng, 2025). Unlike orchestration-based approaches, where task sequence and decision points are predetermined by developers, agentic systems allow the model to dynamically adjust the workflow based on intermediate findings. Architecturally, agentic systems differ fundamentally from RAG, orchestrated, or DDD-based approaches because they delegate control over task progression and tool invocation to the model itself, rather than prescribing these decisions through external workflows or embedded domain rules.

### 2.3 Toxicological risk assessment

TRA is a complex process and represents a particularly demanding application context for AI. In such settings, the use of LLM approaches must be carefully contextualized. TRA has long relied on the classic four-step paradigm (hazard identification, dose–response assessment, exposure assessment, and risk characterization) formalized in the U.S. National Research Council (NRC)’s “Red Book” (US NRC, 1983; Graham, 1995). Within this framework, TRA is defined as the process of characterizing potential adverse health effects resulting from human exposure to chemicals. In addition to data, TRA requires expert judgement to address problem formulation, identify and choose default assumptions, handle uncertainty and variability, and weigh multiple lines of evidence. The NRC has emphasized that such judgments are intrinsic to risk assessment, reflecting normative choices about protection goals, acceptable uncertainty, and decision context rather than purely objective scientific determinations (US NRC, 2009).

TRA has progressively expanded beyond the original four-step paradigm to incorporate more mechanistic, evidence-based, and computationally informed approaches. Mode-of-Action (MoA) frameworks describe biologically plausible sequences of events leading to observed effects and have informed the development of Adverse Outcome Pathways (AOPs), which link molecular events to apical outcomes at the organism or population level (Meek, 2008; Ankley et al., 2010; Villeneuve et al., 2014b; 2014a; OECD, 2017). Substantial advancements in TRA have been driven by the integration of NAMs, including *in vitro* assays, *in silico* methods, high-throughput screening programs (e.g., ToxCast/Tox21 (US EPA, 2015)), omics technologies, physiologically based pharmacokinetic (PBPK) models, and the Integrated Approaches to Testing and Assessment (IATA) (OECD, 2020). Next-Generation Risk Assessment (NGRA) is the latest adaptation of the original TRA paradigm combining data from high-throughput human-cell–based assays with computational models of bioavailability and distribution to evaluate health risks at relevant internal concentrations (Dent et al., 2018).

Collectively, these developments have strengthened evidence-based TRA frameworks towards more systematic, human-relevant, and data-rich evaluations moving away from animal testing methods (Krewski et al., 2022). As a result, the volume and complexity of information that must be summarized, integrated and interpreted have increased substantially. The increasingly diverse data sources generate heterogeneous evidence streams that challenge conventional review processes. Taken together, these trends suggest a meaningful role for LLMs: by processing large amounts of text and generating human-readable summaries and explanations, they can support literature review, extraction and structuring of study details, evidence mapping and documentation. Under appropriate expert supervision, they may also assist with certain aspects of data evaluation and mechanistic interpretation, making them potentially valuable contributors to modern TRA.

### 2.4 LLMs for toxicological risk assessment

#### 2.4.1 Key challenges

Conclusions from TRA have direct implications for human health and safety. Accordingly, integrating LLMs into TRA must account for the rigorous expectations placed on toxicologists and risk assessors regarding accuracy, transparency, reproducibility, and defensibility. Because TRA outputs directly inform regulatory decisions and public health protections, these qualities are fundamental requirements. Moreover, elements of TRA that rely on expert judgment (e.g., problem formulation, interpretation of uncertain or conflicting evidence, and the selection of protective assumptions (US NRC, 2009)) should not be fully delegated to automated systems without accountability.

Attention to transparency, explainability, accountability, and auditability is essential for building trust in the inclusion of LLMs in decision-making processes (Amann et al., 2020; Lötsch et al., 2021; Tucci et al., 2022; Balasubramaniam et al., 2023; Afroogh et al., 2024; Herrera-Poyatos et al., 2025; Luechtefeld and Hartung, 2025; Gant et al., 2026). These concepts are closely related. Transparency provides insight into the process (e.g., how outputs are generated and how decision pathways are constructed, including the information sources and methods). Explainability complements transparency and addresses the understanding of the outputs and the rationale underlying a given conclusion. Auditability and accountability relate to the traceability of processes and the clear attribution of responsibility for conclusions, respectively. Ultimately, toxicologists – not LLMs – are accountable for signing off on assessments and for their conclusions.

LLMs rely on architectures built from billions to trillions of parameters and highly non-linear operations, which makes it difficult to reconstruct how a specific input produces a particular output (Senoner et al., 2024). The detailed mechanisms that drive model behavior remain only partially understood, even by those who design and train these models (Nature Editorial, 2023). This challenge is often referred to as the “black box” problem (Castelvecchi, 2016; Samek et al., 2017; Rudin, 2019). Consequently, full transparency of LLMs is lacking and this limitation may undermine trust in LLM-assisted outputs for TRA. For this reason, the preference for explainable and understandable outcomes is a practical need in TRA. The importance of transparency, however, depends on the context of application: in exploratory tasks such as prioritization or hypothesis generation, LLMs can provide substantial value and time savings even with limited explainability, whereas in regulatory decision-making, transparency of reasoning is considered critically important.

Challenges associated with the use of LLMs are recognized by regulators. For example, the EMA notes that although simple and transparent models are generally preferred, more complex “black box” systems, such as LLMs, may still be acceptable when their use is well-justified and supported by clear documentation and ongoing monitoring (EMA, 2024).

#### 2.4.2 AI governance

AI governance frameworks have emerged to manage high-risk applications in which system failures could harm human health, safety, or environmental protection (EU, 2024; Papagiannidis et al., 2025). This framing is directly relevant to TRA, where AI-assisted outputs contribute to product safety determinations and regulatory decisions. Several international frameworks now provide structured approaches for governing such high-risk applications of AI (e.g., LLMs). For example, the European Union (EU) AI Act establishes regulatory obligations for high-risk AI systems, emphasizing transparency, documentation, human oversight, and accountability (EU, 2024). Similarly, the US National Institute of Standards and Technology (NIST) AI Risk Management Framework and its companion Playbook outline a structured approach organized around four core functions – Govern, Map, Measure, and Manage – for identifying and mitigating AI risks across domains, with a Generative AI Profile developed to address risks specific to LLMs and other generative AI systems (NIST, 2023; 2024; 2026). Complementing these frameworks, ISO/IEC 42001 provides a certifiable international standard for implementing AI management systems across the full AI lifecycle (ISO, 2023). The EMA and FDA have jointly released guiding principles for good AI practice in drug development, including human-centric design, a risk-based approach, adherence to standards, clear context of use, data governance and documentation, risk-based performance assessment (including human–AI interaction), and life-cycle management (EMA, 2026; FDA, 2026).

Governance frameworks consistently emphasize trustworthiness characteristics including accountability, transparency, explainability, and human oversight. These principles are widely recognized as essential when AI-assisted outputs may affect human health or regulatory determinations as reflected in various proposals such as the TRIAL framework (Transparency, Reproducibility, Interpretability, Applicability, and Liability) (Anklam et al., 2022) or the TREAT principle (Trustworthiness, Reproducibility, Explainability, Applicability, Transparency) (Hartung et al., 2025b).

Overall, AI is viewed as complementing and potentially augmenting human expertise, rather than replacing it. Its safe integration requires evolving governance frameworks and harmonized standards addressing both technical limitations (e.g., incomplete training data, hallucinations, biases, reproducibility) and regulatory risks associated with transparency, accountability, explainability, and trust (Lui, 2024; ECETOC, 2025; Gant et al., 2026). The widely cited maxim that “AI will not replace toxicologists, but toxicologists who do not use AI will be replaced by those who do” (Anklam et al., 2022) captures the practical implications of this governance perspective: LLMs should be deployed as augmentative tools, not autonomous decision-makers. This human-centered approach aligns with the EU’s ethics guidelines for trustworthy AI, which identify “human agency and oversight” as a foundational requirement (EU, 2024; 2025). Accordingly, AI systems used in TRA should empower experts to make informed judgments, supported by structured review processes often referred to as human-in-the-loop frameworks.

## 3 Case study: PDE derivation

### 3.1 Case study approach

As summarized in Figure 1, this work adopts a pragmatic perspective on the use of LLMs in TRA. Drawing on prior experience, existing literature, and a focused case study, it examines how LLMs can be integrated into LLM-powered workflows. Rather than presenting a statistical benchmarking study, a quantitative performance comparison, or a formal validation exercise, the objective is to generate observations regarding the practical use of LLMs in TRA and to examine how these observations may inform the development of a workflow hypothesis. The case study is intended to illustrate, through an exemplar application, how LLMs may function as components within TRA processes. Various prompting approaches and strategies are explored to assess practical behavior, but no systematic comparison across prompt configurations, models, or deployment modes is performed. Consequently, observations are aggregated and interpreted collectively rather than reported according to specific model settings or prompting configurations.

#### 3.1.1 Study design and rationale

The use of LLMs to support the derivation of a PDE is presented here as an exemplar of TRA. The case study focuses on the lifetime oral PDE for acetyl tributyl citrate (Table 1). A PDE represents a substance-specific daily dose considered unlikely to cause adverse effects when exposure occurs over a lifetime. Its derivation follows core TRA elements, namely, systematic evidence retrieval, critical evaluation of study quality and relevance, identification of a point-of-departure (PoD), and integration of findings into a compound-specific limit.

**TABLE 1:** Summary of main results from the end-to-end and modular workflow for the oral PDE derivation of acetyl tributyl citrate (CAS 77-90-7). The end-to-end results are generated using prompts of varying complexity that guide the full PDE derivation process. In contrast, the modular workflow results originate from multi-step prompts designed to support specific tasks of the TRA process. Additional details are included in the Supplementary Material.

Name: Acetyl Tributyl Citrate (Tri-n-butyl acetyl citrate) Short name: ATBC CAS RN: 77-90-7 SMILES: CCCCOC(=O)CC(CC(=O)OCCCC)(OC(C)=O)C(=O)OCCCC		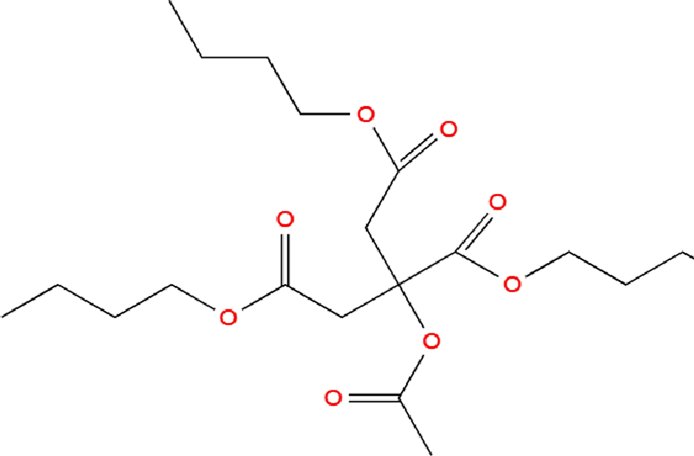
END-TO-END APPROACH		
Strategy	Approach	Considerations
Minimal end-to-end prompt (zero-shot prompt)	End-to-end ChatGPT minimal request was: “*Act as a toxicologist and derive the oral Permitted Daily Exposure (PDE) for acetyl tributyl citrate with CAS 77-90-7. Provide the details of the choice of the point of departure*”. Section S2.1 of the Supplementary Material provides further details of the tests.	Across multiple runs, several references were cited consistently, although the relative emphasis placed on individual sources varied. Some relevant evidence identified through manual data collection did not appear consistently in the ChatGPT-generated outputs. In addition, a subset of cited sources required verification of origin and authorship. Different NOAELs (e.g., 100 and 300 mg/kg bw/day) were identified as PoDs across the tests. Variability in the interpretation of these studies resulted in different oral PDE values, including 5, 20, 100, and 300 mg/day.
Structured end-to-end prompts: step-guided prompting (integrated evidence search)	Highly structured prompts were used to guide the model through the TRA process while producing a single, integrated PDE derivation. Evidence identification and retrieval were embedded directly within the end-to-end prompt, requiring the model to identify and select relevant sources during the analysis. Different operational modes (e.g., Auto run, Deep Research) were tested. See Section S2.2 of the Supplementary Material for further details of these tests.	Repeated runs using fixed detailed prompt wording showed variability in PoD selection, modifying factor application, cited evidence, and resulting PDE values. PoDs most commonly selected were 100 and 300 mg/kg bw/day, reflecting different interpretations and prioritization of available toxicity studies and regulatory documents, with corresponding oral PDEs spanning approximately 20–300 mg/day. The specific studies and regulatory documents cited to support PoD selection also varied across runs.
Structured end-to-end prompts: step-guided prompting (document-constrained configuration)	Highly structured prompts were used to guide the model through the TRA process while producing a single, integrated PDE derivation. Explicit data search functionality was excluded from the end-to-end prompt, constraining the model to rely solely on the uploaded documents as a fixed knowledge base, which included the draft ICH Q3E guidance to support the PDE derivation framework. See Section S2.3 of the Supplementary Material for further details of these tests.	Across multiple runs using a document-constrained configuration, variability was observed in PoD selection (100 or 300 mg/kg bw/day), modifying factor application, cited evidence, and resulting PDE values (approximately 14, 25, and 100 mg/day under different interpretations). The specific documents used to justify PoD selection also varied between runs, and the granularity of extracted evidence was inconsistent. When a highly detailed prompt was applied, the model’s stepwise reasoning became apparent, enabling identification of key decision points where evidence was overlooked, weighted differently, or interpreted divergently, thereby driving run-to-run differences.

MODULAR WORKFLOW		
Modules	Approach	Considerations
Data search	Targeted prompts for identifying relevant documents (e.g., reports, reviews, peer-reviewed publications) were best complemented by traditional data searches to ensure comprehensive coverage of evidence. See Section S3 of the Supplementary Material for prompt examples.	A set of documents forming an *ad hoc* knowledge base is compiled (see Section S6 of the Supplementary Material). The document corpus includes multiple complex documents, such as risk assessment reports from different organizations or peer-reviewed publications on ADME properties and toxicity studies.
Information extraction	Targeted prompts were used to identify and extract relevant evidence in line with the ICH Q3 draft guidance (ICH Q3E, 2025). See Section S3 of the Supplementary Material for prompt examples. This module produces an extracted dataset intended for expert verification.	In accordance with the ICH Q3 draft guidance, evidence is extracted from the dedicated knowledge base collected during the data search module. See Section S6 of the Supplementary Material for examples of extracted evidence.
Integrated analysis and summarization	Targeted prompts were used to summarize the extracted evidence in accordance with the ICH Q3 draft guidance. See Section S3 of the Supplementary Material for prompt examples. This module produces a summarized and interpreted evidence profile (e.g., genotoxicity, carcinogenicity, specific concern for endocrine disruption effects) for expert review against the underlying data.	The extracted evidence is summarized and structured according to the ICH Q3E draft guidance using data extracted from the knowledge base in the previous module. Examples of summarized evidence are provided in Section S6 of the Supplementary Material and are organized as follows: • Pharmacological/Biological Data • Toxicokinetics • Genotoxicity • Sensitization/Local Irritation • Acute Toxicity • Systemic Toxicity • Developmental and Reproductive Toxicity • Carcinogenicity • Additional Information
Expert interpretation	The expert may then select the appropriate PoD and modifying factors for the specific application, supported by ChatGPT through targeted prompts for analytical review. See Section S4 of the Supplementary Material for an overview of available evidence.	The LLM-summarized evidence suggests that a PoD of 100 mg/kg bw/day may be considered; however, the selection of an appropriate PoD and the application of modifying factors cannot be fully delegated to the model. These inherently non-deterministic decisions require expert judgment based on evaluation of uncertainty, weighting of the evidence, and protection goals.

GPT-5 (OpenAI), accessed via ChatGPT, was selected as the representative LLM for this case study. Initial analyses were conducted using GPT-5 and subsequently repeated using GPT-5.2 following its release (OpenAI, 2025c; OpenAI, 2025a; Singh et al., 2026). Importantly, overall behavioral patterns and conclusions were consistent across model versions. ChatGPT was chosen because it is a widely accessible LLM application that offers a conversational user interface without additional programming. In contrast, API-based, domain-driven, or agentic implementations require dedicated software development and workflow design. This work used model versions available between September and November 2025 (GPT-5, including subscription models) and between December 2025 and January 2026 (GPT-5.2, including Plus subscription models). It is noted that ChatGPT-exposed models are variants of the GPT-5/GPT-5.2 family and typically underperform relative to the corresponding OpenAI API models in independent evaluations. Benchmark results are documented through archived Artificial Analysis snapshots included in Supplementary Section S1 (retrieved November 2025) (Artificial Analysis, 2025a; Artificial Analysis, 2025b; Artificial Analysis, 2025c; Artificial Analysis, 2025d).

#### 3.1.2 LLM prompts

A progressive evaluation strategy was used to examine how increasing prompt structure, task decomposition, and constraints influenced LLM-assisted PDE derivation, including a document-constrained configuration in which the model’s reasoning was limited to user-provided documents. All tests were repeated across multiple independent runs, including sessions initiated by different users and sessions launched in incognito-mode. Additional tests explored different operational models, including Auto and Deep Research modes of ChatGPT. Three prompt strategies were implemented.

1. **Minimal end-to-end prompt (Zero-shot prompt).** A single, minimally constrained prompt was used. This prompt was kept intentionally broad and non-specific, requiring the model to independently retrieve evidence, summarize data, evaluate study quality, identify the PoD, apply modifying factors, and justify the resulting PDE in one integrated output. The prompt used was: *“Act as a toxicologist and derive the oral Permitted Daily Exposure (PDE) for Acetyl Tributyl Citrate (CAS 77-90-7). Provide details of the choice of the point of departure”.*
2. **Structured end-to-end prompts (Step-guided prompting).** More complex, highly structured prompts were used to guide the model through the TRA process, while still requiring the generation of a single, integrated PDE derivation as the final output. The prompts explicitly specified the expected assessment steps, decision criteria, and reporting format, thereby reducing ambiguity while preserving an end-to-end workflow within a single query. These prompts are included in the Supplementary Section S2.
3. **Task-specific prompt decomposition (Modular workflow)**. Prompts were designed for individual components of the TRA process decomposed into discrete workflow modules, namely:
  - data search
  - information extraction
  - integrated analysis and summarization
  - data interpretation and expert review

In this ChatGPT workflow, a manual, human-controlled approach was applied. Outputs were generated for each module, typically in tabular format, transferred manually into an external file, and then used as inputs for subsequent modular prompts. Exemplar prompts are included in the Supplementary Section S3.

In addition, a document-constrained approach was evaluated, in which LLM was constrained to operate exclusively on user-provided documents within both the step-guided prompting and the modular workflow. Unlike a true RAG system, this approach did not use automatic retrieval and dynamic indexing of documents. Instead, relevant documents were first identified and organized through a separate, manual data search stage, after which LLM-based reasoning was performed using only the material uploaded in the context window.

#### 3.1.3 Exploratory evaluation across LLM platforms

Although GPT-based models were used as the primary platform for the study, selected components of the case study were also explored using other widely used LLM platforms, including Claude Sonnet 4.5 and Gemini 3 (representative outputs are provided in the Supplementary Material). Minor prompt adaptations were introduced where necessary to accommodate model-specific best practices. These exploratory evaluations were not intended as a formal benchmarking study; rather, they were used to assess whether the general observations arising from the case study were broadly consistent across different LLM platforms. Overall, the observations were qualitatively similar and did not materially alter the overall analysis. It is noteworthy that different models (e.g., ChatGPT, Claude) require model-specific prompt engineering and that the feasibility of certain workflows, including document-constrained approaches with large document sets, may be limited by platform-specific features or license-dependent limitations.

#### 3.1.4 PDE derivation

The overall safety assessment requires integration of diverse toxicological evidence including: pharmacological or biological activity, toxicokinetics, systemic toxicity, sensitization and local irritation, developmental and reproductive toxicity (including endocrine disruption), genotoxicity and carcinogenicity, and additional evidence such as clinical data, regulatory classifications, or related compound information (ICH Q3E, 2025). PDE derivation herein is discussed within the context of the draft ICH Q3E guideline for Extractables and Leachables (ICH Q3E, 2025). The selected test substance, acetyl tributyl citrate (ATBC), represents a data-rich chemical, with numerous toxicological studies and existing regulatory assessments that must be integrated to form a coherent weight of evidence evaluation. A new PDE is derived by first identifying a critical nonclinical repeat-dose toxicity study documenting a well-supported no-observed-adverse-effect level (NOAEL) or lowest-observed-adverse-effect level (LOAEL), followed by the application of appropriate modifying factors to derive a daily dose over a lifetime considered as safe for humans.

### 3.2 Observations from the case study

#### 3.2.1 Summary of case-study results

The analysis of the safety assessment of ATBC draws on relevant literature, the authors’ prior experience, and results from the LLM-assisted case study of PDE derivation (Table 1) to generate observations regarding the strengths and limitations of LLMs, using ChatGPT as a representative model. A series of controlled experiments was conducted using ChatGPT in different operational modes, with additional details of the generated outputs provided in the Supplementary Section S2. The results of the PDE derivation for ATBC are summarized in Table 1. Further details of the principal observations from the case study, including source selection, evidence coverage, reasoning, hallucinations, prompt design, data extraction, summarization, and PDE variability, together with supporting literature, are provided in the Supplementary Material. Exploratory investigations were also conducted with other models (e.g., Claude) and are summarized in the Supplementary Material as well.

Overall, it was observed that LLMs supported rapid data screening, extraction, and summarization, but several well-recognized limitations were noted when used with end-to-end prompts. In particular, outputs varied across repeated interactions. Other observed issues included incomplete data coverage, occasional fabricated or unverifiable citations, limited transparency of reasoning, and variability in toxicological interpretation.

Across independent runs using end-to-end prompts (i.e., prompts directing the LLM to complete the full TRA process, namely, zero-shot prompt and step-guided prompting), the LLM-assisted analysis did not consistently select the same PoD for ATBC; instead, values of 100 and 300 mg/kg bw/day were most frequently identified. Consistent with this variability, a range of oral PDE values (e.g., 5, 20, 100, and 300 mg/day depending on iterations and prompt design) were based on different interpretations and modifying factors, as summarized in Table 1. Notably, a PoD of 100 mg/kg bw/day was previously selected by EFSA for the derivation of a tolerable daily intake (TDI) of 1.0 mg/kg bw/day based on mild effects on body weight and some biochemical parameters from 90-day oral toxicity studies in rats, also including an *in utero* exposure phase, and in a two-generation reproduction study using rats (EFSA, 2005). However, available TRA reports differ in their interpretation of the NOAEL from key studies, particularly regarding whether certain effects are considered adaptive or adverse. While a conservative assessment identifies 100 mg/kg bw/day as the NOAEL, other evaluations consider 300 mg/kg bw/day as the PoD (Anses, 2016; CPSC, 2018; CIR, 2019). Endocrine activity represents an additional area of ongoing discussion in the literature for ATBC, further contributing to divergent interpretations of evidence (Anses, 2016; Rasmussen et al., 2017).

In addition to end-to-end prompting, exploratory tests were conducted with a modular approach using task-specific prompt decomposition, in which the components of the TRA process were addressed as separate prompts (see Table 1). Structuring the LLM-powered workflow into sequential modules made intermediate outputs explicit and reviewable while leveraging the LLM’s strengths.

Overall, these findings suggest that LLMs can support TRA decision-making when their methodological and technical limitations are appropriately recognized and addressed. The following sections first describe the challenges and limitations identified in this case study, then present corresponding mitigation strategies (Table 2), and finally discuss the observed strengths of LLMs in the context of TRA. The proposed mitigation strategies are based on two core principles: (i) human-in-the-loop control, whereby subject-matter experts retain ownership and accountability for decisions underlying PDE derivation, and (ii) a structured workflow that organizes the TRA process across distinct modules, i.e., data search, data extraction, evidence summarization, and conclusion drawing. Together, these principles are intended to support the effective and responsible use of LLMs in TRA.

**TABLE 2:** Limitations observed when applying LLMs (ChatGPT, GPT-5 family models) to the derivation of PDEs, and corresponding mitigations.

Limitation	Description	Mitigations
Inconsistent outputs	Repeated queries often produce varying results, including differences in applied factors, selected critical studies, and cited sources	A structured retrieval protocol can anchor responses to the same verified evidence set, while controlled prompts and a stepwise expert review improve consistent interpretation and reproducibility. RAG approaches coupled with expert review can reduce source-related variability and improve traceability in scientific LLM applications (Abo El-Enen et al., 2025; Gan et al., 2025; Ke et al., 2025; Zhang et al., 2025a). Even with the same knowledge base, variability may persist because TRA involves the interpretation of evidence, and alternative scientifically defensible conclusions may be reached. Subject-matter expert review therefore remains essential.
Incomplete coverage	LLM responses do not reliably capture the full body of relevant evidence	A structured retrieval protocol should be applied to build a comprehensive and validated knowledge base that ensures document-level completeness. This process should verify that all relevant toxicology reports are identified, included, and available as the foundation for subsequent analysis and summarization tasks. Incorporating targeted manual review and traditional search methods may help further improve retrieval accuracy and completeness. RAG and prompt engineering constitute distinct LLM optimization strategies that can improve the output coverage.
Superficial reasoning	Contextual analysis may lack depth (e.g., misinterpretation of PoD, oversimplification of bioavailability considerations)	The LLM’s role should be controlled within a structured workflow that governs evidence extraction, summarization, and interpretation. The model can be prompted to provide preliminary interpretations or alternative viewpoints, but always through controlled prompts that guide how evidence is analyzed and discussed. Expert toxicologists retain responsibility for interpretation and PoD selection. Incorporating controlled prompting and human-in-the-loop review into the workflow improves the output quality (e.g., Bednarczyk et al., 2025) and aligns the workflow with best practices reported in recent literature.
Hallucinations/Confabulations	Outputs may include fabricated references, inaccurate quotations, or unsupported statements presented as factual	To minimize hallucinations, all model outputs should be explicitly linked to verifiable sources. Each claim or data point must be supported by a corresponding citation to the primary document, with unverifiable items flagged for verification and excluded unless confirmed. This evidence-attribution requirement reflects a key safeguard underlying RAG approaches, in which model outputs are constrained to retrieved source material to reduce unsupported generation (Lewis et al., 2021; Zhang and Zhang, 2025). Expert toxicologists should cross-check these citations to confirm accuracy and context before acceptance.
Multi-document extraction instability	Performance may degrade when multiple large documents exceed context window limits, leading to truncated or missed information	Multi-document extraction should follow an extraction workflow, in which each report is processed individually prior to summarization, using models with sufficient context window capacity to accommodate document length; extracted outputs are then combined in a subsequent summarization stage to avoid truncation and incomplete evidence use.
Traceability gap	Outputs may vary across model deployments and tool configurations and may reflect a static or unknown knowledge cutoff	All LLM-generated outputs should be logged with tool, model, version, date/time, and knowledge cutoff (or “unknown”) to ensure accountability and allow for audit tracking of changes due to model updates or cutoff shifts.

#### 3.2.2 Case study-specific challenges

The following observations describe how the technical characteristics discussed in Section 2.1 manifested in practice during the LLM-assisted PDE derivation case study. Details on these observations and their interpretation in the context of the available literature are further provided in the Supplementary Material.

##### 3.2.2.1 Inconsistent outputs

Responses to end-to-end prompts requesting derivation of a PDE for ATBC showed variability both in the sources cited to support the derivation and in resulting outcomes (PoD and PDE values).

The variability observed in LLM outputs regarding the PoD selection and corresponding PDE values may reflect both inherent model limitations and the intrinsic complexity of TRA, in which different experts can reasonably interpret the same evidence to arrive at different, yet scientifically defensible, conclusions. Beyond sensitivity to prompt design and task specification, LLMs are stochastic systems and therefore do not guarantee identical outputs across repeated runs, even when using identical prompts. This variability is expected to be more pronounced for tasks requiring complex expert judgment, such as interpreting toxicological evidence, selecting a critical study, or applying modifying factors. Importantly, PDE derivation itself is not a fully deterministic exercise, even when performed by human experts. Alternative, scientifically justified assumptions regarding study relevance, endpoint adversity, and exposure duration can legitimately yield different PDE values. Accordingly, a portion of the variability observed in LLM-assisted outputs may arise from the inherent non-uniqueness of the TRA process rather than from model limitations alone.

##### 3.2.2.2 Incomplete coverage

The data sources cited in the ChatGPT responses within the case study were often incomplete when compared with those identified via expert evaluation, creating a risk of omitting relevant data or failing to critically evaluate certain evidence. Reviews of LLM use in healthcare discuss such types of gaps and uneven coverage (Fatima et al., 2024; Li J. et al., 2024; Parisi and Sutton, 2024). Because PDE derivation depends on the totality of evidence (including negative studies, route-specific data, and the most recent regulatory guidance), reliance on an LLM without a structured retrieval workflow poses a risk of omitting critical information. In API-based implementations, evidence coverage can be better controlled through retrieval pipelines (e.g., document chunking, embeddings-based search, and controlled source selection), which may reduce such omission (e.g., Gao et al., 2023; Fan et al., 2024; Oche et al., 2025).

##### 3.2.2.3 Reasoning limitations

Reasoning and contextual analysis in the ChatGPT responses within the case study could lack sufficient depth. This limitation was evident, for example, in the selection of the PoD, the choice of modifying factors, or interpretation of effect severity, where key considerations may be misinterpreted or oversimplified. In some instances, the model appeared to paraphrase or restate the conclusions of a single source document without integrating evidence across multiple studies and documents, potentially overlooking conflicting findings (e.g., Ge et al., 2025) or broader contextual information. Limitations in context handling were observed including under-using information placed in the middle of long reports. Certain configurations of the case study like the Deep Research agentic mode of ChatGPT (OpenAI, 2025b; Xu and Peng, 2025) and step-guided prompting generated more detailed responses. However, such responses were not consistently grounded in all the relevant knowledge base and occasionally incorporated sources of uncertain relevance particularly in non-document-constrained approaches. Overall, accessing the same models via the API is not expected to resolve these reasoning limitations, although multi-step, tool-augmented pipelines (e.g., retrieval-augmented or agentic/orchestration workflows) are more likely to enforce structured reasoning and systematic evidence integration.

##### 3.2.2.4 Hallucinations/confabulations

Consistent with other LLM research, the evaluation of the PDE generated by the model also revealed isolated instances of hallucinations, although their occurrence was not quantified. Such confabulations could be difficult to detect without careful verification. For example, the LLM output included statements with quotations or citations that, upon closer inspection, proved to be inaccurate or fabricated in line with available literature (Topaz et al., 2026; Zhao et al., 2026).

##### 3.2.2.5 Multi-document extraction instability

When multiple large toxicology reports were uploaded at once, ChatGPT (including newer versions) sometimes showed reduced reliability, including missed or mixed evidence and incomplete outputs. This behavior is primarily associated with interface-related limitations that may occur with very large or numerous PDFs. API-based workflows remain better suited for comprehensive analysis of large toxicology datasets. Examples of interface limitations include the context window, the size and number of the uploaded files, and parsing and vision barriers.

#### 3.2.3 Mitigation strategy

Identified limitations from the case study can be mitigated through a combination of measures, including decomposing complex tasks into smaller, well-defined actions and the application of structured retrieval protocols to build a verified knowledge base (Table 2). A key methodological outcome of this work is that data retrieval should be treated as a distinct task from data extraction and summarization during prompt formulation, particularly when dealing with large and complex evidence bases such as those associated with the tested chemical. This separation helps maintain the LLM's focus on the most salient points, which in turn improves completeness, enhances reproducibility, and facilitates expert review. Additional controls, such as guided prompting, explicit source citation, human-in-the-loop review, and document-by-document processing prior to summarization, can further improve reliability and traceability (Table 2), favoring focused, well-scoped tasks supported by detailed and structured prompts.

#### 3.2.4 Observed strengths of LLM-assisted tasks

The PDE case study illustrates several strengths of LLM-assisted approaches that can be leveraged using a structured workflow guided by the mitigation strategies outlined in Table 2. In particular, the observations suggest that LLMs can support specific tasks of TRA when applied to well-defined subtasks, using carefully designed prompts, and integrated within an expert review process.

One of the most prominent strengths observed is time efficiency. Tasks that traditionally require substantial manual effort (e.g., extracting relevant information from full-text PDFs, generating structured summaries, and identifying or filling specific data gaps within the evidence framework used for PDE derivation) can be completed more rapidly with LLM support. It may also be noted that the ability of LLMs to scan and summarize large and heterogeneous data sources can help uncover relevant details that may otherwise be overlooked during manual review, particularly under time or resource constraints.

In this case study, it is also observed that LLMs support efficient summarization of toxicological information, especially when task decomposition and structured prompt engineering are applied. Rather than relying on unconstrained, end-to-end prompts, decomposing the TRA process into discrete modules can yield intermediate outputs that can be reviewed and validated by experts. This observation is in line with the mitigation strategies proposed in Table 2, which suggests structured workflows to reduce superficial reasoning and improve reproducibility.

The case study further illustrates how the nature and specificity of prompts can affect LLM outputs, highlighting the influence of prompt design on responses. For example, focused queries aimed at identifying available evidence for a specific hazard (e.g., endocrine disruption potential) produced useful responses that could be reviewed and interpreted by experts. In contrast, complex, multi-step tasks requiring implicit toxicological judgment (such as deriving a complete PDE within a single prompt) often resulted in more variable outputs. These observations motivate the strategy proposed in Table 2, whereby LLMs are incorporated into stepwise workflows under expert review rather than as autonomous decision-makers.

In the present case study, LLM-assisted searches contributed to literature triage and evidence discovery, including revealing potentially overlooked studies when guided by expert-defined questions. In general, this capability can reduce manual iteration cycles in the data search process.

Finally, when provided with relevant source documents and suitable prompts, LLMs could extract key study attributes into structured tables, including study identifiers, exposure details (route and duration), test system characteristics (species and sex), measured endpoints, effect levels (NOAEL/LOAEL values), and direct citations. This structured output can support quality checking by subject-matter experts through consistent organization of extracted information. The associated citation system that could be enforced through appropriate prompting, would create a clear audit trail, enabling verification against original sources and supporting the core principles of explainability, transparency, and accountability.

In summary, the analysis of the case study suggests that the primary value of LLMs in this context does not lie in deriving a single definitive PDE value, but rather in their ability to systematically summarize the available body of evidence, surface alternative interpretations, and explicitly articulate assumptions and judgment points that influence different outcomes. These observations support the use of LLMs as components of structured, expert-supervised workflows for TRA.

## 4 Discussion: a pragmatic workflow for LLM-assisted TRA

The overall rationale underlying the proposed workflow is summarized in Figure 1. Deployment of LLMs for TRA requires frameworks for error detection, risk grading, and human-in-the-loop validation (Asgari et al., 2025; Lui, 2024). A pragmatic approach to meeting these requirements is to embed LLMs within a modular, supervised workflow that decomposes the overall procedure for safety assessment into discrete, reviewable stages, including evidence collection, extraction, appraisal, and integration. Each module serves as a checkpoint for expert evaluation and approval, ensuring transparent and accountable human-in-the-loop integration.

Recent studies indicate that decomposing complex workflows into sequential components can enhance LLM reasoning performance and operational reliability (Huang et al., 2024). Similarly, insights from human–LLM collaboration systems highlight the value of embedded human feedback and orchestration mechanisms for guiding LLMs through multi-stage workflows and improving reliability (Zou et al., 2025). Modular approaches (including reflection mechanisms, reasoning trees, and structured prompting) represent key strategies for mitigating common limitations such as reasoning failure, hallucination, and limited controllability (Kostikova et al., 2025).

Observations from the PDE case study further illustrate these principles. Observed strengths, limitations, and mitigation strategies from the case study emphasize the need to deploy LLMs within a supervised, expert-led framework in which LLM functions as a supportive tool for well-defined subtasks, while subject-matter experts validate outputs, refine interpretations, and retain accountability at each stage. The proposed workflow, shown in Figure 2 and informed by the PDE derivation, provides a structured framework for integrating LLM-assisted tasks into TRA under expert supervision through four main, sequential modules: (1) data search, (2) information extraction, (3) integrated analysis and summarization, and (4) expert interpretation. At every stage, LLM-assisted tasks can be reviewed and monitored by experts for relevance, accuracy, and traceability; expert interpretation and decision-making are consolidated in the final module.

**FIGURE 2:**
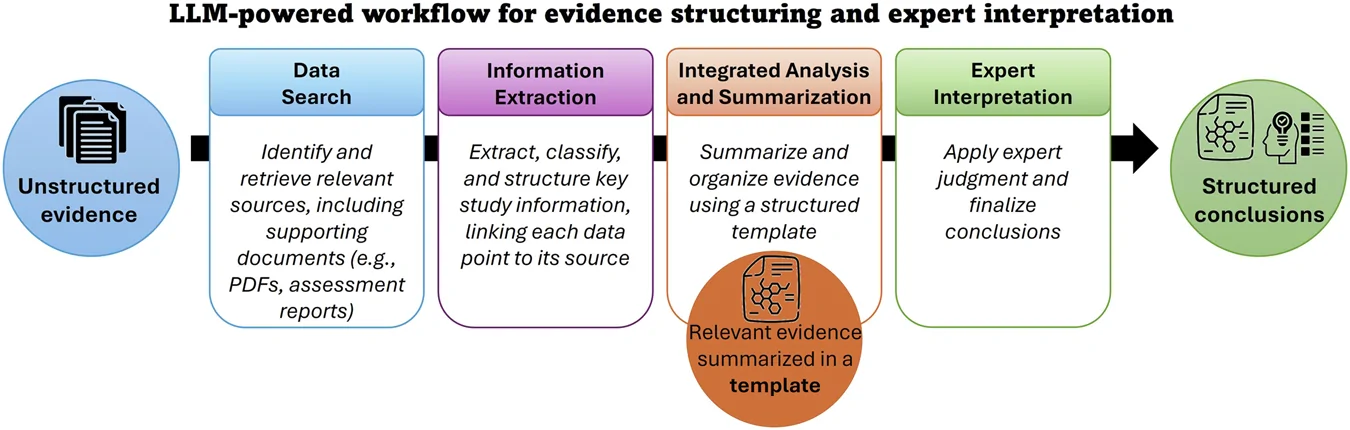
Proposed LLM-powered workflow supporting TRA. The workflow is intended to support the integration of LLMs into TRA and transform unstructured evidence (e.g., documents, tables, text) into expert-driven conclusions through four sequential modules. (1) Data search identifies and retrieves relevant evidence sources. 2) Information extraction converts relevant content into structured summaries or tabular outputs. 3) Integrated analysis and summarization combine information across sources and organize the evidence according to the assessment objective and reporting requirements. 4) Data interpretation and expert review evaluate the summarized evidence, apply toxicological judgment, and support the derivation of assessment conclusions. Representative prompts are provided in the Supplementary Material to illustrate how the workflow modules may be implemented in practice.

### 4.1 Workflow module: data search

Data search identifies and retrieves potentially relevant evidence sources. The retrieved toxicological information forms a knowledge base that augments the standard LLM’s training knowledge. This augmented LLM approach enables models to operate on verified, domain- and chemical-specific evidence. This knowledge base provides the foundation for all subsequent modules, helping to maintain traceability to original sources and support verification. In this module, LLM-assisted retrieval should be facilitated by appropriate prompts and complemented by expert-guided manual identification of critical resources to support the completeness, relevance, and scientific quality of the sources.

### 4.2 Workflow module: information extraction

Once the knowledge base is established, the next module focuses on extracting, classifying, and structuring the relevant evidence needed to inform the specific TRA. In this stage, LLMs assist in identifying and organizing data (e.g., study design, exposure route, endpoints, species, results, and the authors’ conclusions) from the collected documents. The output of this module is a structured dataset that links each extracted data element to its source, creating a transparent and auditable foundation for subsequent summarization and interpretation.

Extraction prompts can be designed to promote consistency in data capture across documents and studies. Subject-matter expert review remains important for targeted verification of critical extracted information, particularly key toxicological endpoints, study limitations, and contextual factors that may influence downstream interpretation.

### 4.3 Workflow module: integrated analysis and summarization

Integrated analysis and summarization combine extracted information across sources and organize the evidence according to the main objective of the assessment. Evidence extracted from the knowledge base is summarized to develop a TRA report, including, for example, the substance’s toxicological profile and mode of actions. Throughout this stage, the structured dataset remains subject to expert review and curation, allowing removal or correction of entries deemed incomplete, inaccurate, or of insufficient methodological quality.

LLM prompts in this module may be used to organize extracted data, facilitate analysis, compare findings across studies, identify recurring toxicological effects, summarize evidence by endpoint, mode of action, or study type, and highlight areas of agreement, uncertainty, or conflicting evidence. The resulting output provides a harmonized overview of the available evidence that supports data interpretation, mechanistic understanding, and expert judgment, including the identification of critical and severe effects.

### 4.4 Workflow module: expert interpretation

In the final module, the structured and reviewed evidence is interpreted through expert reasoning that integrates professional judgment and relevant contextual information. In the context of PDE derivation, this includes selection of the most appropriate PoD study and critical effects, followed by application of modifying factors. Prompts may, where possible, capture the relevant decision framework and underlying reasoning rules (e.g., modifying-factor assignment) of the assessment to improve consistency, transparency, and reproducibility. Notably, LLMs may support the reasoning process by providing not only a comprehensive overview of the collated evidence but also a complementary analytical perspective. Prompts in this module may also support reasoning by evaluating alternative interpretations of the evidence, identifying key uncertainties, and highlighting patterns or relationships across the evidence base that may warrant further expert consideration. However, final determinations rest exclusively with human toxicologists, who evaluate the overall weight of evidence to reach scientifically justified conclusions.

## 5 Conclusion

### 5.1 Fit-for-purpose applications of LLMs

As summarized in Figure 1, observations arising from the case study and their interpretation in the context of the available literature informed the development of the workflow hypothesis. The resulting workflow (Figure 2) reflects the observation that different TRA tasks place different demands on LLMs and proposes that the suitability of LLM-assisted TRA depends on how LLMs are integrated into the assessment process (Figure 3).

**FIGURE 3:**
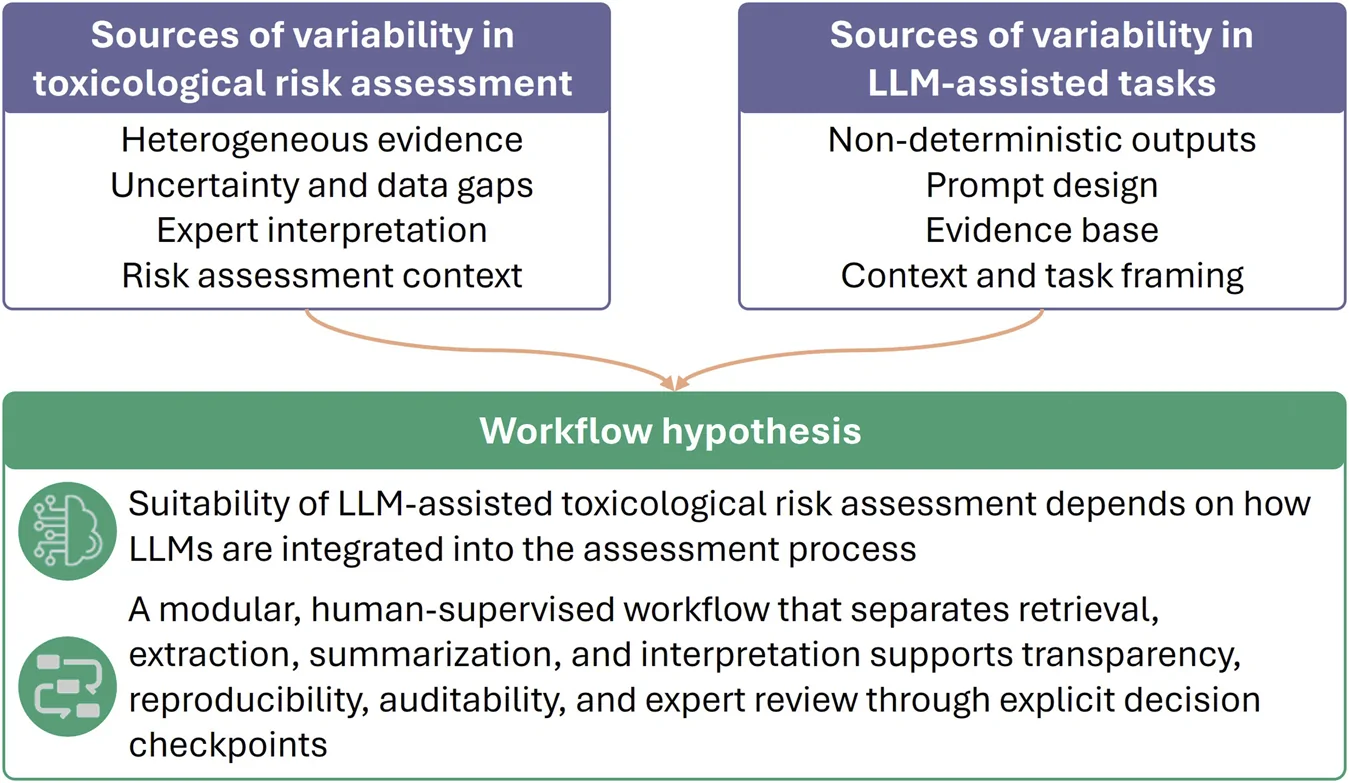
Conceptual hypothesis underlying LLM-assisted TRA. Variability in TRA arises from characteristics of the assessment domain, including heterogeneous evidence, uncertainty, and the possibility of different expert interpretations of evidence. Additional variability arises from characteristics of LLM-assisted tasks, including non-deterministic outputs, prompt design, and differences in the available evidence base. Based on these observations, the workflow hypothesis proposes that the suitability of LLM-assisted TRA depends on how LLMs are integrated into the assessment process. Specifically, the hypothesis supports a modular, human-supervised workflow that separates evidence retrieval, information extraction, evidence summarization, and expert interpretation, with explicit decision checkpoints.

Current literature and observations from the present case study suggest that LLMs can be particularly useful for tasks involving pattern recognition, information retrieval, and summarization. LLM-assisted extraction has shown promising performance for structured data extraction and evidence synthesis tasks, although rigorous comparisons with human extractors remain limited (Sonnenburg et al., 2024; Schmidt et al., 2025; Wang et al., 2025). The present case study extends this discussion beyond extraction reflecting on how extracted evidence is combined and interpreted to derive a compound-specific limit. In line with available literature, challenges arise when tasks require multi-step reasoning across multiple documents, particularly when evidence must be weighted, reconciled, or sequentially integrated into processes that demand domain expertise and expert judgment. Within this context, one potential application of LLMs in TRA is the systematic and transparent summarization of the underlying body of evidence, providing a structured basis for expert review, interpretation, and conclusion drawing.

These capabilities can be further strengthened by pragmatically implementing workflow-based approaches that introduce explicit checkpoint steps for expert review. In data-rich contexts, separating data retrieval from extraction and summarization is essential to preserve transparency, ensure completeness, and support expert review. Conversely, in data-poor scenarios, where only a limited number of documents are available, data search and extraction may be combined into a single end-to-end LLM prompt. Even in such cases, however, verification of available evidence through a dedicated retrieval workflow remains important to support comprehensive evidence collection.

As LLM technology continues to evolve, these models may increasingly be used as first-pass triage tools through end-to-end prompts, independently of expert review. In this role, they can support preliminary mapping of the evidence landscape, highlight potentially relevant data sources, identify information gaps, and guide experts toward areas requiring deeper evaluation.

### 5.2 Human-in-the-loop

When coupled with structured retrieval and extraction protocols, as summarized in Table 2, LLMs may reduce the effort associated with manual compilation and allow human resources to be redirected toward higher-level tasks such as judgment and interpretation (e.g., selection of PoD and modifying factors). This potential redistribution of effort is consistent with the view that human–LLM collaboration in systematic reviews may increase the speed and depth of evidence summarization (Hauser, 2025). As part of a human-in-the-loop configuration, LLMs may thus be particularly useful for systematic extraction and formatting tasks, while expert toxicologists retain responsibility for interpretation, evidence weighting, and final decision-making.

The potential value of LLM-powered TRA workflows therefore depends not only on reductions in time required for data extraction and summarization, but also whether human expert effort can be shifted from manual data collection to evaluation and interpretation. However, the modular workflow proposed in Figure 2 is currently expected to require expert review, source verification, and stepwise control, even when implemented within structured software platforms. As a result, efficiency gains may be partially offset by the effort required for review, verification, and correction of generated outputs. For subject-matter experts, adoption of LLM-assisted approaches is likely to depend on whether such workflows support a transition from summarization to interpretation, and whether this transition yields clearer data organization, improved traceability, and net time gains. Additionally, the proposed LLM-powered modular framework is intended to integrate expert judgment at key decision points where resolution requires human expert interpretation. This reflects the fact that variability in outcomes originates not only from the stochastic behavior of LLMs, but also from the inherent non-determinism and context dependence of TRA itself.

### 5.3 Interpreting variability in LLM-assisted TRA

An important observation from this work is that variability in LLM-assisted outputs should not necessarily be interpreted solely as evidence of model limitations. Although LLM behavior is affected by stochastic generation, prompt design, and task specification, variability may also reflect the expert-judgment-dependent nature of TRA. For example, PDE derivation does not always yield a single uniquely correct outcome, and different toxicologists may reach different conclusions based on the same evidence (e.g., Sehner et al., 2024), leading to different PoD selections and PDE values. This observation suggests that evaluation of LLM-assisted TRA should consider not only reproducibility and accuracy, but also the broader context of scientific uncertainty and expert judgment that characterizes the risk assessment process itself.

### 5.4 LLM integration in TRA

Transparency in LLM use and regulatory acceptance may be enhanced by the integration of curated evidence sources (e.g., through RAG) and by explicitly embedding core toxicological concepts, assessment steps, and decision constraints within LLM-powered workflows. While RAG can improve factual grounding by constraining outputs to retrieved sources, the present case study observed that document-constrained approaches still produced variable PoD selections and PDE values across runs. This finding suggests that grounding alone is insufficient for TRA, as the same evidence base may support multiple conclusions. Consequently, the workflow structure should not only provide access to relevant evidence but also make the reasoning pathways linking evidence to conclusions explicit and reviewable. By providing explicit evidence and controlling workflow sequencing, approaches such as structured orchestration or domain-driven design may thus reduce the need for the model to infer domain-specific reasoning and logic. This explicit formalization is intended to support conceptual consistency, traceability, and auditability, which are important considerations for regulatory acceptance in TRA. Although these approaches may require additional upfront technical development and system design effort, they can also offer opportunities to reduce downstream risks and improve the long-term reliability of LLM-assisted assessments.

### 5.5 Final remarks and perspectives

This work proposes that LLMs may support TRA, including the derivation of PDEs, when their use is embedded within structured, transparent, and expert-driven workflows. This workflow hypothesis is informed by observations from the case study and their interpretation in the context of the available literature. The proposed pragmatic workflow combines modular task decomposition, human expert review, and structured evidence handling to support the integration of LLMs into TRA. By assigning complementary roles to LLMs and subject-matter experts and incorporating mitigation strategies for known limitations and challenges, the framework aims to support transparent and robust assessments.

A key methodological insight is that data retrieval, information extraction, and evidence summarization may be treated as distinct stages within LLM-powered workflows, particularly in data-rich assessment contexts. This separation is intended to facilitate completeness checks, support reproducibility, and aid expert review. Although such structured workflows impose additional controls and burden, these safeguards are critical for regulatory applications, where transparency, accountability, and defensibility are fundamental requirements. Importantly, while structured workflows and traceable evidence handling can reduce risk and improve auditability, they do not replace the need for expert verification and documented decision-making in TRA.

In addition, observations from the case study suggest that variability in LLM-assisted outputs may reflect not only model limitations but also the inherently judgment-dependent nature of TRA, further reinforcing the need for transparent, human-supervised workflows. Accordingly, LLM assistance may be viewed as one component of a broader quality system in which LLM tools, expert judgment, and systematic review operate in concert to support defensible toxicological decisions.

A logical next step of this work is the application of the proposed pragmatic workflow to additional TRA-relevant case studies, spanning data-rich and data-poor substances as well as re-evaluations, to further examine its performance and limitations across different regulatory scenarios. Early engagement with regulatory bodies and international organizations in these evaluations could provide regulatory perspective and facilitate later acceptance and implementation of LLM-assisted TRA processes.

## Data Availability

The original contributions presented in the study are included in the article/Supplementary Material, further inquiries can be directed to the corresponding author.
