# Supplementary material for: A pragmatic workflow for integrating large language models into toxicological risk assessment
Source: Front Toxicol. 2026 Sep 28;8:1877128. doi: 10.3389/ftox.2026.1877128 (PMC13654619; doi:10.3389/ftox.2026.1877128)
Supplement: Supplementary file 1 [file DataSheet1.pdf]

Supplementary Material

The present file includes the prompts used in this investigation, primarily applied to ChatGPT. It also includes summarized evidence related to the test chemical examined for the LLM-assisted PDE derivation.

Contents

Section S1: LLM benchmarking ... 2
Section S2: End-to-end PDE derivation ... 3
S2.1. Minimal end-to-end prompt – Zero-shot prompt (with and without Deep Research) ... 5
S2.2. Structured end-to-end prompts – Step-guided prompting (with and without Deep Research) ... 5
Structured prompt (short) ... 6
Structured prompt (long) ... 7
S2.3. Use of a predefined knowledge base: document-constrained configuration (with and without Deep Research) ... 18
Document-constrained configuration prompt ... 18
Structured prompt adjusted for document-constrained configuration ... 18
Section S3: Task-specific prompt decomposition – Modular workflow ... 20
S3.1. Data search – Example of ChatGPT prompts ... 20
ChatGPT prompt for reviews ... 20
ChatGPT prompt for peer-reviewed publications ... 20
ChatGPT prompt to retrieve evidence supporting the safety assessment ... 20
S3.2 Information extraction – Example of ChatGPT prompts ... 20
Prompt for Pharmacological/Biological Data ... 20
Prompt for Toxicokinetics ... 21
Prompt for Genotoxicity ... 22
Prompt for Sensitization and irritation ... 24
Key toxicological data (systemic toxicity, DART, carcinogenicity) ... 26
S3.3. Integrated analysis and summarization – Example of ChatGPT prompts ... 30
Prompt for Pharmacological/Biological Data ... 30
Prompt for Toxicokinetics ... 31
Prompt for Genotoxicity ... 32
Prompt for Sensitization and Irritation ... 34
Prompt for Key toxicological data (systemic toxicity, DART, carcinogenicity) ... 35
Section S4: Acetyl Tributyl Citrate (CAS 77-90-7) – supporting evidence ... 37
S.4.1. Data search ... 37
S.4.2. Information extraction ... 39
S.4.3. Integrated analysis and summarization ... 41
Pharmacological/Biological Data – summarized evidence (example) ... 41
Toxicokinetics – summarized evidence (example) ... 41
Genotoxicity – summarized evidence (example) ... 42
Systemic Toxicity – summarized evidence (example) ... 42
Reproductive and developmental toxicity – summarized evidence (example) ... 43
S.4.4. Expert interpretation ... 44
AI-assisted point of departure identification – example ... 44

Section S1: LLM benchmarking

Benchmarking insights on large language models were examined using data from Artificial Analysis (https://artificialanalysis.ai/). Artificial Analysis is an independent benchmarking and insights organization that evaluates AI models (particularly LLMs) across a wide range of performance dimensions. Their comparisons include quality, cost, output speed, latency, context-window capacity, and other operational metrics. Because LLM capabilities evolve rapidly, model rankings and performance characteristics can change significantly over short periods.

GPT-5 models demonstrated good performance across several benchmarking tests such as the long-context reasoning benchmark (assessing an LLM's ability to retrieve, integrate, and summarize information across multiple long documents), the GPQA test (a benchmark of graduate-level physics, biology, and chemistry questions authored by domain experts, designed to evaluate scientific-reasoning capabilities) and the IFBench test (a benchmark assessing instruction fidelity and reporting discipline, testing whether an LLM can follow complex, multi-step, and compositional instruction.

Below, several performance graphs comparing leading models are reported, based on data retrieved from the Artificial Analysis website in November 2025 (https://artificialanalysis.ai/evaluations):

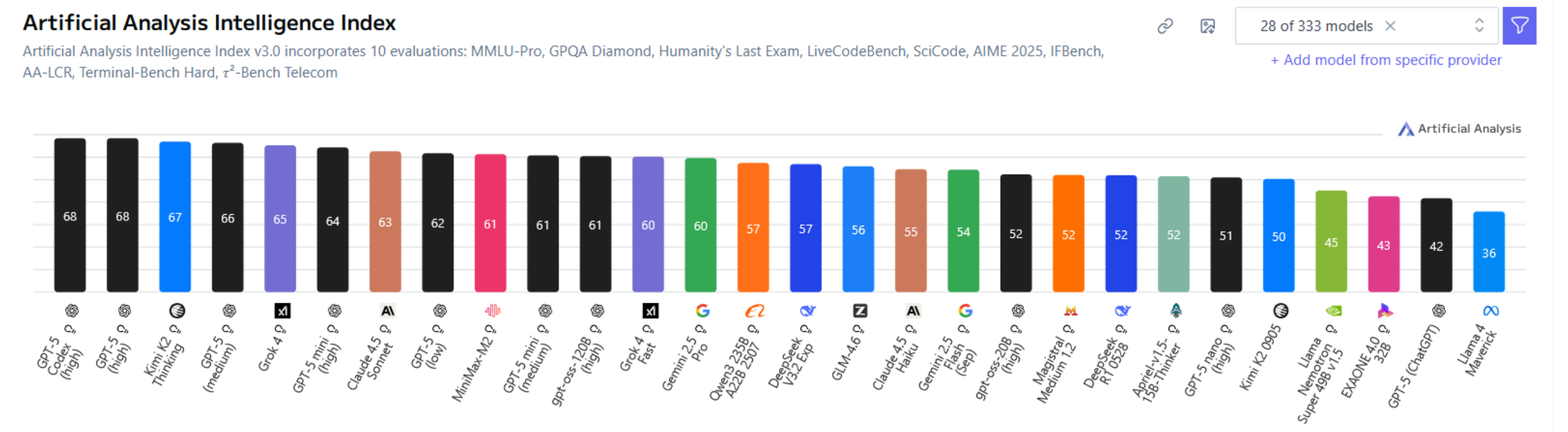

Artificial Analysis Intelligence Index v3.0 incorporates 10 evaluations: MMLU-Pro, GPQA Diamond, Humanity's Last Exam, LiveCodeBench, SciCode, AIME 2025, IFBench, AA-LCR, Terminal-Bench Hard, τ^2-Bench Telecom

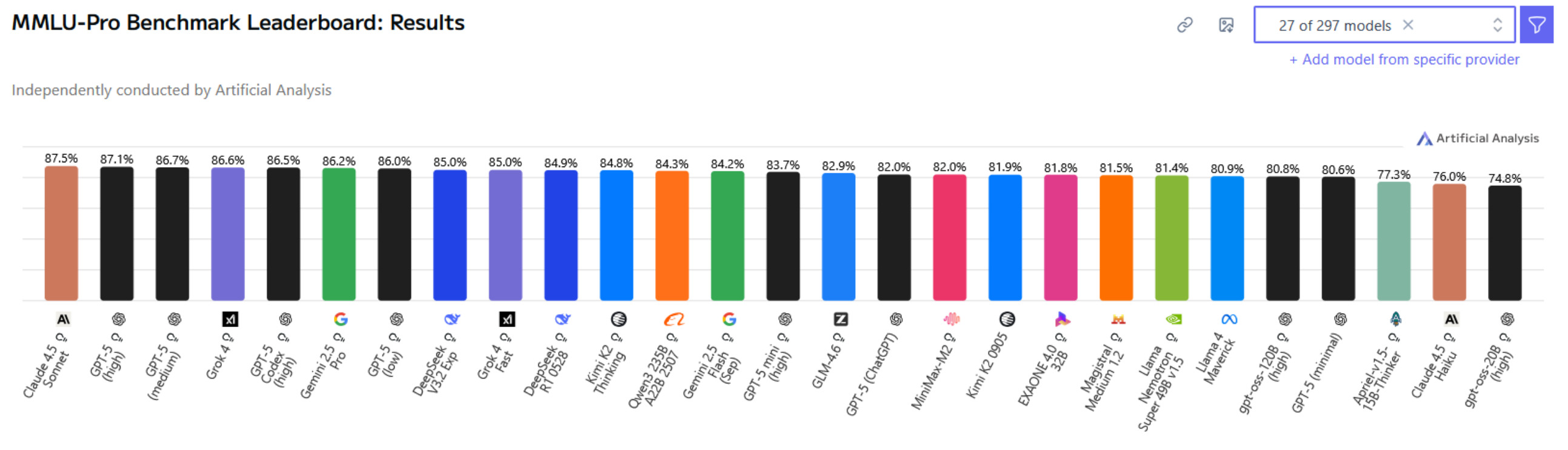

12,000 graduate-level questions across 14 subject areas, featuring ten answer options and deeper reasoning requirements.

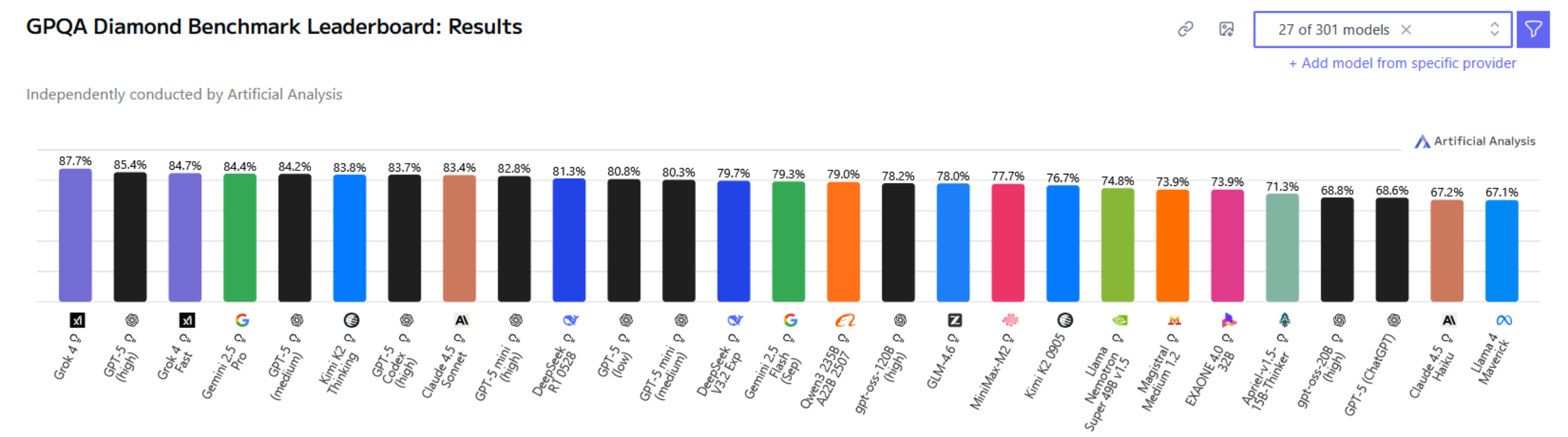

GPQA is a challenging dataset of 448 multiple-choice questions written by domain experts in biology, physics, and chemistry.

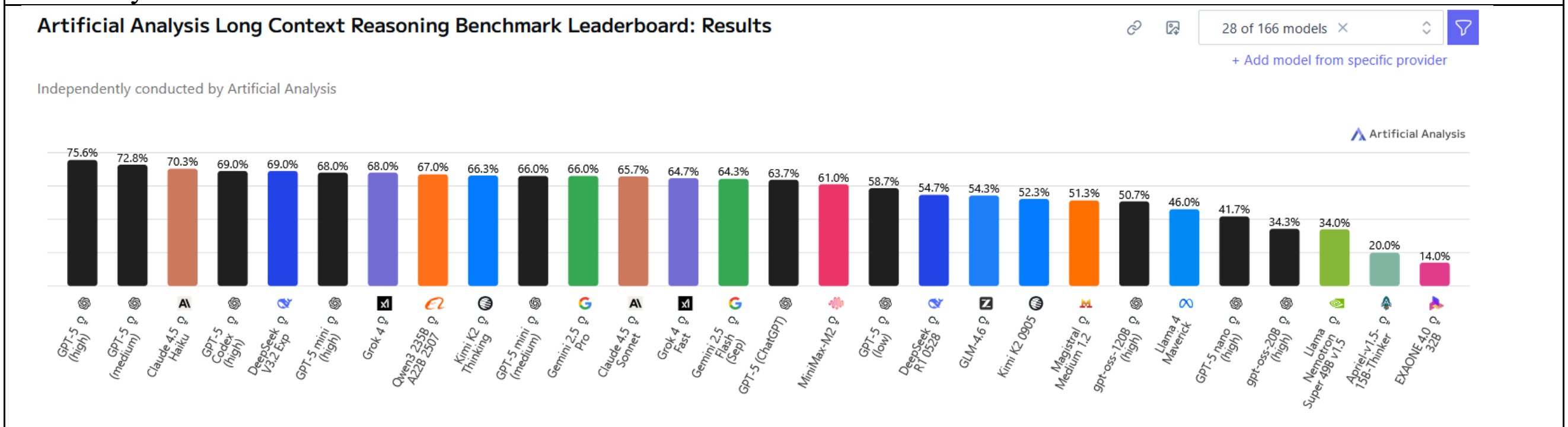

A challenging benchmark measuring language models' ability to extract, reason about, and synthesize information from long-form documents. The Long Context Reasoning Benchmark features 100 questions across diverse document types including academic papers, company financials, government consultations, legal documents, industry reports, and marketing materials, requiring genuine reasoning rather than simple data extraction.

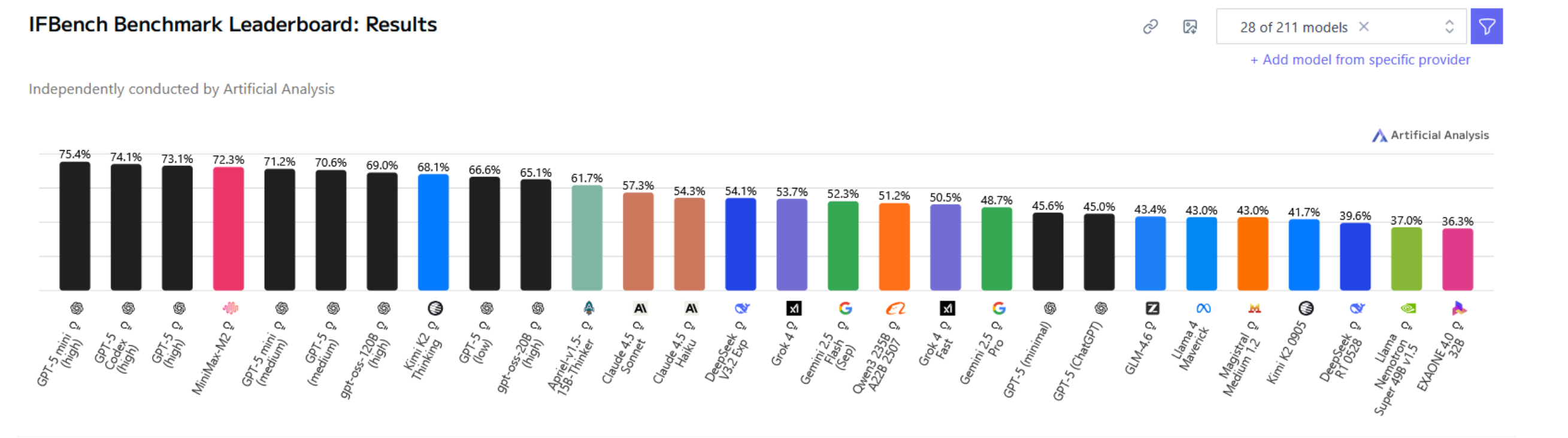

A benchmark evaluating precise instruction-following generalization on 58 diverse, verifiable out-of-domain constraints that test models' ability to follow specific output requirements. IFBench addresses the problem that current language models strongly overfit to a small set of verifiable constraints and cannot generalize well to unseen output constraints.

Section S2: End-to-end PDE derivation

- A series of experiments were conducted using ChatGPT in different modes, and the details of the tests are summarized in the sections below.
- The **attached excel file** summarizes the LLM results, including short structured summaries of the different runs including the LLM run set-up mainly from ChatGPT. Selected exploratory runs

conducted with Claude and Gemini are also presented to provide additional context regarding the applicability of the analysis across different LLM platforms.

- A summary of the principal observations from the case study, including source selection, evidence coverage, reasoning, hallucinations, prompt design, data extraction, summarization, and PDE variability is reported in the table below:

Observations	LLM-results	Comments
**Variable source selection and citation along with incomplete coverage**	Across repeated runs, the combined retrieval-and-reasoning prompt did not consistently identify all key assessment reports included in the reference evidence base, including EFSA, ANSES, EPA, and CIR documents. More comprehensive source identification was observed when retrieval was performed using dedicated search-focused prompts before downstream reasoning.	A set of 16 key sources was established through human-expert review (Section S.4.1) and used as the reference evidence base. The observation supports the need for a structured retrieval step before evidence summarization and interpretation (e.g., Gao et al., 2023; Oche et al., 2025)
**Variability in PoD selection and PDE derivation**	Different runs identified different PoDs and/or modifying factors, resulting in different PDE values. The figure below presents examples extracted from some LLM runs for oral PDE derivation and illustrates how different combinations of PoD selection and modifying factors can lead to different PDE outcomes. 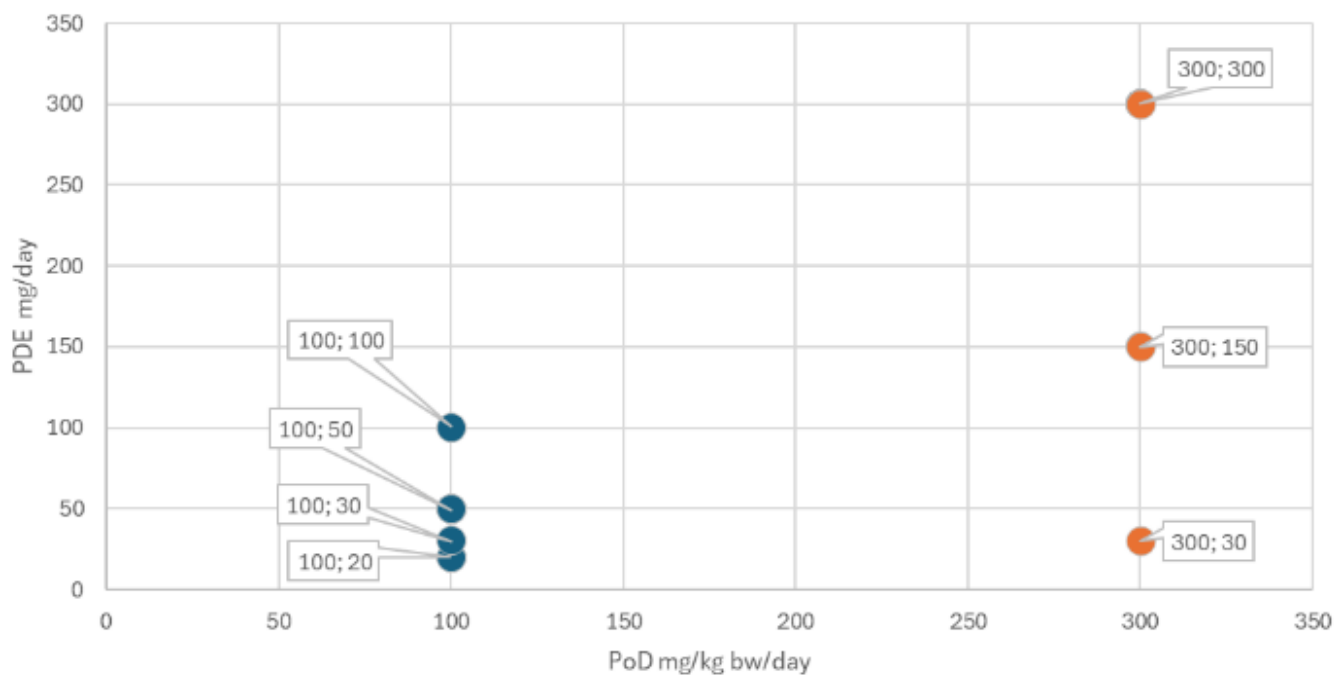	The observed variability may arise from both LLM behavior and the interpretive nature of toxicological risk assessment. PDE derivation is not fully deterministic, as alternative PoDs and modifying factors may lead to different outcomes. PDE derivations may differ among experts (Sehner et al., 2024). The observations highlight the importance of expert review when evaluating the reasoning underlying PDE derivation and the resulting health-based limits.
**Reasoning limitations**	In several runs, the model appeared to rely heavily on a single source or a limited subset of the available evidence when selecting the PoD and modifying factors. Conflicting findings across studies were not always explicitly highlighted in line with recent literature (Ge et al., 2025).	PDE derivation requires integration of evidence across multiple studies, species, endpoints, and regulatory assessments. The observations suggest that while LLMs can summarize individual documents effectively, they may not consistently perform the type of cross-document evaluation expected in expert toxicological risk assessment.
**Hallucinations/ Confabulations**	During manual review, a small number of outputs were identified in which references, quotations, or supporting statements could not be verified against the cited source documents.	Although only a limited number of such instances were observed during manual review, they may be difficult to detect without detailed source verification. This highlights the importance of expert review of extracted evidence and citations before their use in decision-making. Recent evidence confirms that fabricated or unverifiable references can be generated by LLMs and may evade routine quality-control processes if not systematically checked (Topaz et al., 2026; Zhao et al., 2026).
**Prompt design**	The completeness and quality of outputs varied depending on prompt structure. End-to-end prompts tended to provide less transparent intermediate reasoning than	The observations suggest that performance depends not only on model capability but also on workflow design. Structured

Observations	LLM-results	Comments
	stepwise prompts separating retrieval, extraction, summarization, and interpretation.	prompting may improve transparency and facilitate expert review of intermediate outputs (e.g., Wang et al., 2024; Anh-Hoang et al., 2025; Brucks and Toubia, 2025).
**Time efficiency**	LLM-assisted workflows reduced the time required for literature screening, extraction of toxicological findings, summarization of study reports, and preparation of draft assessment narratives. Tasks that would normally require manual review of lengthy reports to extract key data could often be completed within minutes.	Time savings were most apparent for information retrieval, summarization, and drafting activities (Schmidt et al., 2025; Wang et al., 2025). However, expert review remained necessary for source verification, interpretation of evidence, final PDE selection, and validation of extracted data.
**Data extraction in tables**	The model was generally effective at extracting study details and organizing information into structured tables summarizing study design, species, dose levels, critical effects, and PoDs. However, omissions were observed.	Tabular extraction can improve review efficiency but should be treated as draft output requiring verification against source documents. Performance appeared to decline as document volume and complexity increased.
**Summarization**	LLMs generated concise summaries of lengthy toxicology reports, regulatory assessments, and scientific publications. Summaries generally captured major study findings and conclusions but occasionally omitted key details.	Summarization was one of the strongest observed capabilities. One observed limitation was not linguistic quality but lack of details that could be relevant to draw conclusions. Similar strengths and limitations have been reported in LLM-assisted evidence synthesis, where models can efficiently synthesize and summarize large bodies of scientific information but may omit important details and therefore require expert review to ensure completeness and accurate interpretation (Schmidt et al., 2025; Wang et al., 2025)

S2.1. Minimal end-to-end prompt – Zero-shot prompt (with and without Deep Research)

Independent users submitted the same query ("Act as a toxicologist and derive the oral and parenteral Permitted Daily Exposure (PDE) for Acetyl Tributyl Citrate (CAS 77-90-7), providing details on the point of departure") at different times and with various ChatGPT model versions and modes, including Deep Research. Across runs, certain references were cited consistently, although the emphasis placed on each varied. Some sources required verification of origin and authorship, and several relevant documents identified through manual data collection (e.g., the ECHA dossier and the French Agency safety assessment) did not appear in the ChatGPT-generated outputs.

S2.2. Structured end-to-end prompts – Step-guided prompting (with and without Deep Research)

When more complex prompts guided the model from data discovery through PDE derivation, both the standard ChatGPT mode and the Deep Research mode produced summaries based on a restricted number of references. In-text citations facilitated verification, but the restricted set of references reduced the breadth of coverage compared with the knowledge base compiled separately. Two alternative prompts were employed. These

prompts were tested with multiple LLMs, including ChatGPT 5.0/5.2, Claude Sonnet 4.5, and Gemini 3. Minor prompt modifications were introduced where necessary to accommodate model-specific adjustments. No formal benchmarking was performed; the purpose was to assess whether the general observations were broadly consistent across different LLM platforms. The results

Structured prompt (short)

Task: You are a toxicologist with ERT and DABT certification.
You must derive the PDE for
Acetyl Tributyl Citrate (ATBC; Tri-n-butyl acetyl citrate, CAS 77-90-7)
according to the following rules.
GENERAL RULE: Always report evidence with references and citations
SPECIFIC INSTRUCTIONS:
1) Provide a background on the **chemical characteristics as well as the use** of the substance, providing a general background on the substance.
2) Briefly summarize whether **health based guidance values** are available for the substance; whether the substance is included in some specific list (e.g., GRAS, flavourings. Food additives, etc). Also pinpoint whether authorities have recognized some specific toxicity of the substance (endocrine disruptors, developmental or reproductive toxicant, sensitizer, etc).
3) Summarize any evidence related to the **pharmacological and biological activity**, including evidence including for example: Anticholinergic activity; Neurotoxic functional effects following interaction with given receptors; Other receptor or enzyme modulation relevant to toxicity or pharmacodynamics (specify receptor/enzyme if stated); Formation of reactive metabolites and in particular electrophilic species that can covalently bind to biological macromolecules.
4) Summarize any evidence related to **endocrine or hormonal activity** (e.g., estrogenic, androgenic, thyroid, steroidogenic) including in vivo and in vitro studies and highlighting whether health authority has mentioned that there is any concern for endocrine activity or disruption for this substance.
5) Summarize the **ADME properties** about: Absorption and bioavailability of the substance, reporting specific values of oral bioavailability or other related parameters; Distribution of the substance and potential for bioaccumulation; Excretion; Metabolism of the substance, highlighting any relevant metabolite mentioned and whether this is human relevant, if discussed and report if any of these metabolites are considered to be of concern.
6) Summarize the **genotoxicity and mutagenicity** potential of the substance reporting any call available from relevant reports or documents. Information on differences between in vitro and in vivo studies should be highlighted as well as conflicting information across available sources. The role of metabolic activation must also be included. Note whether some of the reported studies have some important limitations.
7) Summarize the **irritation potential** of the substance with information on the results of the irritation studies (e.g., positive, negative, inconclusive, irritant, mild irritant, corrosive) with a short description of the tests or specific considerations from physico-chemical properties. Always specify the (potential) target organ when summarizing the results (e.g., skin, eye, respiratory tract). Note whether some of the reported studies have some relevant limitations.
8) Summarize the **sensitization potential** of the substance with information regarding skin, dermal, respiratory tract, systemic, allergy, etc) for example from:
- In vitro tests such as DPRA, KeratinoSens, h-CLAT, GARD, etc;
- In vivo tests such as LLNA, Food Allergen Sensitization Models, etc;
- Acute tests reporting findings on sensitization potential;
- Human data and epidemiological data, historical data;
-Physico-chemical considerations related to the potential of sensitization (e.g., solubility, lipophilicity, hydrophilicity).
Report any potency consideration (e.g., strong, extreme, weak sensitizer) and if available the EC3 value.
9) Summarize acute toxicity by summarizing conclusions or interpretations of the different **acute toxicity** studies including:
- the threshold values like LD_{50} or LC_{50} and main clinical signs or target organ effects (e.g., neurotoxicity, respiratory distress, hepatic necrosis);
- a summary of any discussion in the document about the potency (GHS class or not other classifications related to acute toxicity)
- a summary of any noted effects occurring well below lethal doses that could indicate a specific acute hazard (e.g., cholinesterase inhibition, methemoglobinemia, renal necrosis).
- any irritation effect noted (e.g. respiratory tract)
10) Summarize **systemic toxicity** and corresponding thresholds (e.g., NOAEL, LOAEL, NOEL, LOEL, BMD, BMDL) complemented with the critical effect used to derive the threshold or whether the threshold corresponds to the highest dose tested; only focus on systemic toxicity; reporting conflicting results across sources on systemic effects.
11) Summarize **reproductive and developmental toxicity** and corresponding thresholds (e.g., NOAEL, LOAEL, NOEL, LOEL, BMD, BMDL) complemented with the critical effect used to derive the threshold or whether the threshold corresponds to the highest dose tested; only focus on reproductive and developmental toxicity; reporting conflicting results across sources on systemic effects; report whether the sources identify this chemical as a reproductive or developmental toxicant.
12) Summarize the **carcinogenicity potential** highlighting the rationale behind of the conclusions; discuss available studies or human data and epidemiological data and historical data; also pinpoint any supporting studies (e.g., New Approach Methodologies, in vitro studies, in silico predictions, read-across, etc) mentioned to support the carcinogenicity conclusion; also report any conflict of the conclusions across sources;
13) Based on the collected evidence, use your expertise to identify the **point of departure** for the derivation of a lifetime PDE for oral exposure.

14) Derive the corresponding lifetime oral PDE according to the ICHQ3E draft guidance.

Output format:
Narrative style with the following sections:
1) Background
2) Health based guidance values
3) pharmacological and biological activity
4) endocrine or hormonal activity
5) ADME properties
6) genotoxicity and mutagenicity
7) irritation potential
8) sensitization potential
9) acute toxicity
10) systemic toxicity
11) reproductive and developmental toxicity
12) carcinogenicity potential
13) point of departure
14) PDE according to the ICHQ3E

Structured prompt (long)

REGULATORY CONTEXT AND EXPERTISE LEVEL
You are acting as a **senior regulatory toxicologist** (≥15 years experience) performing a toxicological risk assessment for the European Medicines Agency (EMA) under EU pharmaceutical legislation (Directive 2001/83/EC as amended).
This assessment follows:
ICH Q3C (Residual Solvents, R2 2021)
ICH Q3D (Elemental Impurities, R1 2019)
ICH Q3E (Extractables and Leachables, Final 2025)
Your assessment will undergo peer review by regulatory toxicologists and may be scrutinized in regulatory submissions. Therefore, **transparency of reasoning**, **explicit uncertainty acknowledgment**, and **clear documentation of professional judgments** are essential.

SUBSTANCE AND APPLICATION
Substance: Acetyl Tributyl Citrate (ATBC, CAS 77-90-7)
Chemical Class: Citrate ester plasticizer, phthalate alternative
Pharmaceutical Applications:
Film coating plasticizer for oral solid dosage forms (tablets, capsules)
Component of pharmaceutical packaging materials
Potential extractable/leachable from primary packaging in contact with drug products
Regulatory Question: What are the scientifically justified Permitted Daily Exposure (PDE) values for ATBC for oral and parenteral routes that ensure patient safety over a lifetime of exposure?

ASSESSMENT FRAMEWORK: STEP-BY-STEP WITH EXPLICIT REASONING
Please structure your assessment following this framework. For each step:
STATE your decision/conclusion
EXPLAIN your reasoning (chain of thought)
FLAG any uncertainties or data gaps
IDENTIFY professional judgment points where alternatives exist
JUSTIFY why you selected one approach over alternatives

STEP 1: LITERATURE REVIEW AND DATA LANDSCAPE
Task: Identify and evaluate available toxicological data for ATBC.
Required Output:
DATA LANDSCAPE ASSESSMENT:

Available Data Categories:
☐ Acute toxicity (oral, dermal, inhalation)
☐ Repeated-dose toxicity (subchronic: 28-day, 90-day; chronic: 1-year, 2-year)
☐ Genotoxicity (in vitro, in vivo)
☐ Carcinogenicity (adequate? limited? absent?)
☐ Reproductive toxicity (fertility, developmental)
☐ ADME/Pharmacokinetics (absorption, metabolism, bioavailability)
☐ Mechanistic data (mode of action, relevance to humans)

Key Studies Identified: [List 5-10 most relevant studies with brief quality assessment]

Data Quality Assessment:
- GLP-compliant studies: [yes/no for key studies]
- Guideline studies (OECD, EPA, equivalent): [yes/no]
- Peer-reviewed publications: [yes/no]
- Gray literature (registrations, assessments): [specify]

⚠️ DATA GAPS IDENTIFIED:
[List any critical missing data and how you will address these gaps]

🔍 REGULATORY PRECEDENTS:
- EFSA assessment: [if available, cite]
- EPA assessment: [if available, cite]
- ECHA REACH dossier: [if available, cite]
- Other regulatory bodies: [if available, cite]
Chain of Thought Requirement: Explain your search strategy and how you evaluated study reliability. If multiple sources provide conflicting information, explain how you weighted the evidence.

STEP 2: POINT OF DEPARTURE (POD) SELECTION
This is a **CRITICAL PROFESSIONAL JUDGMENT POINT**. Multiple defensible PODs may exist.
Task: Identify candidate PODs and select the most appropriate one using weight-of-evidence reasoning.
Required Output:
CANDIDATE PODs IDENTIFIED:

Option A:
- Study: [citation]
- Species/Strain:
- Route: [oral gavage / dietary admixture / other - CRITICAL to specify]
- Duration:
- Endpoint: [specific effect observed]
- NOAEL: [value] mg/kg/day
- LOAEL: [value] mg/kg/day (if applicable)
- Study Quality: [GLP? Guideline? Adequate controls?]
- Strengths: [why this is a good POD]
- Weaknesses: [limitations]

Option B:
[Repeat structure for alternative POD]

Option C:
[Repeat structure if additional alternatives exist]

⚙️ PROFESSIONAL JUDGMENT: POD SELECTION

SELECTED POD: [Specify which option]

REASONING:
[Explain why you selected this POD over alternatives, considering:]
- Most sensitive endpoint (lowest NOAEL with adverse effect)
- Study quality and reliability
- Relevance to human exposure scenario
- Route of administration alignment
- Biological plausibility
- Regulatory precedent
- Conservative vs. risk-based considerations

ALTERNATIVE VIEWPOINT:
[Describe the strongest argument for why a different POD might be chosen]
[Explain why you still prefer your selected POD]

⚠️ UNCERTAINTIES:
- How confident are you that this is the critical effect? [High/Medium/Low]
- Are there uncharacterized endpoints that might be more sensitive? [Specify]
- Is this endpoint relevant to humans? [Justify]

SENSITIVITY CHECK:
If the alternative POD were used instead, how would this change the final PDE?
[Calculate to show impact of POD choice]

STEP 3: UNCERTAINTY FACTOR DERIVATION
Each factor represents a **specific source of uncertainty** requiring professional judgment.
Required Output:
UNCERTAINTY FACTOR ANALYSIS:

F1 - INTERSPECIES EXTRAPOLATION (Animal → Human)
├Test Species: [rat/mouse/dog/other]
├Default ICH Value: [typically 5 for rat, 10 for other species]
├
├ PROFESSIONAL JUDGMENT DECISION POINT:
Should we use the default, or modify based on:
- Comparative metabolism data?
- Allometric scaling considerations?
- Specific toxicokinetic information?

├SELECTED VALUE: F1 = [value]
├JUSTIFICATION: [explain reasoning]
├CONFIDENCE: [High/Medium/Low]

F2 - INTRASPECIES VARIABILITY (Human Population)
├ Default ICH Value: 10
├
├ PROFESSIONAL JUDGMENT DECISION POINT:
Should we use default, or consider:
- Sensitive populations (neonates, elderly, organ impairment)?
- Known human pharmacogenomic variability?
- Narrow vs. wide therapeutic margins?

├ SELECTED VALUE: F2 = [value]
├ JUSTIFICATION: [explain reasoning]
[Address: Are we adequately protecting pediatric population?]
CONFIDENCE: [High/Medium/Low]

F3 - STUDY DURATION EXTRAPOLATION
├ Source Study Duration: [X weeks/months/years]
├
├ ⚠ CRITICAL CLASSIFICATION DECISION:
Is this study SUBCHRONIC or CHRONIC?

Decision Criteria:
- Rodent chronic: typically ≥1 year or lifetime
- Rodent subchronic: typically 28-day, 90-day, 13-week

YOUR CLASSIFICATION: [Subchronic / Chronic]

REASONING:
[Explain how you classified this study duration]
[Note: This decision has MAJOR impact - may change PDE by 5-fold]

├ IF SUBCHRONIC → F3 typically = 2-10 (commonly 5 for 90-day studies)
├ IF CHRONIC → F3 = 1 (no extrapolation needed)

├ SELECTED VALUE: F3 = [value]
├ JUSTIFICATION: [detailed reasoning for this value]
[Consider: Does chronic data exist that could validate this choice?]
CONFIDENCE: [High/Medium/Low]

F4 - SEVERITY OF TOXICITY
├ Critical Effect Observed: [describe]

├ Severity Assessment:
☐ Severe (e.g., non-genotoxic carcinogenicity, teratogenicity)
☐ Moderate (e.g., organ damage, significant functional impairment)
☐ Mild (e.g., adaptive changes, reversible effects)

├ SELECTED VALUE: F4 = [value, typically 1 unless severe effects]
├ JUSTIFICATION: [explain severity assessment]
CONFIDENCE: [High/Medium/Low]

F5 - LOAEL TO NOAEL EXTRAPOLATION
├ Do we have a true NOAEL? [Yes/No]
├ IF NO NOAEL:
- LOAEL value: [X] mg/kg/day
- Nature of effect at LOAEL: [describe]
- Typical F5 = 3-10 depending on severity

├ SELECTED VALUE: F5 = [value, typically 1 if NOAEL available]
├ JUSTIFICATION: [explain]
CONFIDENCE: [High/Medium/Low]

COMPOSITE UNCERTAINTY FACTOR ASSESSMENT:

Total UF = F1 × F2 × F3 × F4 × F5 = [calculate]

🔍 REASONABLENESS CHECK:
- Total UF typically ranges 50-3000 for ICH PDE derivations
- Is your total UF within this range? [Yes/No]
- If outside range, explain why this is justified

⚖️ CONSERVATISM ASSESSMENT:
On a spectrum from "risk-based" to "highly precautionary":
[Risk-based] ←——————————→ [Precautionary]
↑ [Mark your position]

Explain your positioning: [Are you being appropriately conservative given uncertainties?]

STEP 4: ORAL PDE CALCULATION
Task: Calculate oral PDE with full transparency.
Required Output:
ORAL PDE DERIVATION:

FORMULA: PDE_oral = (NOAEL × Body Weight) / (F1 × F2 × F3 × F4 × F5)

INPUT PARAMETERS:
- NOAEL: [X] mg/kg/day
- Body Weight: 50 kg (ICH default - protective for adults and heavier children)
- F1: [value]
- F2: [value]
- F3: [value]
- F4: [value]
- F5: [value]
- Total UF: [product]

CALCULATION (show your work):
PDE_oral = ([X] mg/kg/day × 50 kg) / ([Total UF])
PDE_oral = [X mg] / [Total UF]
PDE_oral = [RESULT] mg/day

⚖️ REASONABLENESS CHECKS:

1. Convert to mg/kg/day for 50 kg person: [calculate]
2. Compare to source NOAEL: [X-fold below]
3. Compare to regulatory precedents:

- EFSA TDI (if available): [value and comparison]
- Other assessments: [specify]
4. Margin below NOAEL: [X-fold] - Is this adequate?

🎯 EXPECTED HUMAN EXPOSURE CONTEXT:
- Typical pharmaceutical leachable levels: [X] µg/day to [Y] mg/day
- Ratio of PDE to expected exposure: [X-fold margin]
- Is PDE likely to be exceeded in practice? [Yes/No - explain]

⚠️ UNCERTAINTIES IMPACTING ORAL PDE:
[List and briefly explain any uncertainties that could affect this value]

STEP 5: PARENTERAL PDE CALCULATION
This involves **ADDITIONAL PROFESSIONAL JUDGMENT** around route-to-route extrapolation.
Required Output:
PARENTERAL PDE DERIVATION:

⚠️ CRITICAL PRELIMINARY QUESTION:
What was the route of administration in the source POD study?

☐ ORAL (gavage, dietary, drinking water)
☐ PARENTERAL (IV, IP, SC)
☐ OTHER: [specify]

↓ [Your answer determines the extrapolation direction]

IF POD STUDY WAS ORAL:
We need to adjust DOWN for parenteral because:
- Oral: Subject to first-pass metabolism, only ~BA% reaches systemic circulation
- Parenteral: 100% reaches systemic circulation (bypasses first-pass)
- Therefore, parenteral exposure is "more potent" per mg administered

═══════════════════════════════════════

BIOAVAILABILITY (BA) ASSESSMENT:

Available BA Data:
- Species studied: [specify]
- BA value: [X]% (typically expressed as fraction: 0.XX)
- Study reference: [citation]
- Study quality: [comment on reliability]

⚠️ DATA QUALITY CHECK:
- Was this measured in the same species as POD study? [Yes/No]
- If no, what is the interspecies uncertainty?
- Was this measured after single dose or chronic dosing?
- Are there sex differences in BA?

═══════════════════════════════════════

⚙️ CRITICAL PROFESSIONAL JUDGMENT: F6 SELECTION

ICH Q3C provides guidance for route extrapolation based on BA:

ICH Q3C F6 Guidelines:
├── BA < 1%: F6 = 1 (no adjustment)
├── BA 1-50%: F6 = 10 (default conservative)
├── BA 50-100%: F6 = 2 (minimal adjustment)

For ATBC:
- Measured BA = [X]%
- Falls into: [which ICH category]
- Default ICH F6 = [value]

ALTERNATIVE APPROACH - CALCULATED F6:
If we use the measured BA directly:
F6_calculated = 1 / BA = 1 / [X] = [Y]

COMPARISON:
- ICH default F6: [value]
- Calculated F6: [value]
- Difference: [X-fold]

⚙️ DECISION POINT: Which F6 should we use?

OPTION A: Use ICH Default (F6 = 10)
Advantages:
- Regulatory precedent and acceptance
- Covers uncertainties in BA measurement
- Accounts for potential variability
- More conservative (protective)

Disadvantages:
- May be overly conservative if BA data is robust
- Could make parenteral route seem disproportionately restrictive

OPTION B: Use Calculated F6 (F6 = [Y])
Advantages:
- Scientifically precise based on actual data
- May be more appropriate if BA data is high quality
- Reduces unnecessary conservatism

Disadvantages:
- May not account for measurement uncertainty
- Less regulatory precedent
- Could be challenged by conservative reviewers

OPTION C: Hybrid Approach
Use calculated F6 but apply additional factor for uncertainty: F6 = [intermediate value]

YOUR SELECTED APPROACH: [A / B / C]

DETAILED JUSTIFICATION:
[Explain your reasoning considering:]
- Quality of BA data
- Regulatory acceptability
- Patient protection
- Scientific accuracy
- Precedent in similar assessments

⚠️ TRANSPARENCY STATEMENT:
"I acknowledge that regulatory experts might make different choices here.
The alternative approach would yield: PDE_parenteral = [X] mg/day
This represents a [Y]-fold difference from my selected value."

PARENTERAL PDE CALCULATION:

METHOD: [Describe whether using F6 in denominator or BA multiplication]

If using F6 approach:
PDE_parenteral = PDE_oral / F6
PDE_parenteral = [X] mg/day / [F6]
PDE_parenteral = [RESULT] mg/day

If using BA multiplication approach:
PDE_parenteral = PDE_oral × BA
PDE_parenteral = [X] mg/day × [BA]
PDE_parenteral = [RESULT] mg/day

FINAL VALUE: PDE_parenteral = [RESULT] mg/day

⚖️ REASONABLENESS CHECKS:

1. Ratio of oral to parenteral PDE: [X-fold]
   - Is this ratio consistent with the BA? [Yes/No - explain]

2. Parenteral PDE as % of NOAEL (for 50 kg person): [X%]
   - Typically should be << 1% of NOAEL - is it? [Yes/No]

3. Expected pharmaceutical exposures:
   - IV leachables typically: [X] µg/day to [Y] mg/day
   - Margin below PDE: [X-fold]

4. Comparison to oral PDE:
   - Parenteral is [X]× lower than oral
   - Does this make biological sense given BA? [Yes/No - explain]

STEP 6: SENSITIVITY ANALYSIS
Task: Demonstrate how alternative decisions would change the PDEs.
Required Output:
SENSITIVITY ANALYSIS:

"What if" Scenarios to Test Robustness:

SCENARIO 1: Alternative POD Selection
IF we had selected [Alternative POD from Step 2]:
- NOAEL would be: [X] mg/kg/day
- F3 might be: [different value] (due to different study duration)
- Resulting PDE_oral: [calculate]
- Resulting PDE_parenteral: [calculate]
- Change from base case: [% or fold-change]

SCENARIO 2: More Conservative F3
IF we classified the study as subchronic rather than chronic (or vice versa):
- F3 would be: [value]
- Resulting PDE_oral: [calculate]
- Resulting PDE_parenteral: [calculate]
- Change from base case: [% or fold-change]

SCENARIO 3: Alternative F6 Approach
IF we used [calculated / default] F6 instead:
- F6 would be: [value]
- Resulting PDE_parenteral: [calculate]
- Change from base case: [% or fold-change]

SCENARIO 4: Pediatric-Specific PDE (if relevant)
IF we used 10 kg body weight for neonates:
- PDE_oral: [calculate]
- PDE_parenteral: [calculate]
- This would be appropriate for: [specify products]

SENSITIVITY SUMMARY TABLE:

Scenario	PDE Oral	PDE Parenteral	Change from Base
Base Case	[X] mg/day	[Y] mg/day	Reference
Alt POD	[X] mg/day	[Y] mg/day	[±Z%]
Alt F3	[X] mg/day	[Y] mg/day	[±Z%]
Alt F6	[X] mg/day	[Y] mg/day	[±Z%]
Pediatric	[X] mg/day	[Y] mg/day	[±Z%]

ROBUSTNESS ASSESSMENT:
- Range of plausible PDE_oral values: [X] to [Y] mg/day
- Range of plausible PDE_parenteral values: [X] to [Y] mg/day
- Would these different values change regulatory conclusions? [Discuss]

STEP 7: WEIGHT OF EVIDENCE INTEGRATION
Task: Step back and evaluate the overall assessment.
Required Output:
WEIGHT OF EVIDENCE SYNTHESIS:

🔬 TOXICOLOGICAL PROFILE SUMMARY:
- Overall hazard characterization: [Low / Moderate / High toxicity]
- Key adverse effects: [list]
- Threshold vs. non-threshold toxicant: [Threshold]
- Genotoxicity: [Positive / Negative / Equivocal]
- Carcinogenicity: [Positive / Negative / Inadequate data]
- Reproductive/Developmental: [Positive / Negative / Equivocal]

🎯 CRITICAL EFFECT DRIVING PDE:
The most sensitive endpoint is: [specify]
Confidence that this is truly the critical effect: [High / Medium / Low]

📊 DATA QUALITY ASSESSMENT:
Overall quality of available data: [Excellent / Good / Adequate / Limited]
- GLP studies: [Yes/No]
- Guideline studies: [Yes/No]
- Peer review: [Yes/No]
- Multiple species: [Yes/No]
- Chronic data: [Yes/No]
- Mechanistic data: [Yes/No]

Major data gaps: [list if any]

⚖️ CONSERVATIVE VS. RISK-BASED ASSESSMENT:

Where does this assessment fall on the spectrum?

Risk-Based Precautionary
├──────────────┼──────────────┼──────────────┤
Liberal Balanced Conservative Ultra-safe
↑
[Mark position]

Justification: [Explain your positioning]

🌍 REGULATORY HARMONIZATION:
Comparison with other regulatory assessments:

Authority	Value	Type	Agreement
EFSA	[X] mg/kg	TDI	[Good/Fair/Poor]
EPA	[X] mg/kg/day	RfD	[Good/Fair/Poor]
ECHA	[comment]	DNEL	[Good/Fair/Poor]
This Assessment	[X] mg/day	PDE	Reference

Are significant discrepancies explained by:
☐ Different regulatory frameworks
☐ Different uncertainty factor applications
☐ Different POD selections
☐ Different body weight assumptions
☐ Different purposes (food vs. pharma vs. environmental)
☐ Other: [specify]

💡 BIOLOGICAL PLAUSIBILITY CHECK:
Does the derived PDE make biological sense?
- PDE is [X]-fold below the NOAEL
- This margin accounts for: [list factors]
- Human exposure at PDE level would be: [describe expected physiological impact]
- Is there any reason to expect humans would be MORE sensitive than animals? [Discuss]

- Is there any reason to expect humans would be LESS sensitive than animals? [Discuss]

STEP 8: UNCERTAINTY QUANTIFICATION AND COMMUNICATION
Task: Clearly communicate the level of confidence and remaining uncertainties.
Required Output:
UNCERTAINTY CHARACTERIZATION:

🎯 OVERALL CONFIDENCE IN PDE VALUES:

PDE_oral = [X] mg/day
Confidence Level: ● HIGH ○ MEDIUM ○ LOW

PDE_parenteral = [Y] mg/day
Confidence Level: ● HIGH ○ MEDIUM ○ LOW

[Explain what drives your confidence assessment]

DETAILED UNCERTAINTY ANALYSIS:

SOURCES OF UNCERTAINTY:

1. STUDY DESIGN UNCERTAINTIES:
⚠️ [List any limitations in the key toxicity studies]
Impact on PDE: [Minimal / Moderate / Significant]
Mitigation: [How did uncertainty factors address this?]

2. INTERSPECIES EXTRAPOLATION:
⚠️ [Discuss confidence in animal-to-human extrapolation]
- Do we have human data? [Yes/No]
- Comparative metabolism known? [Yes/No]
- Structural alerts suggesting species differences? [Yes/No]
Impact on PDE: [Minimal / Moderate / Significant]
Mitigation: [How did F1 address this?]

3. SENSITIVE POPULATIONS:
⚠️ [Are there populations of special concern?]
- Neonates: [concerns?]
- Pregnant women: [concerns?]
- Organ impairment: [concerns?]
- Genetic polymorphisms: [known?]
Impact on PDE: [Minimal / Moderate / Significant]
Mitigation: [How did F2 address this?]

4. STUDY DURATION / CHRONIC EXTRAPOLATION:
⚠️ [Confidence in subchronic-to-chronic extrapolation if applicable]
Impact on PDE: [Minimal / Moderate / Significant]
Mitigation: [How did F3 address this?]

5. ROUTE-TO-ROUTE EXTRAPOLATION:
⚠️ [Confidence in parenteral PDE derivation]
- BA data quality: [Excellent / Good / Limited]
- BA measured in same species as POD? [Yes/No]
- Choice of F6 approach: [conservative / balanced / liberal]
Impact on PDE_parenteral: [Minimal / Moderate / Significant]

6. MISSING DATA:
⚠️ [List any data that would strengthen the assessment]
- Additional chronic studies?
- Human pharmacokinetic data?
- Mechanistic data?
- Mode of action information?
Impact: [How would this change confidence?]

UNCERTAINTY COMMUNICATION:

For Regulatory Submission:
"[Write 2-3 sentences suitable for a regulatory dossier that honestly communicate the level of confidence in these PDE values]"

For Scientific Publication:
"[Write 2-3 sentences suitable for peer-reviewed literature that describe the key uncertainties and how they were addressed]"

For Risk Communication to Non-Experts:
"[Write 2-3 sentences that explain the confidence in patient safety in plain language]"

STEP 9: REGULATORY DOCUMENTATION PACKAGE
Task: Provide final summary suitable for regulatory submission.
Required Output:

FINAL PERMITTED DAILY EXPOSURE (PDE) VALUES FOR ATBC
Per ICH Q3E Guidance for European Medicines Agency

SUBSTANCE: Acetyl Tributyl Citrate (ATBC)
CAS NUMBER: 77-90-7
ASSESSMENT DATE: [Date]
ASSESSOR: [Name/Qualification]
REVIEW STATUS: [Draft / Final / Peer-Reviewed]

DERIVED PDE VALUES:

ORAL ROUTE: PDE = [X.X] mg/day
Equivalent to [Y.Y] mg/kg/day (50 kg)

PARENTERAL ROUTE: PDE = [X.X] mg/day
Equivalent to [Y.Y] mg/kg/day (50 kg)

BASIS OF DERIVATION:

Point of Departure:
- Study: [Citation]
- NOAEL: [X] mg/kg/day
- Critical Effect: [Specify]
- Route: [Oral/Other]
- Species: [Rat/Mouse/Other]

Uncertainty Factors Applied:
- F1 (Interspecies): [value] - [brief justification]
- F2 (Intraspecies): [value] - [brief justification]
- F3 (Duration): [value] - [brief justification]
- F4 (Severity): [value] - [brief justification]
- F5 (LOAEL→NOAEL): [value] - [brief justification]
- F6 (Route, parenteral only): [value] - [brief justification]
- Total UF (oral): [product]
- Body Weight: 50 kg (ICH default)

APPLICABILITY:
☑ Adult patients (≥18 years)
☑ Pediatric patients (with appropriate body weight adjustment if needed)
☑ Pregnant women
☑ Elderly patients
☐ Specific restrictions: [None / Specify if any]

EXPOSURE CONTEXT:
Expected pharmaceutical exposures from extractables/leachables:

- Oral products: [X] µg/day to [Y] mg/day
- Parenteral products: [X] µg/day to [Y] mg/day
- Margin of safety: [X]-fold below oral PDE, [Y]-fold below parenteral PDE

REGULATORY ACCEPTANCE:
- Consistent with ICH Q3E methodology: [Yes]
- Comparable to other regulatory assessments: [Discuss briefly]
- Suitable for EMA submission: [Yes / With qualifications]

KEY PROFESSIONAL JUDGMENTS:
1. [Most critical judgment point and rationale]
2. [Second key judgment and rationale]
3. [Third key judgment and rationale]

LIMITATIONS AND QUALIFICATIONS:
[List 2-4 key limitations that regulatory reviewers should be aware of]

RECOMMENDED ADDITIONAL STUDIES (if any):
☐ None required - data adequate
☐ Recommended but not essential: [Specify]
☐ Critical data gap requiring generation: [Specify]

SIGNATURE AND ATTESTATION:

I certify that this toxicological risk assessment has been performed according to ICH Q3E guidance and represents my professional scientific judgment based on the available data as of [Date].

I have disclosed all key professional judgments and uncertainties that could materially impact the derived PDE values.

Alternative interpretations of the data that could yield different PDE values have been documented in the sensitivity analysis section.

Assessor: ________________________ Date: __________
Peer Reviewer: ____________________ Date: __________ (if applicable)

STEP 10: COMPLETE REFERENCE LIST
Task: Provide full citations for all sources used.
Required Output:
REFERENCES:

[Provide complete bibliographic citations in a consistent format]

Categorize as:
- Primary toxicity studies (key POD studies)
- Supporting toxicity studies
- Pharmacokinetic/ADME studies
- Regulatory assessments and guidelines
- Review articles and secondary sources

For each key reference, note:
- Whether you accessed the primary document or relied on secondary citation
- Data quality indicators (GLP, peer-reviewed, guideline study, etc.)

ADDITIONAL INSTRUCTIONS FOR EXECUTION:
META-COGNITIVE REQUIREMENTS:
As you work through this assessment:
SHOW YOUR WORK: Don't just state conclusions - walk through your reasoning step by step
FLAG DECISION POINTS: Use the ⚙️ symbol to mark where you made a professional judgment between alternatives
ACKNOWLEDGE UNCERTAINTY: Use the ⚠️ symbol to mark where data gaps or uncertainties exist
SELF-CRITIQUE: Periodically ask yourself:
"Am I being appropriately conservative given the uncertainties?"
"Would a regulatory reviewer accept this reasoning?"

"Have I clearly explained why I chose option A over option B?"
ALTERNATIVE PERSPECTIVES: When you make a judgment call, briefly state the strongest counter-argument to your position
TRACEABILITY: Every conclusion should be traceable back to specific data with citations
OUTPUT QUALITY STANDARDS:
Your assessment should be:
✅ Transparent (a reader can follow your reasoning)
✅ Reproducible (another expert could replicate your process)
✅ Defensible (you can justify every decision)
✅ Honest about uncertainty (you don't overclaim confidence)
✅ Scientifically rigorous (appropriate methods applied correctly)
✅ Regulatory-ready (suitable for submission to EMA)
ETHICAL CONSIDERATIONS:
Remember that these PDE values will directly impact patient safety. Your assessment should:
Prioritize patient protection while avoiding unnecessary over-conservatism
Be honest about limitations and uncertainties
Not be influenced by commercial pressures to derive high or low values
Follow the science and regulatory guidance even if results are unexpected

FINAL INSTRUCTION:
Please now perform the complete toxicological risk assessment for ATBC following this structured framework. Take your time to work through each step carefully, showing all reasoning and acknowledging uncertainties. Your goal is to produce a regulatory-grade assessment that demonstrates both scientific rigor and appropriate professional judgment.
Begin with Step 1: Literature Review and Data Landscape.

S2.3. Use of a predefined knowledge base: document-constrained configuration (with and without Deep Research)

In general, the documents were uploaded using a specific prompt, after which two more detailed prompts were refined to restrict the model to using only the uploaded materials. In-text citations supported verification; however, limiting the reference set reduced the breadth of coverage compared with the separately compiled knowledge base.

Document-constrained configuration prompt

You are my RAG assistant for the documents I upload in this chat.
TASK (do this immediately after you can read the documents): - Build an internal document index and section map for all uploaded files.
- Identify and store quotable evidence (definitions, requirements, key claims, numbers) with precise location info. IMPORTANT:
- Do NOT display the index or section maps unless I explicitly ask.
- Acknowledge completion with a single line: "Documents indexed and ready."
RETRIEVAL & ANSWERING RULES:
- Before answering any question, retrieve the most relevant chunks from the uploaded documents.
- Answer using ONLY the uploaded documents unless I allow outside knowledge.
- Cite every claim with [File — section/page — anchor quote].
- If the answer is not in the documents, say so explicitly.
- Ask clarifying questions only if the query is ambiguous.
OUTPUT NOW: - Only confirm indexing is complete.

Structured prompt adjusted for document-constrained configuration

You are an experienced toxicologist with expertise as study director in toxicity studies with ERT and DABT certification.
You must act as a neutral scientific summarizer, extracting only what is explicitly stated in the uploaded documents.
You must never speculate or infer beyond the data.
Expert judgment is permitted only where explicitly requested (e.g., POD selection and PDE derivation), must be clearly labeled, and must be grounded in cited evidence.

Before drafting the narrative, identify and internally retrieve the most relevant evidence from the uploaded documents for each section.

Task:
Your task is to derive the lifetime Permitted Daily Exposure (PDE) for oral exposure for:
Acetyl Tributyl Citrate (ATBC; Tri-n-butyl acetyl citrate, CAS 77-90-7)

GENERAL RULES:
• Write one integrated narrative (no tables).
• Base all statements on cited evidence.
• Integrate evidence from multiple relevant sources; do not rely on a single report unless it is the only source available.
• Use only evidence from the uploaded documents.
• Report numeric thresholds (NOAEL, LOAEL, BMD/BMDL, LD_{50}, etc.) with units.
• Note when evidence is missing or conflicting.
• Ensure all statements are explicitly backed with citations from the uploaded documents.
• Explicitly describe the evidence-based justification for each conclusion.

SPECIFIC INSTRUCTIONS:

1. Pharmacological and biological activity – Summarize known mechanisms or receptor/enzyme interactions relevant to toxicity including Anticholinergic activity.
2. Endocrine or hormonal activity – Summarize evidence of estrogenic, androgenic, thyroid, or steroidogenic effects from in vitro and in vivo studies.
3. ADME properties – Discuss absorption (including bioavailability %), distribution (including bioaccumulation), metabolism (major enzymes and metabolites), and excretion patterns.
4. Genotoxicity and mutagenicity – Summarize in vitro and in vivo results, activation dependence, study limitations, and overall conclusions from the authors.
5. Irritation potential – Describe any irritation or corrosivity data for skin, eyes, or respiratory tract, and classification outcomes.
6. Sensitization potential – Summarize in vitro/in vivo sensitization data (skin, respiratory, systemic) and note any potency conclusions (e.g., weak, strong).
7. Acute toxicity – Summarize LD_{50}/LC_{50} values, key clinical signs, target organs, classification (e.g., GHS), and conclusions.
8. Systemic toxicity – Summarize sub-chronic/chronic findings, critical systemic effects, and numeric thresholds (NOAEL, LOAEL, BMD/BMDL).
9. Reproductive and developmental toxicity – Summarize study types, thresholds, species, endpoints, note if identified as a reproductive/developmental toxicant, and discuss concordance or conflicts across sources.
10. Carcinogenicity potential – Summarize carcinogenicity evidence (animal, human, mechanistic, in silico) and discuss concordance or conflicts across sources.
11. Point of Departure – Identify and justify the study and endpoint selected as the POD for PDE derivation, including relevance and data quality; in this step apply your expert judgement.
12. PDE derivation (ICH Q3E) – Use the ICHQ3E draft guidance to calculate the lifetime oral PDE showing the full equation, uncertainty factors (F1, F2, F3, etc) associated with considerations on interspecies, intraspecies, LOAEL→NOAEL, database, duration, etc., and perform a brief sensitivity analysis.
13. Overall conclusion – Provide a concise summary of key toxicological properties and the final recommended PDE value with confidence statement.
14. List of references used

Output format:
-Narrative style
- All statements explicitly backed with citations (e.g., EFSA, 2021 or FDA CDER 2005) from the uploaded documents
- Following sections:
1) Pharmacological and biological activity
2) Endocrine or hormonal activity
3) ADME properties
4) Genotoxicity and mutagenicity
5) Irritation potential
6) Sensitization potential
7) Acute toxicity
8) Systemic toxicity
9) Reproductive and developmental toxicity
10) Carcinogenicity potential
11) Point of departure
12) PDE according to ICHQ3E
13) Overall conclusions
14) List of references used

The long, structured prompt was also employed in the document-constrained configuration mode.

Section S3: Task-specific prompt decomposition – Modular workflow

S3.1. Data search – Example of ChatGPT prompts

ChatGPT prompt for reviews

Act as a regulatory toxicologist. Goal: Identify all authoritative risk or toxicity assessment reports **for tri-n-butyl acetyl citrate (ATBC, CAS 77-90-7)**. Search the evidence in the main text, in the tables, in the appendixes and in the supplemental material. Task: List the regulatory agencies or organizations (e.g., EFSA, ECHA, OECD, US EPA, FDA, EMA, SCCS, WHO, Health Canada, Australian authority, Swissmed, NITE Japan, etc.) that have reviewed the toxicological data for ATBC and published an assessment. For each, include the document title, year, type of assessment, main toxicological focus, and citation with hyperlink. Explicitly note if no authoritative toxicology review was identified for any major body.

ChatGPT prompt for peer-reviewed publications

Act as a toxicologist. Goal: Identify peer-reviewed publications reporting or discussing toxicity data (and in particular from genotoxicity or repeated dose toxicity or carcinogenicity, or reproductive and developmental toxicity studies), for **tri-n-butyl acetyl citrate (ATBC, CAS 77-90-7)**. Search the evidence in the main text, in the tables, in the appendixes and in the supplemental material. Task: List the publications that contain information on the toxicological data for ATBC. For each, include the document title, year, main toxicological focus, and citation with hyperlink. Explicitly note if no peer-review was identified for any major body.

ChatGPT prompt to retrieve evidence supporting the safety assessment

Act as a regulatory toxicologist and identify and summarize all authoritative evaluations, safety limits, and regulatory positions available for **tri-n-butyl acetyl citrate (ATBC, CAS RN 77-90-7).** Specifically:

1. Identify regulatory or authoritative reviews and list any toxicological assessments, scientific opinions, monographs, or evaluations conducted by recognized authorities such as EFSA, ECHA, OECD, US EPA, FDA, EMA, SCCS, WHO, Health Canada, NITE (Japan), or other comparable bodies.
2. Report existing safety limits or guidance values such as established Acceptable Daily Intake (ADI), Tolerable Daily Intake (TDI), Reference Dose (RfD), Margin of Safety (MOS), or maximum daily dosage defined by those authorities. Include the numerical value, units, and the basis for derivation (e.g., study type, critical effect, uncertainty factors).
3. Summarize the FDA's position and clearly state the regulatory context and use category and specify whether ATBC is recognized by the U.S. FDA for example as: a food additive or indirect food-contact substance, or an inactive ingredient (excipient) in pharmaceuticals, or listed in any relevant CFR section or FDA database (e.g., Inactive Ingredient Database, Food Contact Notifications, GRAS notices, Pesticides, etc).
4. Determine EFSA's assessment status and identify whether EFSA has set an ADI/TDI or other safety limit for ATBC under any applicable category, such as: Feed additives (nutritional, sensory, or technological), food additives, flavorings, processing aids, food contact materials (FCMs), contaminants or other chemical groups (e.g., mycotoxins, natural plant products, heavy metals, POPs).
5. Include citations and active links and cite the authoritative source (e.g., EFSA Journal, ECHA dossier, EPA IRIS entry, FDA database record, or official government report).
6. Present results in a structured regulatory summary table with the following columns: A) Authority / Organization; B) Assessment Type / Document Title; C) Year; D) Main Findings or Toxicological Conclusions including safety limits if any (ADI, TDI, RfD, etc.); D) Regulatory Context (e.g., flavoring, additive, excipient, etc.) E) Citation / Source (with hyperlink). If no value or evaluation is available from a specific authority, explicitly state **"No data or assessment located"** for transparency.

S3.2 Information extraction – Example of ChatGPT prompts

Prompt for Pharmacological/Biological Data

**Extract information related to Pharmacological/Biological activity from a given document**
**Role:** You are a pharmacologist and a toxicologist with ERT and DABT certification. You must act as a neutral scientific summarizer, **EXTRACTING** only what is explicitly stated in the uploaded document. You must never speculate, infer, or summarize the absence of data. You must comply with the following rules exactly.<br><br>**Task:** Extract only from the uploaded document (main text, appendices, tables, or supplemental material) any data on pharmacological or biological activity of:<br>Acetyl Tributyl Citrate (ATBC; Tri-n-butyl acetyl citrate, CAS 77-90-7)

according to the following rules.

Strictly follow these **GENERAL RULES**:

- Use only information explicitly present in the uploaded document (main text, appendices, tables, or supplemental material). Do not infer missing data or introduce external context.
- If the document contains no explicit mention of a relevant activity or finding, omit it entirely and do not state or imply that it was not found, tested, or reported.
- Exclude all evidence related to genotoxicity.
- Do not combine, harmonize, or compare results across studies, and do not reference findings or interpretations from any other section of the summary.
- Assign each finding a unique identifier using the document filename as prefix and a sequential suffix (example: DocumentName_Biol1, DocumentName_Biol2, etc).
- Extract the source document details including authors, title of the report/article, year.
- Create a short reference for the uploaded documents such as "Author, year" (Author, 2010) or "Short title, year".

Strictly follow these **INSTRUCTIONS** AND include findings only if they address:

- Endocrine or hormonal activity (e.g., estrogenic, androgenic, thyroid, steroidogenic)
- Anticholinergic activity
- Other receptor or enzyme modulation relevant to toxicity or pharmacodynamics
- Neurotoxic functional effects following interaction with given receptors
- Formation of reactive metabolites and in particular electrophilic species that can covalently bind to biological macromolecules

For each relevant finding as described in the document, briefly summarize evidence **on:**

- Test Type (in vivo, in vitro, in silico, modelling, docking)
- Biological Activity or Mechanism
- Test System / Species
- Observed Effect(s) and conclusions on the study (if any) as stated in the uploaded document (summarize in less than 100 words) including any other considerations of the authors that support interpretation of the study
- Citations included in the document such as primary references, study names, etc., e.g., Finkelstein and Gold (1959).

Output format:
Create the following table:

| Identifier | Source document details (title, authors, year) | Short Reference | Test Type | Biological Activity / Mechanism | Test System / Species | Observed Effect(s) & conclusions | Citations |

If **no relevant evidence** is found, output only: "No evidence in the document on pharmacological and biological activity."

Prompt for Toxicokinetics

Extract information related to Toxicokinetics from a given document

Role: You are an expert on toxicokinetics and ADME (Absorption, Distribution, Metabolism, Excretion). You must act as a neutral scientific summarizer, **EXTRACTING** only what is explicitly stated in the uploaded document. You must never speculate, infer, or summarize the absence of data. You must comply with the following rules exactly.

Task: Extract only from the uploaded document (main text, appendices, tables, or supplemental material) any data on toxicokinetics (or pharmacokinetics) and ADME (Absorption, Distribution, Metabolism, Excretion) of:
Acetyl Tributyl Citrate (ATBC; Tri-n-butyl acetyl citrate, CAS 77-90-7)
according to the following rules.

Strictly follow these **GENERAL RULES**:

- Use only information explicitly present in the uploaded document (main text, appendices, tables, or supplemental material). Do not infer missing data or introduce external context.
- If the document contains no explicit mention of a relevant activity or finding, omit it entirely and do not state or imply that it was not found, tested, or reported.
- Assign the document a unique identifier using the document filename as prefix and "ADME" as suffix: DocumentName_ADME.
- Extract the source document details including authors, title of the report/article, year.
- Create a short reference for the uploaded documents such as "Author, year" (Author, 2010) or "Short title, year".

Strictly follow these **INSTRUCTIONS**:

- The goal is to extract only information on **internal exposure and absorption, bioavailability, distribution, metabolism, or excretion** following exposure (e.g., oral, dermal, inhalation, parenteral, ocular).
- Exclude any studies limited to external exposure, migration, or leaching
- Create a summary section about **Absorption** according to the following rules:

- Summarize in less than 150 words the conclusions drawn in the document on the absorption and bioavailability of the substance, reporting specific values of oral bioavailability or other related parameters, and highlighting any study mentioned to draw the conclusions. Report any information on dermal absorption, if any. If there is no evidence about absorption or bioavailability, then simply write "No evidence in the document".
- Extract any citations associated with the summarized **Absorption** evidence, , e.g., primary references such as Finkelstein and Gold (1959) or study names, etc.

- Create a summary section about **Distribution** according to the following rules:

- Summarize in less than 50 words the conclusions of the document on the distribution of the substance highlighting whether the document mentions any potential for bioaccumulation. Shortly summarize the studies used to draw conclusions on distribution and bioaccumulation, if any. If there is no evidence about distribution, then simply write "No evidence in the document".
- Extract any citations associated with the summarized **Distribution** evidence, e.g., primary references such as Finkelstein and Gold (1959) or study names, etc.

- Create a summary section about **Metabolism** according to the following rules:

- Summarize in less than 150 words the metabolism of the substance highlighting any study used in the document to get insights on metabolism. Highlight any relevant metabolite mentioned and whether this is human relevant, if discussed. Report if any of these metabolites are considered to be of concern. If there is no evidence about metabolism, then simply write "No evidence in the document".
- Extract any citations associated with the summarized **Metabolism** evidence, e.g., primary references such as Finkelstein and Gold (1959) or study names, etc.

- Create a summary about **Excretion** according to the following rules:

- Summarize in less than 50 words the conclusions of the document on the excretion of the substance highlighting any study mentioned to draw such conclusions. If there is no evidence about excretion, then simply write "No evidence in the document".

- Extract any citations associated with the summarized **Excretion** evidence, e.g., primary references such as Finkelstein and Gold (1959) or study names, etc.

Output format:
Create the following table:
| Document unique identifier | Source document details (title, authors, year) | Short reference | Section type (Absorption or Distribution or Metabolism or Excretion) | Summary | Citations |

If you do not find anything in the documents, then simply write "No conclusions in the document on toxicokinetics/ADME"

Prompt for Genotoxicity

Extract in vitro studies from a given document	Extract in vivo studies from a given document	Extract genotoxicity call from a given document
**Role:** You are an experienced genetic toxicologist with expertise in evaluating in vitro genotoxicity and in vitro mutagenicity studies. You must act as a neutral scientific summarizer, **EXTRACTING** only what is explicitly stated in the uploaded document. You must never speculate, infer, or summarize the absence of data. You must comply with the following rules exactly.<br><br>**Task:** Extract only from the uploaded document (main text, appendices, tables, or supplemental material) any finding from in vitro genotoxicity and mutagenicity studies of:<br>Acetyl Tributyl Citrate (ATBC; Tri-n-butyl acetyl citrate, CAS 77-90-7)<br>according to the following rules.<br><br>Strictly follow these <u>**GENERAL RULES**</u>:<br>• Use only information explicitly present in the uploaded document (main text, appendices, tables, or supplemental material). Do not	**Role:** You are an experienced genetic toxicologist with expertise in evaluating in vivo genotoxicity studies. You must act as a neutral scientific summarizer, **EXTRACTING** only what is explicitly stated in the uploaded document. You must never speculate, infer, or summarize the absence of data. You must comply with the following rules exactly.<br><br>**Task:** Extract only from the uploaded document (main text, appendices, tables, or supplemental material) any finding from in vivo genotoxicity and mutagenicity studies of:<br>Acetyl Tributyl Citrate (ATBC; Tri-n-butyl acetyl citrate, CAS 77-90-7)<br>according to the following rules.<br><br>Strictly follow these <u>**GENERAL RULES**</u>:<br>• Use only information explicitly present in the	**Role:** You are an experienced genetic toxicologist with ERT and DABT certification. You must act as a neutral scientific summarizer, **EXTRACTING** only what is explicitly stated in the uploaded document. You must never speculate, infer, or summarize the absence of data. You must comply with the following rules exactly<br><br>**TASK:** Extract only from the uploaded document (main text, appendices, tables, or supplemental material) any genotoxicity evidence (both in vitro and in vivo) of:<br>Acetyl Tributyl Citrate (ATBC; Tri-n-butyl acetyl citrate, CAS 77-90-7)<br>according to the following rules.<br><br>Strictly follow these <u>**GENERAL RULES**</u>:<br>• Use only information explicitly present in the uploaded document (main text, appendices, tables, or supplemental material). Do not infer missing data or introduce external context.<br>• If the document contains no explicit mention of a relevant activity or finding,

<table>
<tr>
<td>infer missing data or introduce external context.
• If the document contains no explicit mention of a relevant activity or finding, omit it entirely and do not state or imply that it was not found, tested, or reported.
• Exclude evidence related to in vivo genotoxicity or carcinogenicity.
• Exclude in vitro assays unrelated to genotoxicity or mutagenicity (e.g., cytotoxicity, receptor binding, enzyme inhibition).
• Assign each study finding a unique identifier using the document filename as prefix and a sequential suffix (example: DocumentName_Geninvitro1, DocumentName_ Geninvitro2, etc).
• Extract the source document details including authors, title of the report/article, year.
• Create a short reference for the uploaded documents such as “Author, year” (Author, 2010) or “Short title, year”.

Strictly follow these <u>INSTRUCTIONS:</u>

1) Include findings only if they address in vitro genotoxicity and in vitro mutagenicity testing such as:
-Bacterial reverse mutation (Ames test);
-Mammalian cell gene mutation (e.g., HPRT, TK assays);
-Chromosomal aberration or micronucleus tests;
-Other mechanistic in vitro assays that assess genotoxic or mutagenic potential.

2) For each relevant identified in vitro finding, extract and briefly summarize evidence on:
- Study type: e.g., Ames test, In Vitro Mammalian Cell Gene Mutation Test, In Vitro Micronucleus Test, etc.
- Experimental design: including species/strains or cell lines, use of metabolic activation (e.g., S9), concentration range or maximum concentration tested.
- Findings and interpretation: summarize the reported results, indicating whether the document assigns a positive, negative, equivocal, or conflicting call to the study finding and also note any author interpretations (e.g., comments on the study, BMDL if available).
- Reliability: summarize (in less than 30 words) whether the study explicitly reports compliance with an OECD test guideline or other quality standards (if such information is not stated, provide an informed judgment</td>
<td>uploaded document (main text, appendices, tables, or supplemental material). Do not infer missing data or introduce external context.
• If the document contains no explicit mention of a relevant activity or finding, omit it entirely and do not state or imply that it was not found, tested, or reported.
• Exclude evidence related to carcinogenicity or in vitro genotoxicity.
• Exclude in vivo studies unrelated to genotoxicity or mutagenicity (e.g., carcinogenicity).
• Assign each study finding a unique identifier using the document filename as prefix and a sequential suffix (example: DocumentName_Geninvivo1, DocumentName_ Geninvivo2, etc).
• Extract the source document details including authors, title of the report/article, year.
• Create a short reference for the uploaded documents such as “Author, year” (Author, 2010) or “Short title, year”

Strictly follow these <u>INSTRUCTIONS:</u>

1) Include findings only if they address in vivo genotoxicity and in vivo mutagenicity testing such as:
- In Vivo Micronucleus Test (bone marrow or peripheral blood);
- In Vivo Comet (Single-Cell Gel Electrophoresis) Assay;
- Unscheduled DNA Synthesis (UDS) in vivo;
- Transgenic Rodent Assay;
- Chromosomal Aberration Test in bone marrow;
- Other in vivo studies clearly addressing genotoxicity;

2) For each relevant identified in vivo finding, extract and briefly summarize:
- Study type: e.g., In Vivo Micronucleus Test, In Vivo Comet Assay, Transgenic Rodent Mutation Assay, etc.
- Experimental design: including species/strains, concentration range, dose levels and exposure, duration, target tissues examined (e.g., bone marrow, liver, blood, stomach, testis).
- Findings and interpretation: summarize the reported results, indicating whether the document assigns</td>
<td>omit it entirely and do not state or imply that it was not found, tested, or reported.
• Exclude evidence related to carcinogenicity and any other study unrelated to genotoxicity or mutagenicity.
• Assign the document a unique identifier using the document filename as prefix and “Gen” as suffix: DocumentName_Gen
• Extract the source document details including authors, title of the report/article, year.
• Create a short reference for the uploaded document such as “Author, year” (Author, 2010) or “Short title, year”.

Strictly follow these <u>INSTRUCTIONS</u> if information of genotoxicity potential is present:

1) Extract and summarize (in fewer than 200 words) the conclusions on mutagenicity and genotoxicity including:
-interpretations regarding the reported genotoxicity studies (both in vitro and in vivo), indicating whether the document assigns an overall positive, negative, equivocal (or inconclusive), or conflicting call for both genotoxicity and mutagenicity to the overall findings from all the studies;
- the main rationale followed by the authors substantiating the conclusions;
- any difference between the in vitro evidence and the in vivo evidence or across the different studies;
- how metabolic activation affected the results;
- any insights on underlying mechanisms of mutagenicity and genotoxicity;
- any comment from the authors on reliability or limitations of the studies that could impact the overall conclusions;
- any additional notes from the authors such as BMDL values, conflicting results from other sources.

2) Extract any citations (e.g., primary references, references or study names included in the document) associated with the summarized evidence and create a list of citations (e.g., Finkelstein and Gold (1959), Jonker and Hollanders (1991)).

Output format:
Create the following table:
| Document unique identifier | Source document details (title, authors, year) | Short reference | Summary | Citations |

If you do not find anything in the documents on genotoxicity, then simply write "No conclusions in the document on genotoxicity "</td>
</tr>
</table>

<table>
<tr>
<td>on whether the study appears to resemble an OECD guideline design, and clearly identify and label this as “inferred judgment”); also specify whether the study was explicitly described in the document as “pre-GLP,” “guideline-like,” “for information only,” “adequate,” or “limited in reliability”; always make it explicit in each case whether the reliability designation was taken directly from the source text or inferred based on the reported design and methods.
- Citations: citations as included in the document such as primary references, study names, etc., (e.g., Finkelstein and Gold (1959)) associated with the evidence summarized.

Output format:
Create the following table:

| Identifier | Source document details (title, authors, year) | Short Reference | Study type | Experimental design | Findings and interpretation | Reliability | Citations |

If no in in vitro genotoxicity studies are found, state clearly: “No in vitro genotoxicity or mutagenicity studies were identified in the document.”</td>
<td>a positive, negative, equivocal, or conflicting call to the study finding and also note any author interpretations (e.g., comments on the study, BMDL if available).
- Reliability: summarize (in less than 30 words) whether the study explicitly reports compliance with an OECD test guideline or other quality standards (if such information is not stated, provide an informed judgment on whether the study appears to resemble an OECD guideline design, and clearly identify and label this as “inferred judgment”); also specify whether the study was explicitly described in the document as “pre-GLP,” “guideline-like,” “for information only,” “adequate,” or “limited in reliability”; always make it explicit in each case whether the reliability designation was taken directly from the source text or inferred based on the reported design and methods.
- Citations: citations as included in the document such as primary references, study names, etc., (e.g., Finkelstein and Gold (1959)) associated with the evidence summarized.

Output format:
Create the following table:

| Identifier | Source document details (title, authors, year) | Short Reference | Study type | Experimental design | Findings and interpretation | Reliability | Citations |

If no in in vivo genotoxicity studies are found, state clearly: “No in vivo genotoxicity or mutagenicity studies were identified in the document.”</td>
<td></td>
</tr>
</table>

Prompt for Sensitization and irritation

The prompts below address the sensitization potential; similar prompts can be used for the irritation potential.

<table>
<tr>
<th>Extract sensitization studies from a given document</th>
<th>Extract sensitization call from a given document</th>
</tr>
<tr>
<td>Role: You are an experienced toxicologist with expertise in evaluating the sensitization potential of chemicals. You must act as a neutral scientific summarizer, EXTRACTING only what is explicitly stated in the uploaded document. You must never speculate, infer, or summarize the absence of data. You must comply with the following rules exactly.

Task: Extract only from the uploaded document (main text, appendices, tables, or supplemental material) any evidence and findings related to sensitization potential for:
Acetyl Tributyl Citrate (ATBC; Tri-n-butyl acetyl citrate, CAS 77-90-7)
according to the following rules.

Strictly follow these <u>GENERAL RULES</u>:</td>
<td>Role: You are an experienced toxicologist with expertise in evaluating the sensitization potential of chemicals. You must act as a neutral scientific summarizer, EXTRACTING only what is explicitly stated in the uploaded document. You must never speculate, infer, or summarize the absence of data. You must comply with the following rules exactly.

Task: Extract only from the uploaded document (main text, appendices, tables, or supplemental material) any evidence and findings related to sensitization potential for:
Acetyl Tributyl Citrate (ATBC; Tri-n-butyl acetyl citrate, CAS 77-90-7)
according to the following rules.

Strictly follow these <u>GENERAL RULES</u>:</td>
</tr>
</table>

• Use only information explicitly present in the uploaded document (main text, appendices, tables, or supplemental material). Do not infer missing data or introduce external context.<br>• If the document contains no explicit mention of a relevant activity or finding, omit it entirely and do not state or imply that it was not found, tested, or reported.<br>• Focus on the sensitization potential based on findings, considerations or other evidence from in vivo studies, in vitro tests, human data and epidemiological data, historical data, or physico-chemical considerations related to the sensitization potential.<br>• Exclude evidence related to the irritation and corrosion potential.<br>• Assign each finding, consideration or other evidence, a unique identifier using the document filename as prefix and a sequential suffix (example: DocumentName_Sens1, DocumentName_ Sens2, etc).<br>• Extract the source document details including authors, title of the report/article, year.<br>• Create a short reference for the uploaded documents such as "Author, year" (Author, 2010) or "Short title, year".<br><br>Strictly follow these **INSTRUCTIONS:**<br><br>1) Include only findings, considerations or other evidence if they address the sensitization potential (e.g., skin, dermal, respiratory tract, systemic, allergy, etc) for example from:<br>- In vitro tests such as DPRA, KeratinoSens, h-CLAT, GARD, etc;<br>- In vivo tests such as LLNA, Food Allergen Sensitization Models, etc;<br>- Acute tests reporting findings on sensitization potential;<br>- Human data and epidemiological data, historical data;<br>-Physico-chemical considerations related to the potential of sensitization (e.g., solubility, lipophilicity, hydrophilicity).<br><br>2) For each identified findings, considerations or other evidence, extract and briefly summarize:<br>- **Study type**: Specify whether it is an in vitro test, or in vivo study or epidemiological study or human data or historical data or considerations on physicochemical properties and report the type of study e.g. Local Lymph Node Assay, Direct Peptide Reactivity Assay.<br>- **Experimental design**: For in vitro test report the species/strains or cell lines, and concentration range or maximum concentration tested; for the in vivo test report species/strains, route of exposure, doses, duration; for the other cases summarize the study design or considerations.<br>- **Findings and interpretation**: Summarize the reported results and considerations, indicating whether the document assigns a positive, negative, equivocal, or conflicting call; for positive results, specify whether the document indicates any potency class, e.g. strong/extreme sensitizer, weak sensitizer or GHS class or CLP class, including GHS class 1, 2, 3, etc or not classified); summarize any author's interpretations including whether the authors note whether the effects are transient or weak; report any dose or threshold value such as EC3 (with units); it is important to discuss in details any human data.<br>- **Reliability**: Summarize (in less than 30 words) whether the study explicitly reports compliance with an OECD test guideline or other quality standards (if such information is not stated,	• Use only information explicitly present in the uploaded document (main text, appendices, tables, or supplemental material). Do not infer missing data or introduce external context.<br>• If the document contains no explicit mention of a relevant activity or finding, omit it entirely and do not state or imply that it was not found, tested, or reported.<br>• Focus on the sensitization potential based on findings, considerations or other evidence from in vivo studies, in vitro tests, human data and epidemiological data, historical data, or physico-chemical considerations related to the sensitization potential.<br>• Exclude evidence related to the irritation and corrosion potential<br>• Assign the document a unique identifier using the document filename as prefix and "Sens" as suffix: DocumentName_Sens<br>• Extract the source document details including authors, title of the report/article, year.<br>• Create a short reference for the uploaded documents such as "Author, year" (Author, 2010) or "Short title, year".<br><br>Strictly follow these **INSTRUCTIONS:**<br><br>1) Include only findings, considerations or other evidence if they address the sensitization potential (e.g., skin, dermal, respiratory tract, systemic, allergy, etc) for example from:<br>- In vitro tests such as DPRA, KeratinoSens, h-CLAT, GARD, etc;<br>- In vivo tests such as LLNA, Food Allergen Sensitization Models, etc;<br>- Acute tests reporting findings on sensitization potential;<br>- Human data and epidemiological data, historical data;<br>-Physico-chemical considerations related to the potential of sensitization (e.g., solubility, lipophilicity, hydrophilicity).<br><br>**2) Generate the overall conclusions** by summarizing (in fewer than 200 words) the authors' conclusions or interpretations of relevant in vivo studies, in vitro tests, human data and epidemiological data, or physico-chemical considerations regarding the sensitization potential and including:<br>- Indicating whether the authors of the document consider the overall findings as positive, negative, or inconclusive (or equivocal);<br>- Specifying whether the document indicates any potency class, e.g. strong/extreme sensitizer, weak sensitizer or GHS class or CLP class, including GHS class 1, 2, 3, etc or not classified);<br>- Summarizing the main rationale underlying the conclusion of the sensitization potential as stated in the document, highlighting the target organ (skin, respiratory or systemic);<br>- Reporting any threshold value such as EC3 related to the sensitization potential as stated in the document;<br>- Reporting any comment from the authors on reliability or limitations of the studies that could impact on the overall conclusions;<br>- Any additional notes from the authors such as conflicting results from other sources.<br><br>3) Extract any citations (e.g., primary references, references or study names included in the document) associated with the summarized evidence and create a list of citations (e.g., Finkelstein and Gold (1959), Jonker and Hollanders (1991)).

provide an informed judgment on whether the study appears to resemble an OECD guideline design, and clearly identify and label this as "inferred judgment"); also specify whether the study was explicitly described in the document as "pre-GLP," "guideline-like," "for information only," "adequate," or "limited in reliability"; always make it explicit in each case whether the reliability designation was taken directly from the source text or inferred based on the reported design and methods.<br>- **Citations**: citations as included in the document such as primary references, study names, etc., (e.g., Finkelstein and Gold (1959)) associated with the evidence summarized.<br><br>**Output format:**<br>**Create the following table:**<br>| Identifier | Source document details (title, authors, year) | Short Reference | Study type | Experimental design | Findings and interpretation | Reliability | Citations | <br>If the document does not report sensitization studies or considerations, state clearly: "No evidence regarding sensitization potential was identified in the document."	**Output format:**<br>**Create the following table:**<br>| Document unique identifier | Source document details (title, authors, year) | Short Reference | Summary | Citations | <br>If the document does not report sensitization studies or considerations, state clearly: "No evidence regarding sensitization potential was identified in the document."

Key toxicological data (systemic toxicity, DART, carcinogenicity)

**Extract key toxicological studies from a given document**	**Extract conclusions on point of departures (if any) and systemic toxicity, developmental and reproductive toxicity, and carcinogenicity from a given document**
**Role:** You are an experienced toxicologist with expertise as study director in toxicity studies with ERT and DABT certification. You must act as a neutral scientific summarizer, **EXTRACTING** only what is explicitly stated in the uploaded document. You must never speculate, infer, or summarize the absence of data. You must comply with the following rules exactly.<br><br>**Task:** Extract only from the uploaded document (main text, appendices, tables, or supplemental material) any study related to repeated-dose, subchronic, chronic, carcinogenicity, and reproductive/developmental toxicity for different routes of administration (e.g., oral, dermal, cutaneous, inhalation, parenteral, intramuscular, subcutaneous, intravenous, intradermal) for:<br>Acetyl Tributyl Citrate (ATBC; Tri-n-butyl acetyl citrate, CAS 77-90-7)<br>according to the following rules.<br><br>Strictly follow these <u>**GENERAL RULES**</u>:<br>• Use only information explicitly present in the uploaded document (main text, appendices, tables, or supplemental material). Do not infer missing data or introduce external context.<br>• If the document contains no explicit mention of a relevant activity or finding, omit it entirely and do not state or imply that it was not found, tested, or reported.<br>• Describe each study independently and in full detail. Do not combine, harmonize, or compare results across studies, and do not reference findings or interpretations from any other section of the summary.<br>• Include in vivo data, animal data, human data and epidemiological data.<br>• Exclude in vitro assays (e.g., cell types, inhibition assay, functional assay).	**Role:** You are an experienced toxicologist with expertise as study director in toxicity studies with ERT and DABT certification. You must act as a neutral scientific summarizer, **EXTRACTING** only what is explicitly stated in the uploaded document. You must never speculate, infer, or summarize the absence of data. You must comply with the following rules exactly.<br><br>**Task:** Using only the uploaded document (main text, appendices, tables, or supplemental material), summarize the toxicity effects and corresponding thresholds from any study related to repeated-dose, subchronic, chronic, carcinogenicity, and reproductive/developmental toxicity for different routes of administration (e.g., oral, dermal, cutaneous, inhalation, parenteral, intramuscular, subcutaneous, intravenous, intradermal) for:<br>Acetyl Tributyl Citrate (ATBC; Tri-n-butyl acetyl citrate, CAS 77-90-7)<br>according to the following rules.<br><br>Strictly follow these <u>**GENERAL RULES**</u>:<br>• Use only information explicitly present in the uploaded document (main text, appendices, tables, or supplemental material). Do not infer missing data or introduce external context.<br>• If the document contains no explicit mention of a relevant activity or finding, omit it entirely and do not state or imply that it was not found, tested, or reported.<br>• Include in vivo data, animal data, human data and epidemiological data.<br>• Exclude in vitro assays (e.g., cell types, inhibition assay, functional assay).<br>• Exclude any study on acute toxicity (single dose administration), genotoxicity, irritation and corrosion, and sensitization.<br>• Use a narrative form to summarize the results.

- Exclude any study on acute toxicity (single dose administration), genotoxicity, irritation and corrosion, and sensitization.
- Assign each study a unique identifier using the document filename as prefix and a sequential suffix (example: DocumentName_Tox1, DocumentName_Tox2, etc).
- Extract the source document details including authors, title of the report/article, year.
- Create a short reference for the uploaded documents such as "Author, year" (Author, 2010) or "Short title, year".

Strictly follow these **INSTRUCTIONS**

1) Identify all relevant toxicity studies related to repeated-dose, subchronic, chronic, carcinogenicity, and reproductive/developmental toxicity for different routes of administration (e.g., oral, dermal, cutaneous, inhalation, parenteral, intramuscular, subcutaneous, intravenous, intradermal, cutaneous, ocular) for Tri-n-butyl acetyl citrate (ATBC, CAS RN 77-90-7).

2) For each study, extract and summarize the following mirroring as much as possible as reported in the document:
- **Study type** (e.g., subchronic, chronic, reproductive, developmental, carcinogenicity, combined, etc.);
- **Experimental design**: Extract duration (e.g., days, weeks, year), species, strain (if reported), number and sex of animals per group, route of exposure (e.g., oral, dermal, inhalation), and tested dose levels and specify details of the dose administration including frequency per week (when reported for example 5 days a week for 90 days) and estimated intakes for diets; generation of animals (e.g., F0, F1, F2, etc);
- **Findings**: Extract and summarize in a narrative (≤200 words) the effects observed at the lowest dose tested in this study. Include target organs, clinical signs, clinical pathology, and any context or interpretation provided by the authors such as adversity, human relevance, reversibility, or whether responses were transient or adaptive. State the lowest dose with correct units. Then list additional effects seen at higher doses, and clearly indicate if no effects were observed at the highest dose tested.
- **Interpretations:** Extract and list every threshold value reported for this study, including NOAEL, LOAEL, NOEL, LOEL, BMD, BMDL, TD50, or any similar thresholds; for each, provide: threshold type (systemic, reproductive, developmental, maternal, etc.), value exactly as written with units, the critical effect used to derive the threshold or whether the threshold corresponds to the highest dose tested; apply these extraction rules:
A) capture every occurrence of these thresholds, even if duplicated, conflicting, or restated in different sections (e.g., detailed results, summary tables, discussions, or conclusions);
B) do not reconcile, infer, or choose among conflicting values;
C) if a later section restates, revises, or contradicts a threshold for the same study, include both the original and the later value, clearly labeled by location;
D) clearly differentiate the various types of thresholds, such as: NOAEL/LOAEL/NOEL/LOEL for systemic toxicity, NOAEL/LOAEL/NOEL/LOEL for reproductive toxicity, NOAEL/LOAEL/NOEL/LOEL for developmental toxicity, NOAEL/LOAEL/NOEL/LOEL for maternal toxicity;
E) Confirm completeness across the entire document.
F) make sure to report any threshold values such as NOAEL/LOAEL/NOEL/LOEL/BMDL/TD50 values with their corresponding units.
G) for the same study, report different thresholds derived by different organizations or bodies, as stated in the document such

- Assign the document a unique identifier using the document filename as prefix and "Tox-Sys" as suffix: DocumentName_Tox-Sys
- Extract the source document details including authors, title of the report/article, year.
- Create a short reference for the uploaded documents such as "Author, year" (Author, 2010) or "Short title, year".

Strictly follow these **INSTRUCTIONS**

Generate a summary (narrative form) for the toxicity conclusions for Tri-n-butyl acetyl citrate (ATBC, CAS RN 77-90-7), formatted into the following 6 sections:

1) POD (Point of Departure) Section
If the authors identify a point of departure (NOAEL, LOAEL, LOEL, BMDL, dose level, etc) to derive safety limits (e.g., TDI, ADI, RfD, PDE, OEL, etc) or margins of safety for Tri-n-butyl acetyl citrate (ATBC, CAS RN 77-90-7), specify the following:
- the safety limit derived (e.g., TDI, ADI, RfD, PDE, OEL, etc) with units;
- the corresponding value of the dose level or threshold (e.g., NOAEL, LOAEL, LOEL, BMDL, dose level, etc) used to derive such safety limit,
- the type of dose level or threshold selected, e.g. NOAEL (orLOAEL, LOEL, BMDL, dose level, etc) from systemic effects (or reproductive, developmental, maternal, etc.);
- the critical effect used to derive the dose level or threshold;
- whether the dose level or threshold correspond to the highest dose tested;
- a short description of the supporting study (species, duration, exposure route).

If point of departures or multiple safety limits are reported, list each separately with the corresponding details.

Include relevant in-text citations provided in the documents to primary references, study names, etc.

If there isn't any safety limit or margin of safety, state "No PoD identified".

2) Lowest threshold section (format: narrative form)
If no safety limits are identified, determine whether the authors specify the lowest threshold or dose level among the reported NOAEL, LOAEL, LOEL, BMDL, or other dose values for use in risk assessment, or identify the lowest dose at which an adverse or toxic effect is observed. Then provide the following details:
- the value with units of the lowest dose level or threshold (e.g., NOAEL, LOAEL, LOEL, BMDL, dose level, etc) identified by the authors;
- the type of dose level or threshold identified (systemic, reproductive, developmental, maternal, etc.);
- the critical effect underlying the dose level or threshold;
- whether the dose level or threshold correspond to the highest dose tested;
- a short description of the supporting study (species, duration, exposure route) related to the lowest dose level or threshold identified by the authors;
- any discussion by the authors regarding any conflicting values or interpretations reported by different organizations or bodies on such lowest threshold or dose level. Mention whether the authors acknowledge alternative lowest thresholds proposed elsewhere.

as: NOAEL 300 mg/kg bw/d (ECHA) and NOAEL 1000 mg/kg bw/d (US EPA).<br>Then, add an **interpretative narrative summary** of the study (less than 200 words) exactly as taken from the document that describes how the authors explain the identification of the critical effects that drive threshold selection; whether the authors observe any dose-response evidence supporting those thresholds; whether the authors discuss any mechanistic insights or biological plausibility informing adversity or human relevance.<br>**- Reliability:** summarize (in less than 30 words) whether the study explicitly reports compliance with an OECD test guideline or other quality standards (if such information is not stated, provide an informed judgment on whether the study appears to resemble an OECD guideline design, and clearly identify and label this as "inferred judgment"); also specify whether the study was explicitly described in the document as "pre-GLP," "guideline-like," "for information only," "adequate," or "limited in reliability"; always make it explicit in each case whether the reliability designation was taken directly from the source text or inferred based on the reported design and methods.<br>- **Citations**: extract citations (e.g., primary sources, study names, external reports names) associated with the summarized evidence (e.g., (e.g., Finkelstein and Gold (1959), Jonker and Hollanders (1991)).<br><br>3) A "unique study" is defined by its primary experimental design (species, strain, study duration, purpose, and route).<br><br>**Output format:**<br>**Create the following table for all the identified studies:**<br>| **Identifier** | Source document details (title, authors, year) | Short Reference | **Study type** | **Experimental design** | **Findings** | **Interpretation| Reliability** | **Citations** | <br>Do not create any output if there is not any study identified; in such a case, just write "No toxicity studies (systemic, DART, carcinogenicity) identified"	Include relevant in-text citations provided in the documents to primary references, study names, etc.<br><br>If there isn't any threshold identified by the authors, state "No lowest threshold identified by the authors".<br><br>3**) Interpretation section (format: narrative form)**<br>Independently determine the lowest reported threshold value in the document (NOAEL, LOAEL, NOEL, LOEL, BMDL); if none of these is provided, identify the lowest dose at which an adverse or toxic effect is observed in the studies reported. Then provide the following details:<br>- the value with units of the lowest dose level or threshold (e.g., NOAEL, LOAEL, LOEL, BMDL, dose level, etc) identified by the authors;<br>- the type of dose level or threshold identified (systemic, reproductive, developmental, maternal, etc.);<br>- the critical effect underlying the dose level or threshold;<br>- whether the dose level or threshold correspond to the highest dose tested;<br>- a short description of the supporting study (species, duration, exposure route) related to the lowest dose level or threshold identified by the authors;<br><br>Report your selected lowest threshold or lowest dose level, even if the authors made a different choice in their evaluation.<br><br>Include relevant in-text citations provided in the documents to primary references, study names, etc.<br><br>If there are multiple lowest thresholds or dose levels from different studies, report both with the corresponding details.<br>If there isn't any lowest threshold or lowest dose, state: "No lowest threshold or lowest dose found".<br><br>4) **Developmental and reproductive toxicity section (format: narrative form)**<br>Generate a summary on the concern for reproductive and developmental toxicity of Tri-n-butyl acetyl citrate (ATBC, CAS RN 77-90-7) as stated by the authors and according to these rules:<br>- Report whether the authors report a conclusion on whether there is concern (or not) for reproductive and developmental toxicity;<br>- Summarize their reasoning behind the conclusion including their interpretation of the findings from key toxicity in vivo studies or other supporting evidence.<br>- For the key in vivo studies supporting the conclusions, report the study details including species, type of experiments, duration, route of exposure, critical effects and the doses and thresholds with units at which the relevant effects have been observed.<br>- Pinpoint any supporting studies (e.g., New Approach Methodologies, in vitro studies, in silico predictions, read-across, etc) mentioned to support the conclusions on reproductive and developmental toxicity;<br>- Explicitly report the conclusions of the authors about any concern for endocrine disruption and hormonal perturbation as well as endocrine activity in general.<br>- In addition, independently identify the lowest reported threshold for reproductive and developmental toxicity in the document (e.g., NOAEL/LOAEL/NOEL/LOEL/BMDL for

	reproductive, developmental, maternal), regardless of whether this has been identified already by the authors. Always specify whether the designation of the lowest threshold was taken directly from the source text or inferred based on the reported design and methods. For this lowest threshold, report the threshold value with units and type; the critical effect associated with it; the study from which it originates with the exposure route and duration (if provided).<br>- Include relevant in-text citations provided in the documents to primary references, study names, etc.<br>- If there isn't any reproductive or developmental toxicity evidence to summarize, state: "No DART thresholds to summarize".<br><br>5) **Endocrine activity (format: narrative form)**<br>Generate a summary on the concern for endocrine disruption potential and hormonal perturbation of Tri-n-butyl acetyl citrate (ATBC, CAS RN 77-90-7) as stated by the authors and according to these rules:<br>- Summarize any consideration and conclusions reported by the authors on endocrine disruption potential and hormonal perturbation or endocrine activity in general; summarize any conclusion made (concern/no concern) on endocrine disruption potential and hormonal perturbation and summarize the reasoning;<br>- Summarize their reasoning behind the conclusion including their interpretation of the findings from key toxicity in vivo studies () or other supporting evidence.<br>- For the key in vivo studies supporting the conclusions, report the study details including species, type of experiments, duration, route of exposure, critical effects and the doses and thresholds with units at which the relevant effects have been observed.<br>- Pinpoint any supporting studies (e.g., New Approach Methodologies, in vitro studies, in silico predictions, read-across, etc) mentioned to support the conclusions on endocrine activity, hormonal perturbation and also endocrine disruption potential;<br>- Summarize any mechanistic insights offered by the authors.<br>- Include relevant in-text citations provided in the documents to primary references, study names, etc.<br>- If there isn't any reproductive or developmental toxicity evidence to summarize, state: "No Endocrine activity to summarize".<br><br>**6) Carcinogenicity section (format: narrative form)**<br>Generate a summary on the "**carcinogenicity potential**" as stated by the authors:<br>- Report the conclusion of the authors on the carcinogenicity potential;<br>- Summarize the rationale behind these conclusions as discussed by the authors including available in vivo studies or human data and epidemiological data and historical data.<br>- Pinpoint any supporting studies (e.g., New Approach Methodologies, in vitro studies, in silico predictions, read-across, etc) mentioned to support the carcinogenicity conclusion;<br>- Also report whether the authors acknowledge any conflict with conclusions from other bodies;<br>- Include available reference made in the document to the IARC conclusions;<br>- Include relevant in-text citations provided in the documents to primary references, study names, etc;<br>- If there isn't any carcinogenicity evidence to summarize, state: "No carcinogenicity evidence to summarize".

	**Output format**<br>Create the following table: | Document unique identifier	Source document details (title, authors, year)	Short Reference	POD| Lowest Threshold	Interpretation	Reproductive and Developmental Toxicity	Endocrine activity and hormonal perturbation	Carcinogenicity | <br>If the document does not report any toxicity study with systemic effects, state clearly: "No evidence regarding systemic effects was identified in the document."

S3.3. Integrated analysis and summarization – Example of ChatGPT prompts

Prompt for Pharmacological/Biological Data

Summarization of Pharmacological/Biological Data using the extracted table created in the Information extraction step

You are a pharmacologist and a toxicologist with ERT and DABT certification. You must act as a neutral scientific summarizer, **EXTRACTING** only what is explicitly stated in the uploaded TEXT. You must never speculate, infer, or summarize the absence of data. You must comply with the following rules exactly.

TASK: Extract only from the uploaded TEXT (TABLE FORMAT) any data on pharmacological or biological activity of:
Acetyl Tributyl Citrate (ATBC; Tri-n-butyl acetyl citrate, CAS 77-90-7)
according to the following rules.

Strictly follow these **GENERAL RULES**:

- Use only information explicitly present in the PROVIDED TABLE. Do not infer missing data or introduce external context.
- If the TEXT contains no explicit mention of a relevant activity or finding, omit it entirely and do not state or imply that it was not found, tested, or reported.
- When citing evidence, refer only to the "Short reference" column as the source label including it in parenthesis (CIR, 2019). Do not reference the Identifier field.

Strictly follow these **INSTRUCTIONS**:

- Summarize (in fewer than 200 words) the findings, comments, observations, conclusions from the TEXT provided regarding these biological or pharmacological data:

- Endocrine or hormonal activity (e.g., estrogenic, androgenic, thyroid, or steroidogenic effects)
- Anticholinergic activity
- Other receptor or enzyme modulation relevant to toxicity or pharmacodynamics (specify receptor/enzyme if stated)
- Neurotoxic functional effects following interaction with given receptors
- Formation of reactive metabolites and in particular electrophilic species that can covalently bind to biological macromolecules

- Indicate whether these findings, conclusions, comments are regarded as positive, negative, or inconclusive (or equivocal).
- Mention only the endpoints or activities for which the TEXT provides a stated result or interpretation. You must not mention or paraphrase that the document did not report a given finding.
- The citations column report primary references that may indicate the same type of study or evidence summarized differently.
- Include citations of the summarized evidence using only the "Short reference" label: do not use the Identifier field.
- Clearly identify and compare findings across sources, noting agreement, differences, or conflicts exactly for the main effects as they appear, always name or cite each source (primary and secondary sources) as stated in the text (e.g., "CIR , 2019 reported…", "In contrast, Smith 1998 found…").
- Limit reinterpretation of data, providing a summary of the meaning as faithfully as possible to the text provided.

Output format:
- Title: "Biological and Pharmacological activity"

- Use a narrative style.

IMPORTANT NOTE: The next prompt will include the input, which is A TABLE with the following columns:
| Identifier | Source document details (title, authors, year) | Short Reference | Test Type | Biological Activity / Mechanism | Test System / Species | Observed Effect(s) & conclusions | Citations |

Prompt for Toxicokinetics

Summarization of Toxicokinetic Data using the extracted table created in the Information extraction step

Role: You are an expert on toxicokinetics and ADME (Absorption, Distribution, Metabolism, Excretion). You must act as a neutral scientific summarizer, **EXTRACTING** only what is explicitly stated in the uploaded TEXT. You must never speculate, infer, or summarize the absence of data. You must comply with the following rules exactly.

Task: Extract only from the uploaded TEXT (TABLE FORMAT) any data on toxicokinetics (or pharmacokinetics) and ADME (Absorption, Distribution, Metabolism, Excretion) of:
Acetyl Tributyl Citrate (ATBC; Tri-n-butyl acetyl citrate, CAS 77-90-7)
according to the following rules.

Strictly follow these **GENERAL RULES**:

- Use only information explicitly present in the PROVIDED TABLE. Do not infer missing data or introduce external context.
- If the TEXT contains no explicit mention of a relevant activity or finding, omit it entirely and do not state or imply that it was not found, tested, or reported.
- When citing evidence, refer only to the “Short reference” column as the source label, including it in parenthesis (CIR, 2019). Do not reference the Identifier field.

Strictly follow these **INSTRUCTIONS**:

- The goal is to summarize only information on **internal exposure and absorption, bioavailability, distribution, metabolism, or excretion** following exposure (e.g., oral, dermal, inhalation, parenteral, ocular).
- Exclude any studies limited to external exposure, migration, or leaching

1) Create a summary section about **Absorption** according to the following rules:
- Identify the Section type about ABSORPTION in the UPLOADED TABLE
- Summarize in less than 200 words the evidence about the absorption and bioavailability of the substance, reporting specific values of oral bioavailability or other related parameters, and highlighting any study mentioned to draw the conclusions. Report any information on dermal absorption, if any. If there is no evidence about absorption or bioavailability, then simply write "No evidence in the documents".
- Mention only the endpoints or activities for which the TEXT provides a stated result or interpretation. You must not mention or paraphrase that the document did not report a given finding.
- The citations column report primary references that may indicate the same type of study or evidence summarized differently.
- Include citations of the summarized evidence using only the “Short reference” label: do not use the Identifier field.
- Clearly identify and compare findings across sources, noting agreement, differences, or conflicts exactly for the main effects as they appear, always name or cite each source (primary and secondary sources) as stated in the text (e.g., “CIR , 2019 reported…”, “In contrast, Smith 1998 found…”).
- Limit reinterpretation of data, providing a summary of the meaning as faithfully as possible to the text provided.

2) Create a summary section about **Distribution** according to the following rules:
- Identify the Section type about **Distribution** in the UPLOADED TABLE
- Summarize in less than 100 words the conclusions of the document on the distribution of the substance highlighting whether the TEXT mentions any potential for bioaccumulation. Shortly summarize the studies used to draw conclusions on bioaccumulation, if any. If there is no evidence about distribution, then simply write "No evidence in the documents".
- Mention only the endpoints or activities for which the TEXT provides a stated result or interpretation. You must not mention or paraphrase that the document did not report a given finding.
- The citations column report primary references that may indicate the same type of study or evidence summarized differently.
- Include citations of the summarized evidence using only the “Short reference” label: do not use the Identifier field.

- Clearly identify and compare findings across sources, noting agreement, differences, or conflicts exactly for the main effects as they appear, always name or cite each source (primary and secondary sources) as stated in the text (e.g., “CIR , 2019 reported…”, “In contrast, Smith 1998 found…”).<br>- Limit reinterpretation of data, providing a summary of the meaning as faithfully as possible to the text provided.<br><br>3) Create a summary section about **Metabolism** according to the following rules:<br>- Identify the Section type about **Metabolism** in the UPLOADED TABLE<br>Summarize in less than 150 words the metabolism of the substance. Highlight any relevant metabolite mentioned and whether this is human relevant, if discussed. Report if any of these metabolites are considered to be of concern. If there is no evidence about metabolism, then simply write "No evidence in the documents".<br>- Mention only the endpoints or activities for which the TEXT provides a stated result or interpretation. You must not mention or paraphrase that the document did not report a given finding.<br>- The citations column report primary references that may indicate the same type of study or evidence summarized differently.<br>- Include citations of the summarized evidence using only the “Short reference” label: do not use the Identifier field.<br>- Clearly identify and compare findings across sources, noting agreement, differences, or conflicts exactly for the main effects as they appear, always name or cite each source (primary and secondary sources) as stated in the text (e.g., “CIR , 2019 reported…”, “In contrast, Smith 1998 found…”).<br>- Limit reinterpretation of data, providing a summary of the meaning as faithfully as possible to the text provided.<br><br>3) Create a summary section about **Excretion** according to the following rules:<br>- Identify the Section type about **Excretion** in the UPLOADED TABLE<br>- Summarize in less than 100 words the the excretion of the substance. If there is no evidence about excretion, then simply write "No evidence in the document".<br>- Mention only the endpoints or activities for which the TEXT provides a stated result or interpretation. You must not mention or paraphrase that the document did not report a given finding.<br>- The citations column report primary references that may indicate the same type of study or evidence summarized differently.<br>- Include citations of the summarized evidence using only the “Short reference” label: do not use the Identifier field.<br>- Clearly identify and compare findings across sources, noting agreement, differences, or conflicts exactly for the main effects as they appear, always name or cite each source (primary and secondary sources) as stated in the text (e.g., “CIR , 2019 reported…”, “In contrast, Smith 1998 found…”).<br>- Limit reinterpretation of data, providing a summary of the meaning as faithfully as possible to the text provided.<br><br>**Output format and content:**<br>- Title of the section: “Toxicokinetics”<br>- Create 4 subsection: 1) “Absorption”; 2) “Distribution”; 3) “Metabolism”; 4) “Excretion”.<br>- Use a narrative style<br><br>IMPORTANT NOTE: The next prompt will include the input, which is A TABLE with the following columns:<br>| Document unique identifier | Source document details (title, authors, year) | Short reference | Section type (Absorption or Distribution or Metabolism or Excretion) | Summary | Citations |

Prompt for Genotoxicity

**Summarization of genotoxicity studies using the extracted tables for in vivo and in vitro genotoxicity created in the Information extraction step**	**Summarization of genotoxicity conclusions using the extracted table for genotoxicity conclusions created in the Information extraction step**
**Role:** You are an experienced genetic toxicologist with expertise in evaluating in vitro genotoxicity and in vivo mutagenicity studies.<br><br>**Task:** Using only the uploaded TEXT (TABLE FORMAT), summarize any finding related to genotoxicity and mutagenicity of:<br>Acetyl Tributyl Citrate (ATBC; Tri-n-butyl acetyl citrate, CAS 77-90-7)	**Role:** You are an experienced genetic toxicologist with expertise in evaluating in vitro genotoxicity and in vivo mutagenicity studies.<br><br>**Task:** Using only the uploaded TEXT (TABLE FORMAT), summarize conclusions related to genotoxicity and mutagenicity of:<br>Acetyl Tributyl Citrate (ATBC; Tri-n-butyl acetyl citrate, CAS 77-90-7)

according to the following rules.<br><br>Strictly follow these **GENERAL RULES**:<br>• Use only information explicitly present in the PROVIDED TABLE. Do not infer missing data or introduce external context.<br>• If the TEXT contains no explicit mention of a relevant activity or finding, omit it entirely and do not state or imply that it was not found, tested, or reported.<br>• When citing evidence, refer only to the "Short reference" column as the source label, including it in parenthesis (CIR, 2019). Do not reference the "Identifier field".<br><br>Strictly follow these **INSTRUCTIONS**:<br>1) Generate a summary: summarize (in fewer than 200 words) the **findings on mutagenicity and genotoxicity according to the following rules:**<br>- Summarize the results (positive, negative, inconclusive) of the Ames test reporting the strains tested, and the metabolic activation (with or without S9). Note any existing limitations in the tests (e.g., reliability).<br>- Summarize the results of other in vitro tests (positive, negative, inconclusive) with a short description of the tests. Note any existing limitations in the tests (e.g., reliability).<br>- Summarize of the in vivo tests (positive, negative, inconclusive) with a short description of the tests. Note any existing limitations in the tests (e.g., reliability).<br>- Highlight any conflicting results between the in vitro evidence and the in vivo evidence or across the different studies;<br>- Mention only the endpoints or activities for which the TEXT provides a stated result or interpretation. You must not mention or paraphrase that the document did not report a given finding.<br>- The citations column report primary references that may indicate the same type of study or evidence summarized differently.<br>- Include citations of the summarized evidence using only the "Short reference" label; do not use the "Identifier" field.<br>- Clearly identify and compare findings across sources, noting agreement, differences, or conflicts exactly for the main effects as they appear, always name or cite each source (primary and secondary sources) as stated in the text (e.g., "CIR , 2019 reported…", "In contrast, Smith 1998 found…").<br>- Limit reinterpretation of data, providing a summary of the meaning as faithfully as possible to the text provided.<br><br>2) Generate a call: using the available data and your expert judgement, first draw a conclusion on mutagenicity (and in this respect mention the results for mutagenicity) and then draw an overall conclusion for genotoxicity. Explain your rationale (including considerations on in vivo and in vitro results or reliability). Explicitly state that the call is based on expert judgement using the information provided in the knowledge-base.<br><br>**Output format and content:**<br>- Title of the section: "Genotoxicity"<br>- Create 2 subsections:<br>1) "Summary"<br>2) "Expert judgement call"<br><br>- Use a narrative style<br><br>IMPORTANT NOTE: The next 2 prompts will include the input, which are 2 TABLES (one for in vitro studies and one for in vivo studies) with the following columns:	according to the following rules.<br><br>Strictly follow these **GENERAL RULES**:<br>• Use only information explicitly present in the PROVIDED TABLE. Do not infer missing data or introduce external context.<br>• If the TEXT contains no explicit mention of a relevant activity or finding, omit it entirely and do not state or imply that it was not found, tested, or reported.<br>• When citing evidence, refer only to the "Short reference" column as the source label, including it in parenthesis (CIR, 2019). Do not reference the "Identifier field".<br><br>Strictly follow these **INSTRUCTIONS**:<br>1) Summarize the calls for mutagenicity and genotoxicity across different documents.<br>- The purpose is only to report the final calls and to note whether there are conflicting calls across documents.<br>- Note whether there are some unique genotoxicity studies driving any different calls across documents.<br>- Include citations of the summarized evidence for a given document using only the "Short reference" label.<br><br>2) Generate an overall conclusion (50 words) regarding the consistency of the calls across documents and cite the documents using only the "Short reference" label.<br><br>**Output format and content:**<br>- Title of the section: "Genotoxicity calls"<br>- Use a narrative style<br><br>IMPORTANT NOTE: The next prompt will include the input, which is a TABLES with the following columns:<br>| Document unique identifier | Source document details (title, authors, year) | Short reference | Summary | Citations |

<table>
<tr><td>| Identifier | Source document details (title, authors, year) | Short Reference | Study type | Experimental design | Findings and interpretation | Reliability | Citations |</td><td></td></tr>
</table>

Prompt for Sensitization and Irritation

The prompts below address the sensitization potential; similar prompts can be used for the irritation potential.

<table>
<tr><th>Summarization of sensitization studies using the extracted table created in the Information extraction step</th><th>Summarization of sensitization conclusions using the extracted table created in the Information extraction step</th></tr>
<tr><td>Role: You are an experienced toxicologist with expertise in evaluating the sensitization potential of chemicals.

Task: Using only the uploaded TEXT (TABLE FORMAT), summarize any finding related to sensitization potential of:
Acetyl Tributyl Citrate (ATBC; Tri-n-butyl acetyl citrate, CAS 77-90-7)
according to the following rules.

Strictly follow these <u>GENERAL RULES</u>:
• Use only information explicitly present in the PROVIDED TABLE. Do not infer missing data or introduce external context.
• If the TEXT contains no explicit mention of a relevant activity or finding, omit it entirely and do not state or imply that it was not found, tested, or reported.
• When citing evidence, refer only to the “Short reference” column as the source label, including it in parenthesis (CIR, 2019). Do not reference the “Identifier field”.

Strictly follow these <u>INSTRUCTIONS</u>:
Generate a summary: summarize (in fewer than 200 words) the findings on sensitization potential (including allergy):
- Always specify the (potential) target organ when summarizing the results (e.g., skin/dermal, systemic).
- Summarize the results from in vitro studies (e.g., positive, negative, inconclusive, extreme sensitizer, strong sensitizer, weak sensitizer, sensitizer, etc) with a short description of the tests. Note any existing limitations in the tests (e.g., reliability).
- Summarize the results from in vivo studies (e.g., positive, negative, inconclusive, extreme sensitizer, strong sensitizer, weak sensitizer, sensitizer, etc) with a short description of the tests. Note any existing limitations in the tests (e.g., reliability).
- Reporting any threshold value such as EC3 related to the sensitization potential as stated in the document.
- Summarize any evidence from human data, epidemiological data or historic data studies (e.g., positive, negative, inconclusive, extreme sensitizer, strong sensitizer, weak sensitizer, sensitizer, etc) with a short description of this evidence. Note any existing limitations in the evidence (e.g., reliability).
- Summarize considerations from physico-chemical properties
- Highlight any conflicting results between available evidence;
- Mention only the endpoints or activities for which the TEXT provides a stated result or interpretation. You must not mention or paraphrase that the document did not report a given finding.
- The citations column report primary references that may indicate the same type of study or evidence summarized differently.
- Always include citations of the summarized evidence using only the “Short reference” label; do not use the “Identifier” field.
- Clearly identify and compare findings across sources, noting agreement, differences, or conflicts exactly for the main effects</td><td>Role: You are an experienced toxicologist with expertise in evaluating irritation and corrosion potential of chemicals.

Task: Using only the uploaded TEXT (TABLE FORMAT), summarize any finding related to sensitization potential of:
Acetyl Tributyl Citrate (ATBC; Tri-n-butyl acetyl citrate, CAS 77-90-7)
according to the following rules.

Strictly follow these <u>GENERAL RULES</u>:
• Use only information explicitly present in the PROVIDED TABLE. Do not infer missing data or introduce external context.
• If the TEXT contains no explicit mention of a relevant activity or finding, omit it entirely and do not state or imply that it was not found, tested, or reported.
• When citing evidence, refer only to the “Short reference” column as the source label, including it in parenthesis (CIR, 2019). Do not reference the “Identifier field”.

Strictly follow these <u>INSTRUCTIONS</u>:
1) Summarize the calls for the sensitization potential (including allergy) across different documents.
- The purpose is only to report the final calls and to note whether there are conflicting calls across documents.
- Always specify the (potential) target organ when summarizing the results (e.g., skin, systemic).
- Note whether there is some unique evidence driving any different calls across documents.
- Include citations of the summarized evidence for a given document using only the “Short reference” label.

2) Generate an overall conclusion (50 words) regarding the consistency of the calls across documents and cite the documents using only the “Short reference” label.

Output format and content:
- Title of the section: “Sensitization calls”
- Use a narrative style

IMPORTANT NOTE: The next prompt will include the input, which is a TABLES with the following columns:
| Document unique identifier | Source document details (title, authors, year) | Short reference| Summary | Citations</td></tr>
</table>

as they appear, always name or cite each source (primary and secondary sources) as stated in the text (e.g., “CIR , 2019 reported…”, “In contrast, Smith 1998 found…”).<br>- Limit reinterpretation of data, providing a summary of the meaning as faithfully as possible to the text provided.<br><br>2) Generate a call: using the available data and your expert judgement, first draw a conclusion on the sensitization potential and specify whether there are indications to classify the chemical as: sensitizer or non sensitizer (e.g, skin, systemic). Explain your rationale (including considerations on in vivo and in vitro results or reliability). Explicitly state that the call is based on expert judgement using the information provided in the knowledge-base.<br><br>**Output format and content:**<br>- Title of the section: “Sensitization”<br>- Create 2 subsections:<br>1) “Summary”<br>2) “Expert judgement call”<br>- Use a narrative style<br><br>IMPORTANT NOTE: The next prompt will include the input, which is a TABLE with the following columns: | Identifier	Source document details (title, authors, year)	Short Reference	Study type	Experimental design	Findings and interpretation	Reliability	Citations		

Prompt for Key toxicological data (systemic toxicity, DART, carcinogenicity)

**Summarization of reproductive and developmental toxicity from the extracted conclusions from the Information extraction step**	**Summarization of carcinogenicity from the extracted conclusions from the Information extraction step**	**Summarization of point of departure from the extracted conclusions from the Information extraction step**
Role: You are an experienced toxicologist with ERT and DABT certification. You must act as a neutral scientific summarizer, **EXTRACTING** only what is explicitly stated in the uploaded TEXT. You must never speculate, infer, or summarize the absence of data. You must comply with the following rules exactly.<br><br>**Task:** Using only the TEXT PROVIDED (TABLE FORMAT), summarize the conclusions on reproductive and developmental toxicity for:<br>Acetyl Tributyl Citrate (ATBC; Tri-n-butyl acetyl citrate, CAS 77-90-7)<br>according to the following rules.<br><br>Strictly follow these **<u>GENERAL RULES</u>**:<br>• Use only information explicitly present in the PROVIDED TABLE. Do not infer missing data or introduce external context.<br>• If the TEXT contains no explicit mention of a relevant activity or finding, omit it	Role: You are an experienced toxicologist with ERT and DABT certification. You must act as a neutral scientific summarizer, **EXTRACTING** only what is explicitly stated in the uploaded TEXT. You must never speculate, infer, or summarize the absence of data. You must comply with the following rules exactly.<br><br>**Task:** Using only the TEXT PROVIDED (TABLE FORMAT), summarize the conclusions on carcinogenicity potential for:<br>Acetyl Tributyl Citrate (ATBC; Tri-n-butyl acetyl citrate, CAS 77-90-7)<br>according to the following rules.<br><br>Strictly follow these **<u>GENERAL RULES</u>**:<br>• Use only information explicitly present in the PROVIDED TABLE. Do not infer missing data or introduce external context.<br>• If the TEXT contains no explicit mention of a relevant activity or finding, omit it entirely and do not state or imply that it was not found, tested, or reported.	Role: You are an experienced toxicologist with ERT and DABT certification. You must act as a neutral scientific summarizer, **EXTRACTING** only what is explicitly stated in the uploaded TEXT. You must never speculate, infer, or summarize the absence of data. You must comply with the following rules exactly.<br><br>**Task:** Using only the TEXT PROVIDED (TABLE FORMAT), extract information on toxicity thresholds and dose levels for:<br>Acetyl Tributyl Citrate (ATBC; Tri-n-butyl acetyl citrate, CAS 77-90-7)<br>according to the following rules.<br><br>Strictly follow these **<u>GENERAL RULES</u>**:<br>• Use only information explicitly present in the PROVIDED TABLE. Do not infer missing data or introduce external context.

entirely and do not state or imply that it was not found, tested, or reported.<br>• When citing evidence, refer only to the "Short reference" column as the source label, including it in parenthesis (CIR, 2019). Do not reference the Identifier field.<br><br>Strictly follow these **<u>INSTRUCTIONS</u>**:<br><br>• The goal is to summarize only conclusions on **reproductive and developmental toxicity** following exposure (e.g., oral, dermal, inhalation, parenteral, ocular).<br><br>• Create a summary section (in less than 200 words) about **reproductive and developmental toxicity** for Acetyl Tributyl Citrate (ATBC; Tri-n-butyl acetyl citrate, CAS 77-90-7) according to the following rules:<br>- Identify the section about **reproductive and developmental toxicity** in the UPLOADED TABLE ("Reproductive and Developmental Toxicity" column)<br>- Summarize whether there is a consensus conclusion across the listed documents for **reproductive and developmental toxicity potential** (e.g., no concern, concern, equivocal concern across documents).<br>- Explain the consensus rationale across documents about the conclusion on **reproductive and developmental toxicity**.<br>- Highlight the key in vivo studies cited to support the conclusion including details on the threshold (NOAEL/LOAEL for reproductive/developmental), its value and units, the species tested, exposure route, the duration and the critical effect.<br>- Identify the lowest threshold across documents for reproductive and developmental toxicity and report the details of the corresponding study and citations. Mention all the documents supporting the same findings.<br>- Summarize any mechanistic insights provided in the documents.<br>- If the substance is data-poor, pinpoint any supporting evidence used by the documents to strengthen the conclusions (e.g., New Approach Methodologies, in vitro studies, in silico predictions, read-across, etc).<br>- Also report whether the authors acknowledge any conflict with conclusions from other bodies;<br>- Mention only the endpoints or activities for which the TEXT provides a stated result or interpretation. You must not mention or paraphrase that the document did not report a given finding.	• When citing evidence, refer only to the "Short reference" column as the source label, including it in parenthesis (CIR, 2019). Do not reference the Identifier field.<br><br>Strictly follow these **<u>INSTRUCTIONS</u>**:<br><br>• The goal is to summarize only conclusions on **carcinogenicity potential** following exposure (e.g., oral, dermal, inhalation, parenteral, ocular).<br><br>• Create a summary section (in less than 200 words) about **carcinogenicity** for Acetyl Tributyl Citrate (ATBC; Tri-n-butyl acetyl citrate, CAS 77-90-7) according to the following rules:<br>- Identify the section about **carcinogenicity** in the UPLOADED TABLE ("Carcinogenicity" column)<br>- Summarize whether there is a consensus conclusion across the listed documents for **carcinogenicity potential** (e.g., no concern, concern, equivocal concern across documents).<br>- Explain the consensus rationale across documents about the conclusion on **carcinogenicity.**<br>- Highlight the key in vivo studies cited to support the conclusion including details on doses (value and unit), the species tested, the duration, route and the critical effect.<br>- Summarize any mechanistic insights provided in the documents (e.g., genotoxic mechanism of carcinogenicity, non-genotoxic mechanism, binding to receptor).<br>- Pinpoint any supporting studies (e.g., New Approach Methodologies, in vitro studies, in silico predictions, read-across, etc) mentioned to support the carcinogenicity conclusion;<br>- Also report whether the authors acknowledge any conflict with conclusions from other bodies;<br>- Only if explicitly mentioned, include available reference made in the document to the IARC conclusions;<br>- Include relevant in-text citations to the documents using the "Short reference" label;<br>- Include citations of the summarized evidence using only the "Short reference" label: do not use the Identifier field.<br>- Clearly identify and compare findings across documents, noting agreement, differences, or conflicts exactly for the main effects as they appear, always name or cite each source (primary and secondary sources) as stated in the text (e.g., "CIR , 2019 reported…", "In contrast, Smith 1998 found…").<br>- Limit reinterpretation of data, providing a summary of the meaning as faithfully as possible to the text provided.<br>- If there isn't any carcinogenicity evidence to summarize, state: "No carcinogenicity evidence to summarize".<br><br>**Output format and content:**	• If the TEXT contains no explicit mention of a relevant activity or finding, omit it entirely and do not state or imply that it was not found, tested, or reported.<br>• When citing evidence, refer only to the "Short reference" column as the source label, including it in parenthesis (CIR, 2019). Do not reference the Identifier field.<br><br>Strictly follow these **<u>INSTRUCTIONS</u>**:<br><br>1) Identify the section named PoD in the UPLOADED TABLE, and create a summary section following these rules:<br>- Only using the information included in the "PoD" column, list the point of departures and safety limits reported for all the documents.<br>- Include any summarized evidence with the corresponding citations using the corresponding "Short reference" label.<br>- Omit the information if there is nothing to report and write "Nothing to report".<br><br>2) Identify the section named "Lowest Threshold" in the UPLOADED TABLE, and create a summary section following these rules:<br>- Only using the information included in the "Lowest Threshold" column,<br>summarize the lowest thresholds identified in the documents and report the short description of the supporting study (species, duration, exposure route).<br>- Include any summarized evidence with the corresponding citations using the corresponding "Short reference" label.<br>- Omit the information if there is nothing to report and write "Nothing to report".<br><br>2) Identify the section named "Interpretation" in the UPLOADED TABLE and create a summary section following these rules:<br>- Only using the information included in the "Interpretation" column, summarize the lowest thresholds identified in the documents and report the short description of the supporting study (species, duration, exposure route).<br>- Include any summarized evidence with the corresponding citations

- Include citations of the summarized evidence using only the “Short reference” label: do not use the Identifier field.<br>- Clearly identify and compare findings across documents, noting agreement, differences, or conflicts exactly for the main effects as they appear, always name or cite each source (primary and secondary sources) as stated in the text (e.g., “CIR , 2019 reported…”, “In contrast, Smith 1998 found…”).<br>- Limit reinterpretation of data, providing a summary of the meaning as faithfully as possible to the text provided.<br>- If there isn’t any reproductive or developmental toxicity evidence to summarize, state: “No reproductive or reproductive or developmental toxicity evidence to summarize”.<br><br>**Output format and content:**<br>- Title of the section: “Reproductive and developmental toxicity”<br>- Use a narrative style<br><br>IMPORTANT NOTE: The next prompt will include the input, which is A TABLE with the following columns:<br><br>| Document unique identifier | Source document details (title, authors, year) | Short Reference | POD| Lowest Threshold | Interpretation | Reproductive and Developmental Toxicity | Endocrine activity and hormonal perturbation | Carcinogenicity |	- Title of the section: “Carcinogenicity”<br>- Use a narrative style<br><br>IMPORTANT NOTE: The next prompt will include the input, which is A TABLE with the following columns:<br><br>| Document unique identifier | Source document details (title, authors, year) | Short Reference | POD| Lowest Threshold | Interpretation | Reproductive and Developmental Toxicity | Endocrine activity and hormonal perturbation | Carcinogenicity |	using the corresponding “Short reference” label.<br>- Omit the information if there is nothing to report and write “Nothing to report”.<br><br>**Output format and content:**<br><br>Create a table:<br><br>| PoD summary | Lowest threshold summary | Interpretation summary | <br>IMPORTANT NOTE: The next prompt will include the input, which is A TABLE with the following columns:<br><br>| Document unique identifier | Source document details (title, authors, year) | Short Reference | POD| Lowest Threshold | Interpretation | Reproductive and Developmental Toxicity | Endocrine activity and hormonal perturbation | Carcinogenicity |

Section S4: Acetyl Tributyl Citrate (CAS 77-90-7) – supporting evidence

S.4.1. Data search

The following table highlights the data sources identified during the data discovery phase, where LLM-assisted exploration was complemented by targeted manual literature searches.

Source document details	Short Reference	Type of reference
U.S. Food and Drug Administration. (2025, September 29). *21 CFR 172.515 – Synthetic Flavoring Substances and Adjuvants*	(21 CFR 172.515, 2025)	This regulation lists synthetic flavoring substances and adjuvants permitted for direct addition to food. It specifies conditions for safe use, compliance with good manufacturing practices, and concentration limits. The rule ensures consumer safety while allowing specific synthetic compounds in food flavoring.

Johnson (2023), "Acetyl Trialkyl Citrates," International Journal of Toxicology, Vol. 42(Suppl. 3), 7S–9S. DOI: 10.1177/10915818231204615.	(Johnson et al., 2023)	The Cosmetic Ingredient Review (CIR) Expert Panel reaffirmed that Acetyl Triethyl Citrate, Acetyl Tributyl Citrate, Acetyl Triethylhexyl Citrate, and Acetyl Trihexyl Citrate are safe for cosmetic use. Updated data showed increased use but no new safety concerns, confirming these ingredients remain safe at current concentrations.
Cosmetic Ingredient Review Expert Panel. (2019). *Johnson, W. Jr Safety Assessment of Acetyl Trialkyl Citrates as Used in Cosmetics. Cosmetic Ingredient Review Expert Panel Re-Review, Washington, DC.*	(CIR, 2019)	The review reaffirms that Acetyl Tributyl Citrate is safe for use in cosmetics at concentrations up to ~8.9%. Animal and in vitro studies showed low toxicity, no carcinogenicity, and no genotoxic effects. Mild adaptive liver changes were observed at high doses, but overall systemic and reproductive toxicity were minimal.
Cosmetic Ingredient Review Expert Panel. (2002). *Johnson, W. Jr. Final Report on the Safety Assessment of Acetyl Triethyl Citrate, Acetyl Tributyl Citrate, Acetyl Trihexyl Citrate, and Acetyl Trioctyl Citrate., Journal of the American College of Toxicology / Cosmetic Ingredient Review Expert Panel Report, Washington, DC..*	(CIR, 2002)	Acetyl Tributyl Citrate showed low acute and chronic toxicity, with no carcinogenicity, genotoxicity, or reproductive toxicity in animal studies. Human patch tests found it non-irritating and non-sensitizing. Approximately 99% is excreted unchanged. The CIR Expert Panel concluded it is safe as used in cosmetics, typically up to 7%.
EFSA Panel on Food Contact Materials, Enzymes, Flavourings and Processing Aids (2012), "Scientific Opinion on Flavouring Group Evaluation 10, Revision 3 (FGE.10Rev3): Aliphatic primary and secondary saturated and unsaturated alcohols, aldehydes, acetals, carboxylic acids and esters containing an additional oxygenated functional group and lactones from chemical groups 9, 13 and 30." *EFSA Journal* 2012;10(3):2563.	(EFSA, 2012)	Acetyl Tributyl Citrate (FL-no: 09.511, CAS 77-90-7) was evaluated as a supporting substance. JECFA specification confirmed (2000d). Acute oral LD_{50} in rats >30 g/kg bw (Finkelstein & Gold, 1959). Subchronic dietary studies up to 5000 mg/kg bw/day showed no adverse effects. EFSA concluded "no safety concern."
EFSA (2005), *A 10th list of substances for food contact materials. The EFSA Journal* (2005) 273, 19–22.	(EFSA, 2005)	Acetyl tributyl citrate (ATBC, Ref. No. 93760, CAS 77-90-7) is used as a plasticizer in food-contact PVC/PVDC films. Migration studies (9–28 mg/kg) showed low migration. Toxicological studies revealed no genotoxicity, carcinogenicity, or accumulation. NOAEL = 100 mg/kg bw/day; TDI = 1 mg/kg bw. Classified SCF List 2 (safe for use).
U.S. Environmental Protection Agency (2014), "Screening-Level Hazard Characterization: Acetyl Tributyl Citrate (CASRN 77-90-7)", *Hazard Characterization Document*, Office of Pollution Prevention and Toxics, EPA, Washington, DC.	(EPA, 2014)	EPA's 2014 hazard characterization found Acetyl Tributyl Citrate has low toxicity and low environmental hazard. It is readily biodegradable, with low persistence and bioaccumulation. In rats, systemic NOAEL ≈100–300 mg/kg bw/day; no mutagenicity observed. Acute aquatic toxicity was moderate (LC_{50} ≈ 38–60 mg/L for fish, EC_{50} ≈ 7.8 mg/L for invertebrates).
University of Cincinnati (2018), "Toxicity Review for Acetyl Tri-n-Butyl Citrate (ATBC)", prepared for the U.S. Consumer Product Safety Commission (CPSC), *University of Cincinnati, Department of Environmental Health, Risk Science Center*	(CPSC/University of Cincinnati, 2018)	Comprehensive toxicology review on ATBC. It reports low acute and repeated-dose toxicity, non-carcinogenicity in rats, no reproductive or developmental toxicity up to 1000 mg/kg-day, and rapid metabolism with low bioaccumulation potential. Liver weight increases were adaptive. Overall, ATBC shows low human health hazard at expected exposure levels.
CSTEE (2004), "Opinion on the Risk Assessment for Acetyl Tributyl Citrate (ATBC) Plasticizer Used in Children's Toys", *European Commission, Scientific Committee on Toxicity, Ecotoxicity and the Environment (CSTEE)*	(CSTEE, 2004)	The CSTEE concluded that ATBC-plasticized PVC toys pose no safety concern for children. Human saliva studies showed low migration rates (max 10.1 µg/min). A 13-week rat study with in utero exposure found a NOAEL of 100–300 mg/kg/day. The calculated Margin of Safety (MOS) was ~440, indicating safe use in mouthing toys.
FDA (2004), *Hydrolysis Studies of Acetyl Tributyl Citrate – Federal Register Notice (04-21587)*, U.S. Food and Drug Administration.	(FDA, 2004)	The FDA review concluded that Acetyl Tributyl Citrate (ATBC) hydrolyzes readily to tributyl citrate and acetic acid under simulated physiological conditions, indicating low persistence and low toxicity. Data from hydrolysis and metabolism tables supported its safe use as a plasticizer in food contact materials.

frontiers

ANSES (Agence nationale de sécurité sanitaire de l'alimentation, de l'environnement et du travail). (2016, March). *Analysis of the Most Appropriate Risk Management Option (RMOA) for Tributyl O-acetylcitrate (ATBC)*. French Competent Authority Report.	(ANSES, 2016)	The French RMOA concluded that ATBC shows low toxicity, no mutagenic or carcinogenic effects, and no evidence of endocrine disruption. It is readily metabolized and largely excreted. Environmental persistence is moderate to low, and ATBC is not classified as PBT/vPvB. Overall, it poses low risk to human health and the environment.
ECHA database	(ECHA 2025)	ECHA dossier including several toxicity studies.
Kim et al (2018). Pharmacokinetic Properties of Acetyl Tributyl Citrate, a Pharmaceutical Excipient. Pharmaceutics, 10(177), 1–10. https://doi.org/10.3390/pharmaceutics10040177	(Kim et al., 2018)	This study characterized ATBC's pharmacokinetics in rats using LC–MS/MS. ATBC was rapidly absorbed (Tmax = 0.4 h) and cleared (t½ = 0.58 h) with oral bioavailability of 27.4%. It was hydrolyzed quickly in rat plasma but stable in human plasma, metabolized mainly by CYP2C19 and CYP3A4. Findings support its low persistence and safety.
Jo et al (2024). Suspect Screening Candidate Exposure Biomarkers of Acetyl Tributyl Citrate and Acetyl Triethyl Citrate after Human Oral Administration. Environment International, 193, 109062. https://doi.org/10.1016/j.envint.2024.109062	(Jo et al., 2024)	The first controlled human exposure study of ATBC identified six urinary metabolites. ADBC-d3 and OH-ADBC-d3 were proposed as reliable biomarkers due to high detection and persistence. Parent ATBC was not found in urine, confirming rapid metabolism. Elimination half-lives ranged 1–10 h, indicating fast clearance and low bioaccumulation potential.
Rasmussen, L. M. (2017), *Effects of oral exposure to the phthalate substitute acetyl tributyl citrate on female reproduction in mice*, *Journal of Applied Toxicology*, 37(6):668–675..	(Rasmussen et al., 2017)	Female mice exposed orally to 5–10 mg/kg/day ATBC for 15 days showed no effects on fertility, estrous cyclicity, or gestation. However, 10 mg/kg/day reduced ovarian primordial, primary, and secondary follicle numbers, indicating potential ovarian toxicity at low doses, warranting further investigation of endocrine-disrupting effects.
Zhang, Y. (2023), "Acetyl Tributyl Citrate Exposure at Seemingly Safe Concentrations Induces Adverse Effects in Different Genders of Type 2 Diabetes Mice, Especially Brain Tissue." Toxics, 11(10), 877. DOI: 10.3390/toxics11100877	(Zhang et al., 2023)	The study found that even "safe" levels of acetyl tributyl citrate (ATBC) aggravated glucose–lipid metabolism disorders, caused cognitive deficits, oxidative stress, inflammation, and brain damage in type 2 diabetic mice, especially males. It concludes that ATBC may pose neurological risks to sensitive populations such as diabetics.

S.4.2. Information extraction

In accordance with the ICH Q3 guidance, the following types of information can be extracted from the selected knowledge base identified during the data discovery phase, using example prompts such as those presented in Section S4. An example of the extracted evidence is shown below for Toxicokinetics.

Short reference	Section type (Absorption or Distribution or Metabolism or Excretion)	Summary
CIR, 2019	Metabolism	Acetyl Tributyl Citrate activates the steroid and xenobiotic receptor (SXR) in the intestine, inducing CYP3A1 expression in rat intestinal tissues, but not in the liver. No human-relevant metabolites are mentioned as concerning.
CIR, 2019	Excretion	No evidence in the document
University of Cincinnati, 2018	Absorption	The document reports that ATBC is readily absorbed after oral administration. Following ingestion, ATBC is hydrolyzed in the gastrointestinal tract, leading to systemic exposure to its metabolites. Limited information is given on quantitative oral bioavailability. The review cites animal and in vitro hydrolysis studies demonstrating cleavage to tributyl citrate (TBC) and n-butanol, suggesting efficient gastrointestinal absorption of both the parent compound and metabolites. No explicit information is provided on dermal absorption.
University of Cincinnati, 2018	Distribution	The document states that ATBC and its metabolites show low potential for bioaccumulation. The parent compound and metabolites are widely distributed but rapidly cleared, as supported by findings of no tissue retention in animal studies.

University of Cincinnati, 2018	Metabolism	The document summarizes that ATBC undergoes extensive hydrolysis by esterases to yield tributyl citrate (TBC) and n-butanol. Further metabolism involves oxidation of butyl side chains and β-oxidation of citrate esters. ATBC and its metabolites are metabolized primarily in the liver via ester hydrolysis and oxidation, producing water-soluble metabolites that are readily excreted. The document notes no reactive or toxic intermediates. These metabolic transformations were reported to occur in both humans and animals and are consistent with rapid clearance.
University of Cincinnati, 2018	Excretion	The document reports that ATBC and its metabolites are rapidly excreted, mainly via urine and expired air as carbon dioxide. Fecal elimination is minimal. Excretion studies indicate almost complete clearance within 24 hours post-administration.
EFSA, 2005	Absorption	The EFSA report indicates that acetyl tributyl citrate (ATBC; tri-n-butyl acetyl citrate, CAS 77-90-7) is rapidly hydrolysed in the gastrointestinal tract to tributyl citrate and acetic acid, followed by further metabolism. The hydrolysis suggests that systemic absorption occurs primarily as metabolites rather than the parent compound. No quantitative absorption or bioavailability data (e.g., % absorbed) were provided.
EFSA, 2005	Distribution	The EFSA document notes that following metabolism, the resulting citrate esters are expected to enter normal metabolic pathways and are not considered bioaccumulative. No tissue distribution studies or quantitative data were included.
EFSA, 2005	Metabolism	The EFSA report explains that ATBC is enzymatically hydrolysed to tributyl citrate and acetic acid, which are then metabolized via normal citric acid cycle pathways. No human-specific metabolic differences or toxic metabolites were described. The metabolic pattern was interpreted as similar to other citrate esters and not of toxicological concern.
EFSA, 2005	Excretion	The EFSA document indicates that the metabolites of ATBC are excreted as carbon dioxide and water, consistent with normal metabolic processing of citrate esters. No quantitative excretion data were provided.
ANSES, 2016	Absorption	The document reports that ATBC is well absorbed via the oral route in rats and humans. Rapid hydrolysis occurs in the gastrointestinal tract and liver. The extent of oral absorption was estimated as high (>80%) based on available experimental evidence and read-across data from citrate esters. Dermal absorption is considered limited, as indicated by the physicochemical properties of ATBC and supporting data from in vivo and in vitro tests (CSTEE, 1999; US EPA, 2003).
ANSES, 2016	Distribution	ATBC and its metabolites are described as widely distributed following absorption. The document states that no evidence indicates bioaccumulation, given the substance's rapid metabolism and elimination.
ANSES, 2016	Metabolism	ANSES (2016) reports that ATBC is rapidly hydrolyzed to citric acid and n-butanol by esterases in the gastrointestinal tract and liver. Secondary metabolites include tributyl citrate and acetylated citric derivatives, which further undergo oxidation and conjugation. Takeshita et al. (2011) found that ATBC activated the pregnane X receptor (PXR/SXR) and induced intestinal CYP3A expression, suggesting that it can influence xenobiotic metabolism but not in an adverse manner. No metabolites were identified as toxicologically concerning.
ANSES, 2016	Excretion	The document states that ATBC and its metabolites are rapidly excreted, primarily via urine and feces within 48 hours post-administration, with negligible tissue retention.
Kim *et al.*, 2018	Absorption	In rats, Acetyl Tributyl Citrate (ATBC; CAS 77-90-7) was rapidly absorbed following oral administration (500 mg/kg) with a Tmax of 0.4 h. Oral bioavailability was reported as 27.4%. Plasma concentration profiles indicated nonlinear elimination at high doses. No dermal absorption studies were mentioned.
Kim *et al.*, 2018	Distribution	Intravenous plasma concentration–time data indicated that ATBC follows a two-compartment model, suggesting distribution to certain tissues at a slower rate. No bioaccumulation potential was noted.
Kim *et al.*, 2018	Metabolism	ATBC was rapidly metabolized in rat plasma (half-life ≈ 0.2 h) but stable in human plasma (> 80% remaining after 1 h). It was easily metabolized in both rat and human liver microsomes (half-life ≈ 0.26–0.64 h). CYP2C19 and CYP3A4 showed high metabolic activity for ATBC. Reported metabolites include acetyl citrate, monobutyl citrate, acetyl monobutyl citrate, dibutyl citrate, and acetyl dibutyl citrate, primarily formed via hydrolysis.
Kim *et al.*, 2018	Excretion	Approximately 99% of 14C-labeled ATBC administered orally to rats was excreted within 48 h via urine (59–70%), feces (25–36%), and expired air (2%).

S.4.3. Integrated analysis and summarization

The extracted evidence can be summarized and structured following the ICH Q3E template. The summarization process uses data extracted from the knowledge base in the previous step, guided by example prompts provided in Section S3. Illustrative examples of the resulting summaries are shown below.

Pharmacological/Biological Data – summarized evidence (example)

CIR (2019) reported that Acetyl Tributyl Citrate (ATBC) increased corticosteroid, corticosterone, aldosterone, and 11-deoxycorticosterone synthesis in H295R adrenal cells at 10 mM (Strajhar et al., 2017), indicating positive steroidogenic activity. The same source described anti-estrogenic and anti-androgenic activity in human cell lines, with no concurrent agonist activity (Park et al., 2019). CIR (2019) also noted activation of the steroid and xenobiotic receptor (SXR), resulting in CYP3A1 induction in intestinal but not hepatic tissue (Takeshita et al., 2011), confirming positive receptor-mediated enzyme induction.

The University of Cincinnati (2018) confirmed increased CYP11B2 and CYP21A2 expression and raised corticosterone, aldosterone, and progesterone in H295R cells (Strajhar et al., 2017), supporting positive steroidogenic enzyme modulation. The same review noted SXR activation with induction of related target genes (Takeshita et al., 2011) and absence of estrogenic or androgenic receptor binding in rat assays (Ohta et al., 2003, as cited by SCENIHR, 2016).

ANSES (2016) reported absence of estrogenic or androgenic receptor activity in vivo (Ohta et al., 2003) and confirmed pregnane X receptor (PXR/SXR) activation with intestinal CYP3A induction (Takeshita et al., 2011).

Rasmussen et al. (2017) observed reduced ovarian follicles in exposed mice, interpreted as potential ovarian endocrine disruption, and cited Takeshita et al. (2011) confirming SXR/PXR-mediated CYP3A induction.

Zhang et al. (2023) demonstrated positive neurotoxic functional effects in diabetic mice: ATBC reduced acetylcholine and serotonin levels, increased β-amyloid and phosphorylated tau, induced oxidative stress (ROS, MDA, 4-HNE), decreased antioxidant enzymes (SOD, GSH), and triggered neuroinflammatory cytokine release and glial activation.

Overall, across cited sources, ATBC shows potentially positive steroidogenic, anti-estrogenic, anti-androgenic, SXR/PXR activation, oxidative, and neuroinflammatory activities.

Toxicokinetics – summarized evidence (example)

Absorption

Across multiple sources, Acetyl Tributyl Citrate (ATBC; CAS 77-90-7) is consistently reported as rapidly absorbed following oral exposure. Kim et al. (2018) described fast absorption in rats (Tmax = 0.4 h) and oral bioavailability of 27.4%. EFSA (2005) stated that ATBC undergoes rapid gastrointestinal hydrolysis to tributyl citrate and acetic acid, implying systemic absorption mainly as metabolites. University of Cincinnati (2018) confirmed efficient oral absorption with hydrolysis in the gastrointestinal tract producing tributyl citrate and *n*-butanol. ANSES (2016) estimated high oral absorption (>80%) in rats and humans and noted limited dermal uptake based on physicochemical data and supporting in vivo/in vitro tests.

Citations: Kim et al., 2018; EFSA, 2005; University of Cincinnati, 2018; ANSES, 2016; Finkelstein and Gold (1959); Versar (2010); U.S. EPA (2008); ECHA (2018); CSTEE (1999); U.S. EPA (2003); Gäfvert et al. (1998)

Distribution

Kim et al. (2018) reported that intravenous plasma concentration–time data follow a two-compartment model, suggesting tissue distribution without bioaccumulation. University of Cincinnati (2018) and ANSES (2016) noted that ATBC and its metabolites are widely distributed but rapidly cleared, with no tissue retention. EFSA (2005) similarly indicated that metabolites enter normal metabolic pathways and are not bioaccumulative.

Citations: Kim et al., 2018; University of Cincinnati, 2018; ANSES, 2016; EFSA, 2005; Versar (2010); U.S. EPA (2008); ECHA (2018); CSTEE (1999); U.S. EPA (2003)

Metabolism

ATBC is consistently described as rapidly and extensively metabolized. Kim et al. (2018) found rapid hydrolysis in rat plasma ($t_{1/2} \approx 0.2$ h) and stability in human plasma (>80% remaining after 1 h), with metabolism in both rat and human liver microsomes ($t_{1/2} \approx 0.26$–0.64 h). Metabolites identified include acetyl citrate, monobutyl citrate, acetyl monobutyl citrate, dibutyl citrate, and acetyl dibutyl citrate, primarily from hydrolysis. University of Cincinnati (2018) described extensive esterase hydrolysis to tributyl citrate and *n*-butanol, followed by oxidation of butyl chains and β-oxidation of citrate esters, primarily in the liver. EFSA (2005) and ANSES (2016) both stated that ATBC is hydrolyzed to citric acid derivatives and *n*-butanol, metabolized through normal citric acid cycle pathways. Takeshita et al. (2011), cited by ANSES, reported intestinal activation of the pregnane X receptor and induction of CYP3A expression. No toxic metabolites were identified.

Citations: Kim et al., 2018; University of Cincinnati, 2018; EFSA, 2005; ANSES, 2016; Takeshita et al., 2011; Finkelstein and Gold (1959); Versar (2010); U.S. EPA (2008); ECHA (2018); CSTEE (1999); Gäfvert et al. (1998)

Excretion

Kim et al. (2018) reported that approximately 99% of orally administered ^14C-labeled ATBC was eliminated within 48 h, mainly via urine (59–70%), feces (25–36%), and expired air (2%). University of Cincinnati (2018) found rapid excretion of ATBC and metabolites, primarily in urine and as carbon dioxide in expired air, with nearly complete clearance within 24 h. ANSES (2016) described urinary and fecal elimination within 48 h and negligible tissue retention. EFSA (2005) indicated excretion of

metabolites as carbon dioxide and water.
Citations: Kim et al., 2018; University of Cincinnati, 2018; ANSES, 2016; EFSA, 2005; Johnson (2002); Finkelstein and Gold (1959); Versar (2010); U.S. EPA (2008); CSTEE (1999); U.S. EPA (2003)

Genotoxicity – summarized evidence (example)

Genotoxicity calls from documents
Across all reviewed documents, Acetyl Tributyl Citrate (ATBC; CAS 77-90-7) consistently demonstrated no genotoxic or mutagenic activity in both bacterial and mammalian test systems.
CIR (2019) reported uniformly negative results in Ames, chromosomal aberration, and forward mutation assays, as well as in vivo DNA synthesis assays in rats, concluding no genotoxic or mutagenic risk.
EFSA (2005) found ATBC non-mutagenic in multiple bacterial and mammalian cell systems (Ames, HPRT, chromosomal aberration), though one mouse lymphoma tk assay was positive. This inconsistency was resolved by a negative in vivo unscheduled DNA synthesis (UDS) test, leading to an overall non-genotoxic interpretation.
U.S. EPA (2014) described multiple in vitro assays (Ames, mouse lymphoma, CHO/HGPRT, and chromosomal aberration tests) as negative, concluding ATBC does not induce mutations or chromosomal damage.
University of Cincinnati (2018) confirmed negative Ames, HPRT, chromosomal aberration, and micronucleus results, with findings meeting OECD or equivalent guidelines, supporting the absence of mutagenic or genotoxic potential.
ANSES (2016) reviewed in vitro and in vivo studies (Ames, chromosomal aberration, gene mutation, micronucleus, and dominant lethal assays) and concluded all were negative, reinforcing a consistent absence of genotoxicity across test types.
Across all available sources (CIR, 2019; EFSA, 2005; U.S. EPA, 2014; University of Cincinnati, 2018; ANSES, 2016), the genotoxicity and mutagenicity calls for Acetyl Tributyl Citrate are consistently negative. Despite one isolated positive mouse lymphoma result (EFSA, 2005), all in vivo and confirmatory data indicate no genotoxic concern.
Summaries of genotoxicity studies
Across all available in vitro and in vivo studies, Acetyl Tributyl Citrate (ATBC; CAS 77-90-7) demonstrated no evidence of mutagenicity or genotoxicity.
In Ames tests, performed with *Salmonella typhimurium* strains (TA98, TA100, TA1535, TA1537, TA1538, and *E. coli* WP2 uvrA), all sources — including CIR (2019), EFSA (2005), U.S. EPA (2014), University of Cincinnati (2018), and ANSES (2016) — reported negative results with and without metabolic activation (S9). Test concentrations ranged from 50 to 10,000 µg/plate, with no increase in revertant colonies or cytotoxicity observed.
In mammalian cell assays, multiple independent studies (CIR, 2019; EFSA, 2005; U.S. EPA, 2014; University of Cincinnati, 2018; ANSES, 2016) showed negative results for both the HPRT gene mutation and mouse lymphoma TK assays. EFSA (2005) noted one inconsistent positive mouse lymphoma result, but this was not reproducible and was contradicted by other negative tests.
Chromosomal aberration and micronucleus assays in mammalian cells (rat lymphocytes, CHO cells, human lymphocytes) were consistently negative across all documents (CIR, 2019; EFSA, 2005; U.S. EPA, 2014; University of Cincinnati, 2018; ANSES, 2016).
In vivo studies confirmed these results. CIR (2019) and EFSA (2005) reported negative outcomes in rat liver unscheduled DNA synthesis (UDS) assays, indicating no DNA damage. ANSES (2016) documented negative results in both a micronucleus assay (rodent bone marrow) and a dominant lethal test, showing no clastogenic or mutagenic effects in vivo.
Collectively, all studies, including those performed under OECD guideline-like conditions, consistently reported no genotoxic or mutagenic activity for ATBC.
Citations: CIR, 2019; EFSA, 2005; U.S. EPA, 2014; University of Cincinnati, 2018; ANSES, 2016.
AI-assisted expert judgment
Based on expert evaluation of the evidence provided, the mutagenicity call for Acetyl Tributyl Citrate is negative. Multiple Ames tests across independent laboratories and regulatory reviews found no mutagenic activity, and mammalian cell assays (HPRT, TK) were consistently negative except for a single unconfirmed positive (EFSA, 2005).
The overall genotoxicity call is negative. In vivo assays, including UDS, micronucleus, and dominant lethal tests, were all negative, confirming the absence of genotoxic potential. The consistency of results across bacterial, mammalian, and whole-animal systems, many performed to OECD standards, supports the conclusion that ATBC is not genotoxic or mutagenic under the conditions tested. This assessment is based on expert judgement using only the information explicitly provided in the referenced data sources (CIR, 2019; EFSA, 2005; U.S. EPA, 2014; University of Cincinnati, 2018; ANSES, 2016).

Systemic Toxicity – summarized evidence (example)

Oral ≤1 Month
A 14-day dietary range-finder in rats reported transient body-weight reduction at 1000 mg/kg/day and hepatic eosinophilia/glycogen decrease at ≥2700 mg/kg/day; no NOAEL was stated [French_tributyl_Tox6]. In CD-1 mice dosed for 15 days, 5 mg/kg/day slightly increased relative spleen weight; 10 mg/kg/day decreased ovarian follicle counts; no systemic threshold was specified [ED_Rasmussen_Tox1].
Lowest systemic threshold reported in this group: not stated. Studies: [French_tributyl_Tox6], [ED_Rasmussen_Tox1].
Oral >1–4 Months
Multiple 6–13-week dietary studies in rats reported no effects at 100 mg/kg/day but liver/kidney weight changes, hepatic hypertrophy and weak peroxisome proliferation at ≥300–1000 mg/kg/day, often described as adaptive (enzyme induction/peroxisome proliferation) [CIR_AcetylTrialkylCitrates_Tox1; EPA_Tox1; EPA_Tox2; French_tributyl_Tox1;

French_tributyl_Tox2; French_tributyl_Tox5; EPA_Toxicity Review of ATBC_2018_Tox1; EPA_Toxicity Review of ATBC_2018_Tox2]. Shorter 6–8-week high-dose screens found diarrhea and reduced gain at ~5000–15 240 mg/kg/day [EPA_Tox3; EPA_Tox4; EPA_Toxicity Review of ATBC_2018_Tox3]. A 2-month cat gavage study (≈5250 mg/kg/day) caused diarrhea and ~30% weight loss [EPA_Toxicity Review of ATBC_2018_Tox6]. Conflicting NOAELs were reported: 100 mg/kg/day (males) [CIR_AcetylTrialkylCitrates_Tox1], 300 mg/kg/day [EPA_Tox1; EPA_Tox2; French_tributyl_Tox1], and 1000 mg/kg/day [EPA_Toxicity Review of ATBC_2018_Tox2; French_tributyl_Tox5].

Lowest systemic threshold reported in this group: NOAEL 100 mg/kg/day (male rats; hepatic/renal changes at higher doses deemed adaptive) [CIR_AcetylTrialkylCitrates_Tox1]. Supporting studies: [CIR_AcetylTrialkylCitrates_Tox1; French_tributyl_Tox1; French_tributyl_Tox2; French_tributyl_Tox5; EPA_Tox1; EPA_Tox2; EPA_Tox3; EPA_Tox4; EPA_Toxicity Review of ATBC_2018_Tox1; EPA_Toxicity Review of ATBC_2018_Tox2; EPA_Toxicity Review of ATBC_2018_Tox3; EPA_Toxicity Review of ATBC_2018_Tox6].

Oral >4 Months–1 Year

A 1-year dietary study in rats and mice (50 or 250 mg/kg/day) noted transient weight and hematology changes at 250 mg/kg/day; NOAEL (systemic) 250 mg/kg/day was reported [EPA_Toxicity Review of ATBC_2018_Tox9].

Lowest systemic threshold reported in this group: NOAEL 250 mg/kg/day [EPA_Toxicity Review of ATBC_2018_Tox9]. Supporting studies: [EPA_Toxicity Review of ATBC_2018_Tox9].

Oral >1 Year

Two-year dietary studies in Wistar and Sherman rats observed slight body-weight decreases and increased liver weights with hepatocellular hypertrophy at ≥300–1000 mg/kg/day; single-cell liver necrosis occurred at 1000 mg/kg/day (7/50 males, 1/50 female). Liver changes were commonly described as adaptive; carcinogenicity was not observed [CIR_AcetylTrialkylCitrates_Tox4; EFSA Journal_Tox2; EPA_Tox5; EPA_Toxicity Review of ATBC_2018_Tox4; EPA_Toxicity Review of ATBC_2018_Tox5; French_tributyl_Tox3; French_tributyl_Tox4]. Reported systemic NOAELs vary: 300 mg/kg/day [CIR_AcetylTrialkylCitrates_Tox4; EPA_Toxicity Review of ATBC_2018_Tox4; French_tributyl_Tox3] vs 1000 mg/kg/day [EPA_Tox5; EPA_Toxicity Review of ATBC_2018_Tox5; French_tributyl_Tox4].

Lowest systemic threshold reported in this group: NOAEL 300 mg/kg/day [CIR_AcetylTrialkylCitrates_Tox4; EPA_Toxicity Review of ATBC_2018_Tox4; French_tributyl_Tox3]. Supporting studies: [CIR_AcetylTrialkylCitrates_Tox4; EPA_Toxicity Review of ATBC_2018_Tox4; French_tributyl_Tox3; EFSA Journal_Tox2; EPA_Tox5; EPA_Toxicity Review of ATBC_2018_Tox5; French_tributyl_Tox4].

Oral Multigeneration

Two-generation dietary studies reported reduced F1 male body weight at 300 mg/kg/day (LOAEL), with systemic NOAEL 100 mg/kg/day for F1 males; no reproductive performance effects up to 1000 mg/kg/day [EPA_Tox6; French_tributyl_Tox7]. EFSA summarized mild body-weight and biochemical changes with a systemic/reproductive NOAEL of 100 mg/kg/day [EFSA Journal_Tox3].

Lowest systemic threshold reported in this group: NOAEL 100 mg/kg/day [EPA_Tox6; French_tributyl_Tox7; EFSA Journal_Tox3]. Supporting studies: [EPA_Tox6; French_tributyl_Tox7; EFSA Journal_Tox3].

Parenteral ≤1 Month

Daily intraperitoneal dosing for 14 days (450–900 mg/kg/day) in mice and rabbits decreased body-weight gain and erythroid indices without liver, lung, or kidney histopathology; bone marrow smears showed no aplasia. No thresholds were reported [French_tributyl_Tox10].

Lowest systemic threshold reported in this group: not stated. Studies: [French_tributyl_Tox10].

Dermal Multigeneration

Guinea pigs with repeated dermal applications (250–500 mg/kg; duration not stated) showed decreased body weight, increased relative liver weight, and reduced cerebral perfusion pressure; no thresholds were reported [French_tributyl_Tox9].

Lowest systemic threshold reported in this group: not stated. Studies: [French_tributyl_Tox9].

Reproductive and developmental toxicity – summarized evidence (example)

Across the available documents, a consistent conclusion is that Acetyl Tributyl Citrate (ATBC; Tri-n-butyl acetyl citrate, CAS 77-90-7) shows no adverse reproductive or developmental effects in standard multi-generation oral studies in rats, with mild or no systemic effects at high dietary concentrations. Multiple sources—EFSA (2005), CSTEE (2004), U.S. EPA (2014), University of Cincinnati (2018), ANSES (2016), and CIR (2019)—agree that ATBC is not of concern for reproductive or developmental toxicity at tested doses.

Key in vivo evidence comes from two-generation and repeated-dose dietary studies in rats. In the study by Robbins (1994), cited by U.S. EPA (2014), University of Cincinnati (2018), and ANSES (2016), Sprague-Dawley rats exposed to 0, 100, 300, or 1000 mg/kg bw/day ATBC showed reduced F1 male body weight at 300 mg/kg/day and maternal weight decreases at 1000 mg/kg/day, but no reproductive or developmental abnormalities. The NOAEL (systemic) was 100 mg/kg/day, NOAEL (reproductive/developmental) = 1000 mg/kg/day.

In the 13-week oral dietary study with in utero exposure in Han Wistar rats (Chase & Willoughby, 2002, cited by CIR (2019) and CSTEE (2004)), no effects on fertility, gestation, parturition, or offspring development were observed up to 1000 mg/kg/day; NOAEL = 300 mg/kg/day (reproductive) and 1000 mg/kg/day (developmental).

Other rat and mouse studies reported similar outcomes (Larionov & Cherkasova 1977, NOAEL = 250 mg/kg/day).

The lowest threshold across documents is 10 mg/kg/day (oral, CD-1 mice) for decreased ovarian follicle counts in Rasmussen et al. (2017), cited in CIR (2019) and University of Cincinnati (2018). This short-term study (15 days) found follicular depletion without fertility or developmental effects.

Mechanistically, CIR (2019), University of Cincinnati (2018), and ANSES (2016) note CYP3A induction and activation of the steroid and xenobiotic receptor (SXR/PXR) in rats and humans (Takeshita et al., 2011), but these were not linked to adverse reproductive outcomes. No conflicts among regulatory bodies were reported regarding reproductive or developmental hazard classification.
(CIR, 2019; EFSA, 2005; U.S. EPA, 2014; University of Cincinnati, 2018; CSTEE, 2004; ANSES, 2016; Rasmussen et al., 2017)

S.4.4. Expert interpretation

AI-assisted point of departure identification – example

Basis: Expert judgment using only the information provided above.

- Oral — lowest available systemic threshold: NOAEL 100 mg/kg/day. Subchronic dietary studies in rats (13 weeks) reported no adverse effects at 100 mg/kg/day, with hepatic/renal changes at ≥300–1000 mg/kg/day commonly interpreted as adaptive (enzyme induction/peroxisome proliferation). Sex-specific NOAELs of 100 mg/kg/day (males) and 300 mg/kg/day (females) were reported; multiple evaluations also cite 100 mg/kg/day from 90-day or multigeneration designs [CIR_AcetylTrialkylCitrates_Tox1; EFSA Journal_Tox1; EU_Tox1; EU_Tox2; EPA_Tox6; French_tributyl_Tox1; French_tributyl_Tox2; French_tributyl_Tox5].

Rationale: Where multiple systemic thresholds were presented for comparable oral studies, I selected the lowest explicitly reported value (100 mg/kg/day) to represent a conservative systemic point of departure, consistent with several evaluations, while noting author interpretations that hepatic/renal changes at higher doses were adaptive rather than adverse. For routes lacking stated thresholds in the provided evidence, I report that explicitly and refrain from inferring values.
